# Obesity genetics in mouse and human: back and forth, and back again

**DOI:** 10.7717/peerj.856

**Published:** 2015-03-24

**Authors:** Fereshteh T. Yazdi, Susanne M. Clee, David Meyre

**Affiliations:** 1 Department of Clinical Epidemiology and Biostatistics, McMaster University, Hamilton, ON, Canada; 2 Department of Cellular and Physiological Sciences, Life Sciences Institute, University of British Columbia, Vancouver, BC, Canada; 3 Department of Pathology and Molecular Medicine, McMaster University, Hamilton, ON, Canada

**Keywords:** Polygenic obesity, Monogenic obesity, Genetics, Integrative biology, Human, Mouse, Genome-wide association study, Next generation sequencing, Knock-out, Transgenic

## Abstract

Obesity is a major public health concern. This condition results from a constant and complex interplay between predisposing genes and environmental stimuli. Current attempts to manage obesity have been moderately effective and a better understanding of the etiology of obesity is required for the development of more successful and personalized prevention and treatment options. To that effect, mouse models have been an essential tool in expanding our understanding of obesity, due to the availability of their complete genome sequence, genetically identified and defined strains, various tools for genetic manipulation and the accessibility of target tissues for obesity that are not easily attainable from humans. Our knowledge of monogenic obesity in humans greatly benefited from the mouse obesity genetics field. Genes underlying highly penetrant forms of monogenic obesity are part of the leptin-melanocortin pathway in the hypothalamus. Recently, hypothesis-generating genome-wide association studies for polygenic obesity traits in humans have led to the identification of 119 common gene variants with modest effect, most of them having an unknown function. These discoveries have led to novel animal models and have illuminated new biologic pathways. Integrated mouse-human genetic approaches have firmly established new obesity candidate genes. Innovative strategies recently developed by scientists are described in this review to accelerate the identification of causal genes and deepen our understanding of obesity etiology. An exhaustive dissection of the molecular roots of obesity may ultimately help to tackle the growing obesity epidemic worldwide.

## Introduction

Obesity is a worldwide epidemic affecting over 400 million adults (World Health Organization, 2011), and is defined as the accumulation of excess body fat to the extent that it results in other health complications and reduces life expectancy (Bahreini et al., 2013). Co-morbidities associated with obesity include psychological distress, osteoarthritis, type 2 diabetes mellitus, hypertension, hyperlipidemia, liver steatosis, cardiovascular disease and certain types of cancer (Switzer, Mangat & Karmali, 2013). The steady increase in life expectancy due to advanced medical treatment may be reversed by negative impacts of obesity on youth today in Westernized countries (Olshanky et al., 2005).

Unfortunately, attempts to prevent obesity have had limited success thus far. The individualistic lifestyle approach of “eat less, move more” has been ineffective as a preventive measure for obesity (Kirk et al., 2012). Effective obesity prevention and management is dependent on the acknowledgement that obesity goes beyond the individual behavior and is influenced by genetics, psychology, society and public policy (Kirk, Penney & McHugh, 2010).

The rise of obesity coincided with several major societal and environmental changes that are likely causal factors. The umbrella term ‘obesogenic’ has been applied to these changes, which include excessive consumption of energy dense foods, sedentary lifestyles, urbanization, and socioeconomic-dependent access to a healthy diet (Hill, 1998; Misra & Khurana, 2008; Must et al., 1999). Aside from environmental factors, considerable evidence from twin, adoption, and family studies indicates that 40 to 70% of BMI variation is due to genetic factors (Choquet & Meyre, 2011). Obesity is a heritable neurobehavioral condition that is highly sensitive to the environment (Ahmad, Varga & Franks, 2013; O’Rahilly & Farooqi, 2008). Understanding the molecular roots of obesity is an important prerequisite to improve both prevention and management of this condition (Bessesen, 2008). This has prompted considerable effort to identify the genes predisposing to obesity by conducting studies in rodents and humans. This review summarizes the progress in the elucidation of obesity genes focusing on the synergies developed between mouse and human obesity genetic fields. The paper also reviews the innovative strategies that synergize the two disciplines to comprehensively uncover the genetic architecture of obesity.

## What Have Mouse Models Taught us About Human Obesity?

Mouse models are the most common experimental animals in obesity genetics. General advantages of mouse models include: low maintenance cost, small size, ease of breeding and short gestation period. They reach sexual maturity faster than other mammals and have a shorter life span (Lee & Cox, 2011; Rosenthal & Brown, 2007). The availability of genetically defined strains, the complete genome sequence of numerous strains, dense single nucleotide polymorphism (SNP) characterization of many others, and well-developed genetic manipulation tools facilitate sophisticated genetic analyses. Genetic manipulation techniques employed in mouse models, such as knock-out/knock-in, overexpression or tissue-specific expression methods can be used to test the functionality of genes associated with obesity (Cox & Church, 2011). Additionally, since homozygote null mutations are exceptionally rare in humans, knock-out mouse models are an attractive alternative to study such rare occurrences (Rees & Alcolado, 2005). Mice also exhibit obesity and metabolic phenotypes that are comparable to humans and can be measured with standardized diagnostic tests (Toye et al., 2004). More detailed phenotyping, such as direct metabolic measurements and assessment of body fat content, that are difficult and costly in large numbers of humans are also possible in mice (Butler & Kozak, 2010; Ellacott et al., 2010; Kaiyala et al., 2010). Importantly, environmental factors can be carefully controlled and specifically manipulated in mouse models (Ayala et al., 2010). Reducing environmental heterogeneity results in an increased power to link genetic variation to the phenotypic differences observed. Specific environmental manipulations allow direct testing of hypotheses to inform about gene-environment interactions. Mice also provide obesity-related tissues such as brain tissue that are otherwise difficult to obtain in humans (Tung et al., 2010).

However, it should be noted that mouse models of obesity are not without their limitations. Different conclusions may arise in mouse and human because of the use of different phenotypes in the study. For instance, BMI measurements are typical in human studies, whereas the direct measurement of percent body fat or body fat mass is more common in mouse models. Unlike humans, there is no defined threshold for obesity based on BMI in mice. Also, in comparison to humans, the secondary complications of obesity substantially depend upon the background strain (Clee & Attie, 2007). Moreover, some physiological differences between humans and mice make studying certain important genes or pathways difficult. For example, the role of *β*-*MSH* in control of energy balance was overlooked in humans mainly due to the fact that mouse models lack *β*-*MSH* (Lee et al., 2006).

## Tools and Approaches Available in Mouse and Human

### Human genetics approaches

#### Linkage analysis

This approach aims to map the location of a disease causing loci by looking for genetic markers that co-segregate with the disease within pedigrees (Teare et al., 2005). Different linkage approaches are applied depending on the type of the disease or trait. For example, parametric analysis is used if the disease is a Mendelian disease (Li & Meyre, 2014).

#### Homozygosity mapping

This is a powerful method to map genes responsible for recessive Mendelian disorders in consanguineous pedigrees. This approach requires less than a dozen of affected individuals, and no additional family members are required to identify the disease causing locus (Lander & Botstein, 1987).

#### Candidate gene studies

Candidate gene approach is hypothesis-driven and has been widely used before the rise of GWAS. Candidate genes have a known biological function that directly or indirectly influence the trait being investigated (Zhu & Zhao, 2007). The main disadvantage of this approach is that it is heavily reliant on the current level of knowledge of a specific gene (Hirschhorn et al., 2002). Candidate genes also have a low success rate overall, as consistent associations have been reported only for a selected few candidate genes (Vimaleswaran et al., 2012).

#### Genome-wide association studies

This approach exhaustively tests the genotype/phenotype associations across up to 4.8 million genetic markers and to date represents the most efficient way to identify common variants (MAF>1%) associated with complex diseases (Visscher et al., 2012).

#### Whole exome/whole genome sequencing

This relatively new approach is efficiently applied to identify rare variants associated with Mendelian or complex traits for a reasonable cost in comparison to classical approaches such as Sanger sequencing. It is powerful because it detects mutations in novel genes not previously detected by candidate gene approaches. The main challenge is to identify a causal gene analyzing the large sequencing dataset (Li & Meyre, 2014). With advances in sequencing technology, it is now possible to sequence approximately 95% of all protein-coding bases of all known genes (the “exome”) at a cost that is comparable to sequencing a single gene by the Sanger method (Shendure, 2011; Singleton, 2011). Despite the fact that whole-genome sequencing experiments are more expensive than whole-exome sequencing experiments, they are more and more used to identify genetic variants associated with Mendelian and complex traits (Morrison et al., 2013; Styrkarsdottir et al., 2014).

### Mouse genetic approaches

#### Natural mutations

Naturally occurring mutations are spontaneous mutations in mice that could be linked to the trait of interest. Natural mutations can range from simple single nucleotide substitution to complex rearrangements (Justice, Siracusa & Stewart, 2011). They occur by chance and transmission from parent to offspring results in fixation of these mutations within a population (Justice, Siracusa & Stewart, 2011). These mutations are often studied by quantitative trait loci (QTLs), which link a chromosomal region to the trait of interest (Chiu et al., 2006; Diament, Fisler & Warden, 2003).

Although studying natural variants may be appealing, regrettably the spontaneity of their appearance is often matched by their impromptu disappearance (Stanford, Cohn & Cordes, 2001). Furthermore, studying obesity genes in mouse models with natural mutations may be a more time consuming approach compared to chemically induced mutations.

#### Chemically induced mutations

Chemical mutagenesis increases frequency and variety of mutations for functional genetic studies. Furthermore, with the use of inbreeding techniques, chemical mutagenesis can create a genetic variant that is identical to parent strain except for the induced mutation that may be responsible for phenotypic diversity compared to parent strain (Svenson, Bogue & Peters, 2003). This approach creates a set of mutants that differ minimally in genotype from parental strain, but differ robustly in phenotype, making it a promising approach in functional genetic studies (Svenson, Bogue & Peters, 2003).

Successful genetic manipulation requires DNA modifications of germ-line cells so that the modification is heritable (Strachan & Read, 1999). Target cells usually can differentiate into different cells or give rise to germ-line cells, which makes embryonic stem cells (ES) ideal because they can differentiate to somatic and germ line cells (Strachan & Read, 1999).

#### X-ray mutagenesis

This method induces mutations 20–100 times greater than spontaneous mutations. It causes chromosomal rearrangements which can range from simple deletions, inversions and translocations to complex rearrangements (Silver, 1995). In this approach, several genes are affected by chromosomal rearrangements, therefore this method adds complexity to genetic studies and makes it difficult to dissect individual gene function (Stanford, Cohn & Cordes, 2001).

#### Chlorambucil

Chlorambucil (CAB) induces similar chromosomal translocations and multigene deletions to X-ray (Russell et al., 1989). CAB is an alkalyting agent that impacts cell division and results in aneugenic activity (Efthimiou et al., 2007). CAB causes smaller deletions and translocations in comparison to X-ray mutagenesis, but it does not lead to single-gene identification and therefore, is not used in high-throughput approaches (Stanford, Cohn & Cordes, 2001).

#### Ethylnitrosourea

Ethylnitrosourea (ENU) is an alkylating agent that induces point mutations in the DNA of spermatogonial stem cells *via* single-base mismatching to the unrepaired alkylated base (Svenson, Bogue & Peters, 2003). ENU is advantageous since it is easy to administer, results in higher mutagenesis rate and is amendable to high-throughput screening (Justice, 2000). Through international collaborative efforts several archives of DNA, embryonic stem (ES) cells or typically sperm, from mutagenized mice have been created and catalogued along with some standardized phenotypic data and the listing of mutations they contain. The corresponding ES cells/sperm can be ordered and used to regenerate mice harboring the mutation of interest (Acevedo-Arozena et al., 2008).

### Insertional mutagenesis

#### Pronuclear injection

This approach involves microinjection of DNA into fertilized oocytes to affect the function of an endogenous gene. This approach requires the cloning of cDNA (coding sequence of a selected gene). The sequence is then inserted in frame with a constitutively active promoter that drives transcription (Gordon & Ruddle, 1981; Harbers, Jahner & Jaenisch, 1981). This method disturbs endogenous gene expression and can generate chromosomal rearrangements and deletions (Belizário et al., 2012). Pronuclear injections are labor intensive and highly technical, thus they are not used in high-throughput screening (Stanford, Cohn & Cordes, 2001).

#### Gene targeting

Targeted mutagenesis by homologous recombination in embryonic stem cells is used to efficiently target a single gene (Sun, Abil & Zhao, 2012). Gene targeting was first pioneered by Thomas & Capecchi (1987) who were able to exchange the endogenous gene with a mutated copy in cultured mammalian cells by using a homologous sequence of the gene. This approach is able to produce specific alterations to the mouse genome to analyze targeted gene function (Menke, 2013).

#### Gene trapping

Gene trapping is a vector insertion that disrupts the regular transcription of endogenous genes (O’Kane & Gehrin, 1987). Gene trapping includes enhancer, promoter and exon traps. The enhancer trap is used for gene identification, and it involves the introduction of a reporter construct that requires a *cis-*acting DNA to activate gene expression. Genes are then identified depending on the expression information (Yamamura & Araki, 2008). The promoter and the exon traps are mainly used for mutagenesis. The promoter trap contains the coding sequence of the reporter gene and can interfere with normal coding capacity of endogenous genes and create a mutation. The exon trap is designed to create spliced fusion transcripts between the reporter and the endogenous gene (Yamamura & Araki, 2008). Gene trapping is an efficient system for simultaneous studies of gene function, sequence and expression (Stanford, Cohn & Cordes, 2001). Many of the targeted ES cells produced by the international consortia include gene-trap vectors.

#### Lentivirus vectors

Viral vectors are a stable, long-term gene delivery system of genetic information to host cells. The system depends on replicating viruses that have the genetic information for the targeted gene instead of their own coding regions (Kootstra & Verma, 2003). Lentiviral vectors are often preferred over other viral vectors because they are more efficient at delivering complex gene expression cassettes (May et al., 2000), they can mediate long term gene expression (Seppen et al., 2001) and they are relatively safe (Brown et al., 2010). They provide high control over the manipulated gene, and are ideal for studying gene function in small populations (Osten, Dittgen & Licznerski, 2006). Other viral vectors that are commonly used, particularly adeno-associated virus (AAV) vectors, have different tissue tropisms that can be used for the over-expression of genes of interest even within the central nervous system, that may be of particular relevance for obesity (Lentz, Gray & Samulski, 2012). The development of novel viral vectors is ongoing (Huang & Kamihira, 2013). Recent advances in materials and nanotechnologies may also facilitate non-viral methods of direct gene delivery (Yin et al., 2014), although these are not yet routinely used.

### Inbreeding methods

Once a mutation is successfully induced, a mutant model is obtained and the next step is to dissect the genetic roots of the phenotype. Figure 3 is a graphical representation of different inbreeding techniques (Rogner & Avner, 2003). This section provides an overview of the different inbreeding approaches used to reach this objective.

#### Genetic cross

Genetic cross is a classical cross where two inbred strains are mated and their offspring are either mated to each other (an intercross F2 design) or to a progenitor strain (a backcross design) (Flint & Eskin, 2013). Second-generation offspring are then phenotyped and genotyped, and linkage analysis is carried out to identify a region that is associated with the trait of interest (Silver, 1995).

#### The classical inbred strains

This method provides a higher mapping resolution than the genetic cross, since the inbred mouse strains are separated from their founders by more generations, thus increasing the recombination events between the genomes of the founding strains (McClurg et al., 2007). These strains are commercially available for purchase from vendors, and no breeding steps are required in this approach.

#### The recombinant inbred lines

Recombinant inbred lines are created through cross breeding of inbred mice, amplifying the genotypic/phenotypic diversity found across inbred mice. The power of recombinant inbred lines are in that they represent a fixed polygenic model that can be phenotyped deeply, in multiple environments (Zou et al., 2005). This fixed model is established *via* crossing two different inbred parental strains to produce F1 offspring, and then generating a series of brother-sister mating for at least 20 generations. This produces fully inbred strains which are homozygous at all loci for a unique combination of the original parental genomes (Pomp, 2007). An example of recombinant inbred lines is the Hybrid Mouse Diversity Panel (HMDP). This is a panel of approximately 100 strains that are phenotyped and association is carried out after correcting for population structure using efficient mixed-model association and made available to the scientific community. The combined populations in the HMDP provide a high statistical power and a high resolution (Flint & Eskin, 2013), which makes this model ideal for systems-level analysis of gene by environment interactions (Parks et al., 2013).

Another illustration of recombinant lines is the Collaborative Cross, which is a large-scale effort to create a set of recombinant inbred strains that are specifically designed for mapping traits (Aylor et al., 2011). By using wild derived strains, a substantial amount of genetic diversity is introduced, giving the collaborative cross the advantage of covering more genetic variations compared to other approaches (Philip et al., 2011).

#### Chromosome substitution strains or consomic strains

Chromosome substitution occurs when a single, full-length chromosome from one inbred strain has been transferred onto the genetic background of a second strain by repeated backcrossing (Nadeau et al., 2000). The method involves construction of chromosome substitution strains (CSS) between a donor and a host strain, which partitions the variation between two strains and becomes a resource for studying genetic control of phenotype variation (Singer et al., 2004).

#### Long-term selection lines

Long-term selection lines are developed through selective breeding for a wide variety of phenotypes (Paigen, 2003). Several of these lines can be joint and inbred to characterize different metabolic traits together and develop models for gene mapping. For example, the body fat phenotype in mice were developed by long-term selection of fat (F) and lean (L) mice in over 60 generations (Horvat et al., 2000). A genome-wide quantitative trait locus (QTL) analysis of a cross between F and L lines revealed QTLs that mapped to regions that were previously described as obesity QTLs (Horvat et al., 2000).

#### The heterogeneous stock

The heterogeneous stock (HS) uses outbred mice to increase statistical power compared to recombinant inbred strains. The outbred mice are similar to F2 animals from a cross, but they have ancestry from eight founder strains instead of only two, and the population is bred for more generations (Valdar et al., 2006). Commercial outbred stock animals have been maintained for many generations, and they provide high-resolution mapping (Flint & Eskin, 2013).

### Genetic manipulations in mice

Gene manipulation techniques allow for definitive alterations of specific genes at the systemic level. They also enable gene alterations in a time or tissue specific manner (Speakman et al., 2007).

#### Over-expression of target genes

This method involves cloning of a full-length coding sequence downstream of a promoter, which may provide a global or tissue-specific overexpression of a target gene in the transgenic offspring (Speakman et al., 2007). This technique is straightforward and inexpensive, but the extent of changes in gene and protein expression is not always predictable. It can also disturb endogenous gene expression (Justice, Siracusa & Stewart, 2011; Stanford, Cohn & Cordes, 2001).

#### Knock-out models

Knock-out models involve the total ablation of the target gene in all tissues, but the ablation of target genes could reveal unpredicted effects (Davey & MacLean, 2006). For example, homozygous knock-outs may result in embryonic death (eg: *Sim1*^−/−^) (Michaud et al., 2001), or developmental compensation (eg: *Npy*) (Erickson, Hollopeter & Palmiter, 1996; Erikson, Clegg & Palmiter, 1996) in which no particular phenotype will be observed. In the example of *Sim1*, heterozygous knock outs of this gene survive, but develop severe obesity associated with increase in food intake without measurable energy expenditure. This is indicative of how *Sim1* plays a role in energy homeostasis (Ramachandrappa et al., 2013).

##### SiRNA/shRNA

Conditional gene knockdown is a powerful tool for studying gene function, and methods of gene knockdowns are in constant evolution (Brown et al., 2010). The discovery of small interfering RNA (siRNA) as a viable mechanism for eliciting RNA interference (RNAi) expanded opportunities for functional genetic studies (Elbashir et al., 2001). Vector directed siRNA technology allows for rapid generation of large number of knockdown mice in any strain of interest (De Souza et al., 2006), which produces strong and specific suppression of gene expression with no cytotoxicity (Elbashir et al., 2001).

Short hair pin RNAs (shRNA) provide a more straightforward approach for down regulation of gene expression, because unlike siRNAs, they can be stably expressed within the cell and are not lost with cell division (Brown et al., 2010). Lentivirus vectors containing polymerase III promoters for shRNA expression and polymerase II promoter for fluorescent protein expression (for labeling the cell) can be used in order to knock-down endogenous genes (Dittgen et al., 2004). An example of this application is silencing of the leptin receptor gene related protein (*OB-RGPR*) that was accomplished *via* lentiviral vector encoding a shRNA directed against *OB-RGPR* in the hypothalamus (Couturier et al., 2007). Silencing of *OB-RGPR* in hypothalamus prevents the development of diet-induced obesity in mice fed high-fat diet (HFD) (Couturier et al., 2007). Expression of microRNAs (miRNA) can also be used to modulate gene expression (Casola, 2010).

#### Knock-in models

These models involve the replacement of the endogenous gene with the mutated form and are used to study more specific roles of the changes in protein function (Speakman et al., 2007). This technique could also be used to confirm the impact of target mutations on phenotype of the disease. For example, humans with dominant negative *PPAR*_*γ*_ L466A mutation display severe insulin resistance, dyslipidemia and hypertension (Freedman et al., 2005). Further studies of the mutation in mice revealed that mice with *Ppar*_*γ*_ knock-in L466A mutation exhibit lipodystrophy, decrease in adipogenic genes, high circulating free fatty acids (FFAs), and low adiponectin. Human studies of this mutation, coupled with the animal experiments confirm the importance of *PPAR*_*γ*_ in adipose tissue maintenance (Freedman et al., 2005)

#### The Cre/loxP system

This is a tool for tissue-specific and time specific knock out genes. When Cre is expressed in mice with a loxP containing gene, the desired gene is excised (Kühn & Torres, 2002). The expression of Cre can be driven either through a transgenic under a tissue specific and/or temporally regulated promoter, or by direct delivery to cells. Depending on the specific tissue or time of Cre expression, modifications can be restricted to a certain cell type or a development stage (Kühn & Torres, 2002). Delivery of Cre *via* viral vectors provides a more specific gene delivery in the nervous system (Kaspar et al., 2002), particularly if performed by targeted injection.

Newer gene expression manipulation techniques such as zinc fingers and TALENs are also used in obesity genetics field, albeit less frequently than Cre/loxP. Zinc fingers are a versatile DNA recognition domain that have been combined in a modular fashion to generate fusion proteins that recognize unique DNA sites in complex eukaryotic genomes (Urnov et al., 2010). Similarly, transcription activator-like effector nuclease (TALENs) from pathogenic bacterium *Xanthomonas* can be engineered to virtually bind to any DNA sequence (Boch, 2011). Each TALEN can travel to the nucleus, bind to the promoters of target genes and induce transcription based on their specific DNA-binding site (Boch, 2011).

## The Golden Age of Mouse Obesity Genetics

### Monogenic obesity mouse models and candidate gene studies in human

**Figure 1:**
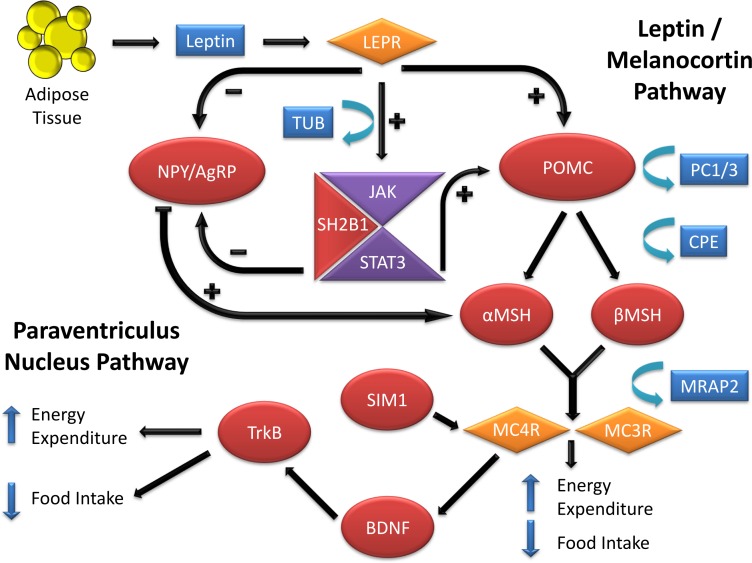
Genes involved in the leptin-melanocortin pathway that have been associated with monogenic obesity through their influence on food intake and energy expenditure. Leptin secreted from adipose tissue binds to the leptin receptor in the hypothalamus. Leptin binding inhibits the neuropeptide Y/agouti-related protein (NPY/AgRP) production and stimulates pro-opiomelanocortin (POMC) production, which undergoes post-translational modifications to produce peptides such alpha and beta-melanocyte-stimulating hormone (*α* and *β*MSH) via the processing of prohormone convertase 1(PC1/3) and carboxypeptidase E (CPE) enzymes. Alpha and *β*MSH bind to melanocortin 3 and melanocortin 4 receptors (MC3R and MC4R) and induce their activity. Melanocortin 2 receptor accessory protein 2 (MRAP2) can reduce the responsiveness of both MC3R and MC4R to *α* and *β*MSH and result in obesity. On the other hand, Single-minded 1 (SIM1) acts as a facilitator of MC4R activity. Increase in the MC3R and MC4R activities result in a decrease in food intake and increase in energy expenditure. MC4R activity also stimulates release of Brain-derived neurotrophic factor (BDNF) which will bind to the neurotrophin receptor (TrkB) and influence food intake and energy expenditure. Aside from activation of the POMC, leptin binding to its receptor also activates the Janus kinase/signal transducer and activator of transcription (JAK/STAT) signaling. This pathway, through the help of Src homology 2 B adapter protein 1 (SH2B1), results in activation of Signal transducer and activator of transcription 3 (STAT3). STAT3 will then migrate to the nucleus with the help of Tubby bipartite transcription factor (TUB) and activate its target genes related to energy homeostasis and mediate in the anorexigenic effects of leptin.

The identification of genes underlying monogenic obesity relied heavily on mouse genetic studies. By searching the available literature on mouse models of obesity, we collected 221 genes that have been linked to obesity or weight gain *via* knock-out or transgenic mice, or by utilizing techniques such as over-expression or Cre/loxP (Table 1). We conducted this literature review by searching key terms in PubMed and OMIM databases. We have focused on the obesity and weight gain phenotype and did not detail genes responsible for leanness phenotypes. Leanness may truly result from the manipulation of a gene important in energy balance (e.g., FTO inactivation leads to leanness, FTO overexpression leads to obesity). However, leanness may also be linked to toxicity and sickness of the animal due to genetic manipulations (Reed, Lawler & Tordoff, 2008). The study of monogenic obesity in mice pioneered our understanding of the mechanisms underlying the regulation of body weight in humans. The genes underlying monogenic forms of obesity in humans all encode members of these highly conserved pathways, which are essential in regulation of body weight and energy homeostasis (Fig. 1) (Choquet & Meyre, 2011; Farooqi & O’Rahilly, 2008). Since a detailed discussion of all the genes listed in Table 1 is not feasible for one review paper, we will focus on a subset of the genes that are all part of the leptin/melanocortin or paraventricular nucleus development pathways in the section below.

**Table 1:** List of genes experimented in mouse models for obesity or obesity-related phenotypes.

Num	Cand. genes	Phenotypic details	Technique	Ref
1	*ACADVL*	Adult-onset fat mass gain	Knock-out	Exil, VJ. 2003. Circ Res
2	*ADAR2*	Obesity under HFD^*^	Transgenic	Singh, M. 2007. J Biol Chem
3	*ADRA1B*	Accelerated weight gain on HFD	Knock-out	Burcelin, R. 2004. J Biol Chem
4	*ADRA2A*	Obesity in homozygous mutation	Transgenic	Valet, P. 2000. J Biol Chem
5	*ADRB1*	Obesity	Knock-out	Bachman, ES. 2002. Science
6	*ADRB2*	Obesity	Knock-out	Soloveva, V. 1997. Mol Endocrinol
7	*ADRB3*	Obesity on HFD	Knock-out	Susulic, VS. 1995. J Biol Chem
8	*AEBP1*	Obesity in females	Transgenic	Zhang, L. 2005. Mol Med
9	*AGRP*	Elevated weight gain & obesity	Transgenic	Ollman, MM. 1997. Science
10	*ALMS1*	Obesity	Knock-out	Collin, GB. 2005. Hum Mol Genet
11	*ALP1*	Accelerated weight gain on HFD	Knock-out	Narisawa, S. 2003. Mol Cell Biol
12	*ANGPTL6*	Obesity and insulin resistance	Knock-out	Oike, Y. 2005. Nat Med
13	*ANKRD26*	Obesity in homozygotes	Transgenic	Bera, TK. 2008. Acad Sci USA
14	*APOB*	Increased BW^*^	Knock-out	Siri, P. 2009. J Biol Chem
15	*APOC3*	Obesity on HFD	Transgenic	Jong, MC. 2001. J Lipid Res
16	*APOE*	Obesity	Knock-out	Zhang, T. 2013. Reproduction
17	*AQP7*	Adult-onset obesity	Knock-out	Hibuse, T. 2005. Proc Natl Acad Sci USA
18	*AR*	Obesity, decreased energy expenditure	Cre/LoxP	Fan, W. 2005. Diabetes
19	*ASIP*	Increased BW & Fat mass – Obesity	Transgenic	Mynatt, RL. 1997. Natl Acad Sci USA
20	*AT2R*	Increase in BW in females only	Knock-out	Samuel, P. 2013. PLoS One
21	*ATX*	Increase in adiposity in fat-specific knockout under HFD	Cre/LoxP	Dusaulcy, R. 2011. J Lipid Res
22	*ATXN2*	Obesity under HFD	Knock-out	Kiehl, T. 2006. Biochem Biophys Res Commun
23	*BBS1*	Adult-onset obesity in 10% of mutants	Knock-out	Kulaga, HM. 2004. Nat Genet
24	*BBS2*	Adult-onset fat mass gain	Knock-out	Nishimura, DY. 2004. Proc Natl Acad Sci USA
25	*BBS4*	Adult-onset obesity	Knock-out	Mykytyn, K. 2004. Proc Natl Acad Sci USA
26	*BBS7*	Obesity	Knock-out	Zhang, Q. 2013. J Cell Sci
27	*BDNF*	Adult-onset obesity in heterozygotes	Knock-out	Coppola, V. 2004. Neuroreport
28	*BRD2*	Obesity	Knock-out	Wang, F. 2009. Biochem J
29	*BRS3*	Obesity	Knock-out	Ohki-Hamazaki, H. 1997. Nature
30	*CAPN10*	Increase in body weight	Knock-out	Cheverud, JM. 2010. J Lipid Res
31	*CART*	Adult-onset obesity	Knock-out	Wierup, N. 2005. Regul Pept
32	*CAV3*	Increased adiposity	Knock-out	Capozza, F. 2005. Am J Physiol Cell Physiol
33	*CB2R*	Increase in body weight and hyperphagia	Knock-out	Agudo, J. 2010. Diabetologia
34	*CCKBR*	Obesity	Knock-out	Lavine, J. 2010. Endocrinol
35	*CDH2*	Increased adiposity	Transgenic	Castro, CH. 2004. J Cell Sci
36	*CDKN1A (B)*	Increased adiposity	Knock-out	Naaz, A. 2004. FASEB J
37	*CEP19*	Obesity	Knock-out	Shalata, A. 2013. Am J Hum Genet
38	*CHEMR23*	Adult-onset obesity	Knock-out	Rouger, L. 2013. J Endocrinol
39	*CHGA*	Increased adiposity	Knock-out	Bandyopadhyay, G. 2012. J Biol Chem
40	*CHOP*	Obesity under HFD	Knock-out	Grant, RW. 2014. J Biol Chem
41	*CLOCK*	Obesity	Knock-out	Turek, F. 2005. Science
42	*CORIN*	Increased bodyweight	Knock-out	Chan, JC. 2005. Proc Natl Acad Sci USA
43	*CPE*	Obesity	Knock-out	Cawley, NX. 2004. Endocrinology
44	*CPT1*	Obesity under HFD	Knock-out	Gao, FX. 2009. Diabetologia
45	*CRH*	Excess fat accumulation & muscle atrophy	Transgenic	Stenzel-Poore, MP. 1992. Endocrinology
46	*CRY (1/2)*	Obesity under HFD	Knock-out	Barclay, JL. 2013. Am J Physiol Endorinol Metab
47	*CSF2*	Adult-onset obesity	Knock-out	Reed, JA. 2005. J Clin Invest
48	*CTRP9*	Increased bodyweight & adiposity	Knock-out	Wei, Z. 2014. Am J Physiol Endocrinol Metab
49	*CYP19A1*	Elevated gonadal fat pad weight	Knock-out	Misso, ML. 2005. Horm Metab Res
50	*D2*	Increased BW & adiposity	Knock-out	Marsili, A. 2011. PLoS One
51	*DGAT1*	Increased gonadal but not subcutaneous fat	over-expression	Yamazaki, T. 2005. J Biol Chem
52	*DPT*	Increased subcutaneous fat	Knock-out	Takeda, U. 2002. J Invest Dermatol
53	*DRD3*	Increased adiposity and obesity	Knock-out	McQuade, JA. 2004. Behav Brain Res
54	*dup(17)*	Obesity	Transgenic	Walz, K. 2003. Mol Cell Biol
55	*ECSCR*	Obesity under HFD	Transgenic	Akakabe, Y. 2013. Nat Commun
56	*ESR1*	Obesity	Knock-out	Heine, PA. 2000. Proc Natl Acad Sci USA
57	*FABP4*	Obesity in homozygotes under HFD	Knock-out	Hotamisligil, GS. 1996. Science
58	*FATP4*	Obesity in homozygotes under HFD	Knock-out	Lenz, LS. 2011. J Biol Chem
59	*FKBP51*	Increase in body weight under HFD	Transgenic	Yang, L. 2012. Am J Physiol Endocrinol Metab
60	*FOXA2*	Heterozygotes develop obesity under HFD	Knock-out	Wolfrum, C. 2003. J Clin Invest
61	*FOXO1*	Obesity	Transgenic	Kamei, Y. 2004. J Biol Chem
62	*FOXO3A*	Obesity	Knock-out	Fang, C. 2008. Am J Physiol
63	*FSHR*	Obesity	Knock-out	Danilovich, N. 2000. Endocrinology
64	*FTO*	Obesity	Over-expression	Church, C. 2010. Nat Genet
65	*GAL-3*	Late-onset obesity	Knock-out	Pang, J. 2013. PLoS One
66	*GAST*	Obesity	Knock-out	Cowey, SL. 2005. Cancer
67	*GCK*	Increased BW under HFD	Transgenic	Ferre, T. 2003. Diabetologia
68	*GDF3*	Increased BW under HFD	Over-expression	Wang, W. 2004. Biochem Biophys Res Commun
69	*GFPT1*	Increased adiposity	Transgenic	McClain, DA. 2005. Am J Physiol Endocrinol Metab
70	*GH*	Obesity	Transgenic	Pomp, D. 1996. Transgenic Res
71	*GHR*	Increased adiposity in males	Knock-in	Rowland, JE. 2005. Mol Cell Bio
72	*GHRH*	Increased adiposity	Transgenic	Cai, A. 1999. Endocrinology
73	*GIRK4*	Increased BW & adiposity	Knock-out	Perry, CA. 2008. Proc Natl Acad Sci USA
74	*GNAS*	Maternal inheritance of mutant allele leads to obesity	Knock-out	Germain-Lee, EL. 2005. Endocrinology
75	*GNB3*	Increased BW and adiposity	Transgenic	Goldlust, S. 2013. Proc Natl Acad Sci USA
76	*GPD2*	Increased BW & adiposity in females	Knock-out	Alfadda, A. 2004. Am J Physiol Regul Integr Comp Physiol
77	*GPR10*	Adult-onset obesity	Knock-out	Ishii, M. 2003. Proc Natl Acad Sci USA
78	*GPR120*	Obesity under HFD	Knock-out	Hirasawa, A. 2005. Nat Med
79	*GPR26*	Obesity	Knock-out	Chen, D. 2012. PLoS One
80	*GPR39*	Obesity	Knock-out	Moechars, D. 2006. Gastroenterology
81	*GPR7*	Adult-onset obesity	Knock-out	Gu, W. 2004. J Mol Neurosci
82	*GPX1*	Increased BW & adiposity	Transgenic	McClung, JP. 2004. Proc Natl Acad Sci USA
83	*GRM8*	Increased adiposity	Knock-out	Duvoisin, RM. 2005. Eur J Neurosci
84	*GRP*	Resistant to diet-induced obesity	Knock-out	Ye, R. 2010. Diabetes
85	*GRPR*	Reduced food intake	Knock-out	Hampton, LL. 1998. Proc Natl Acad Sci USA
86	*GSK3B*	Increased BW & adiposity in males	Transgenic	Pearce, NJ. 2004. Metabolism
87	*HDC*	Increased BW & adiposity	Knock-out	Hara, J. 2001. Neuron
88	*HIF1α*	Obesity	Transgenic	Zhang, X. 2010. J Biol Chem
89	*HRH1*	Late onset obesity	Knock-out	Masaki, T. 2004. Diabetes
90	*HRH3*	Increased BW & adiposity	Knock-out	Takahashi, K. 2002. J Clin Invest
91	*HSD1-11β*	Obesity	Transgenic	Zhang, L. 2012. Transgenic Res
92	*HSD11β2*	Increased adiposity	Transgenic	Masuzaki, H. 2001. Science
93	*HTR2C*	Late onset obesity	Knock-out	Nonogaki, K. 2003. Diabetes
94	*ICAM1*	Late onset obesity/accelerated under HFD	Knock-out	Gregiore, FM. 2002. AM J Physiol Endocrinol Metab
95	*IDH1*	Obesity	Transgenic	Koh, HJ. 2004. J Biol Chem
96	*IFRD1*	Increased adiposity	Transgenic	Wang, Y. 2005. J Biol Chem
97	*IL18*	Increased BW	Knock-out	Netea, M. 2006. Nature Medicine
98	*IL18R*	Increased BW	Transgenic	Netea, M. 2006. Nature Medicine
99	*IL-1RI*	Adult-onset obesity	Knock-out	McGillicuddy, FC. 2013. Am J Physiol Endocrinol Metab
100	*IL6*	Increased BW & adiposity	Knock-out	Wallenius, V. 2002. Nat Med
101	*INSR*	Increased adiposity & obesity	Cre/LoxP	Cariou, B. 2004. Endocrinol
102	*IRS1*	Increase weight gain	Knock-out	Shirakami, A. 2002. J Endocrinol
103	*IRS2*	Increased adiposity	Cre/LoxP	Lin, X. 2004. J Clin Invest
104	*JAK2 (Adipose)*	Increased adiposity	Cre/LoxP	Sy, S. 2014. Diabetalogia
105	*KCNJ11*	Increased BW & adiposity	Knock-out	Kanezaki, Y. 2004. Endocr J
106	*KDM3A*	Obesity	Knock-out	Okada, Y. 2010. J Androl
107	*KRAS*	Obesity under HFD	Transgenic	Dawson, DW. 2013. Cancer Prev Res
108	*KSR2*	Obesity	Knock-out	Revelli, JP. 2011. Obesity
109	*LEP*	Obesity	Knock-out	D’Souza, AM. 2014. Endocrinol
110	*LEPR*	Obesity	Knock-in	Bates, SH. 2003. Nature
111	*LH (B)*	Obesity in females	Transgenic	Kero, JT. 2003. Am J Physiol Endocrinol Metab
112	*LIPC*	Increased adiposity	Knock-out	Farahani, P. 2004. Obes Res
113	*LPIN1*	Obesity due to increased fat storage	Transgenic	Phan, J. 2005. Cell Metab
114	*LRH-1*	Mild obesity	Knock-out	Hattori, T. 2014. Endocr J
115	*LSR*	Obesity in heterozygotes	Transgenic	Yen, FT. 2008. J Biol Chem
116	*MAGEL2*	Increased BW & adiposity	Knock-out	Bischof, JM. 2007. Hum Mol Genet
117	*MAGP-1*	Increased BW & adiposity	Knock-out	Weinbaum, JS. 2008. J Biol Chem
118	*MAS*	Increased adiposity	Knock-out	Santos, SH. 2008. Diabetes
119	*MC3R*	Obesity	Knock-out	Butler, AA. 2000. Endocrinolopgy
120	*MC4R*	Obesity	Knock-out	Huszar, D. 1997. Cell
121	*MED13*	Obesity	Cre/LoxP	Grueter, C. 2012. Cell
122	*MEST*	Increased adiposity	Transgenic	Takahashi, M. 2005. Am J Physiol Endocrinol Metab
123	*MKKS*	Obesity	Knock-out	Fath, MA. 2005. Hum Mol Genet
124	*MMP11*	Obesity	Knock-out	Andarawewa, KL. 2005. Cancer Res
125	*MMP19*	Accelerated weight gain on HFD	Knock-out	Pendas, AM. 2004. Mol Cell Biol
126	*MPO*	Increased BW	Transgenic	Castellani, LW. 2006. J Lipid Res
127	*MRAP2*	Obesity	Knock-out	Asai, M. 2013. Science
128	*MT1A (B)*	Adult-onset obesity	Knock-out	Beattie, JH. 1998. Proc Natl Acad Sci USA
129	*MT-HGH*	Obesity	Transgenic	Wolf, E. 1991. Growth Dev Aging
130	*NBEA*	Increased BW & adiposity in heterozygotes	Knock-out	Olszewski, P. 2012. PLoS Genet
131	*NEIL1*	Obesity	Knock-out	Sampath, H. 2011. Am J Physiol Endocrinol Metab
132	*NEP*	Adult-onset obesity	Knock-out	Becker, M. 2010. PLoS One
133	*NGN3*	Obesity	Knock-out	Anthwal, N. 2013. Dis Model Mech
134	*NHLH2*	Adult-onset obesity	Knock-out	Jing, E. 2004. Endocrinology
135	*NMU*	Increased BW & adiposity	Knock-out	Handa, R. 2004. Nat Med
136	*NPB*	Mild obesity	Knock-out	Kelly, MA. 2005. Proc Natl Acad Sci USA
137	*NPC1*	Dose-dependent weight gain under HFD	Knock-out	Jelinek, D. 2010. Obesity
138	*NPY*	Obesity under high-sucrose diet	Transgenic	Kaga, T. 2001. Diabetes
139	*NPY1R*	Obesity	Knock-out	Kushi, A. 1998. Proc Natl Acad Sci USA
140	*NPY2R*	Obesity	Knock-out	Lin, D. 2006. Endocrinol
141	*NPY5R*	Increased adiposity	Knock-out	Marsh, DJ. 1998. Nat Med
142	*NR5A1*	Adult-onset obesity	Knock-out	Majdic, G. 2002. Endocrinol
143	*NTSR1*	Adult-onset obesity	Knock-out	Remaury, A. 2002. Brain Res
144	*OGG1*	Increased adiposity in HFD	Knock-out	Sampath, H. 2012. PLoS One
145	*OMA1*	Obesity	Knock-out	Quiros, PM. 2012. EMBO
146	*OSMRβ*	Increase in BW and hyperphagia	Knock-out	Gotardo, EM. 2013. J Nutr Sci Vitaminol
147	*OXT*	Obesity	Knock-out	Nishimori, K. 2008. Prog Brain Res
148	*P62*	Adult-onset obesity and hyperphagia	Knock-out	Harada, H. 2013. J Neurosci
149	*PARP1*	Adult-onset obesity	Knock-out	Devalaraja-Narashimha, K. 2010. J Endocrinol
150	*PC1/3*	Increased adiposity in heterozygotes	Knock-out	Zhu, X. 2002. Proc Natl Acad Sci USA
151	*PCK1*	Obesity	Transgenic	Franckhauser, S. 2002. Diabetes
152	*PCSKIN*	Adult-onset obesity	Transgenic	Wei, S. 2004. J Endocrinol
153	*PCYT2*	Obesity	Knock-out	Fullerton, MD. 2009. J Biol Chem
154	*PEG3*	Obesity	Knock-out	Curley, JP. 2005. FASEB J
155	*PGC-1α*	Obesity	Knock-out	Leone, TC. 2005. PLoS Biol
156	*PGDS*	Obesity	Knock-out	Tanaka, R. 2009. Biochem Biophys Res Commun
157	*PGP*	Increased BW & adiposity	Knock-out	Foucaud-Vignault, M. 2011. PLoS One
158	*PHB*	Obesity	Transgenic	Ande, SR. 2014. Diabetes
159	*PI3K (p110α*)	Increased adiposity, hyperphagia	Knock-in	Foukas, L. 2006. Nature
160	*PLAC 8*	Increase in adiposity	Knock-out	Jimenez-Preitner, M. 2011. Cell Metab
161	*PLSCR1*	Increased adiposity	Knock-out	Zhou, Q. 2002. Blood
162	*PLSCR3*	Increased BW & adiposity	gene-trap	Wiedmer, T. 2004. Proc Natl Acad Sci USA
163	*POMC*	Obesity under HFD	Knock-out	Challis, BG. 2004. Proc Natl Acad Sci USA
164	*PPARA*	Increase in adiposity	Knock-out	Miyazaki, M. 2004. J Biol Chem
165	*PPARG2*	Obesity under HFD	Knock-in	Heikkinen, S. 2009. Cell Metab
166	*PPARGC1A*	Increased adiposity in young females & old males	Knock-out	Leone, TC. 2005. PLoS Biol
167	*PPARδ*	Obesity under HFD	Knock-out	Kocalis, H. 2012. PLoS One
168	*PPIF*	Late-onset obesity	Knock-out	Luvisetto, S. 2008. Neurosci
169	*PPIR3A*	Increased BW & adiposity	Knock-out	Delibegovic, M. 2003. Diabetes
170	*PPKAA2*	Increased adiposity	Knock-out	Villena, JA. 2004. Diabetes
171	*PREF1*	Obesity	Knock-out	Moon, YS. 2002. Mol Cell Biol
172	*PRKCQ*	Obesity	Transgenic	Serra, C. 2003. J Cell Physiol
173	*PRL*	Increased BW	Knock-out	Perez-Villamil, B. 1992. J Endocrinol
174	*PROX1*	Obesity in heterozygotes	Knock-out	Harvey, NL. 2005. Nat Genet
175	*PRRP*	Increased BW	Knock-out	Lawrence, C. 2002. Endocrinol
176	*PTPN11*	Obesity	Knock-out	Zhang, EE. 2004. Proc Natl Acad Sci USA
177	*PYY*	Obesity	Knock-out	Batterham, R. 2002. Nature
178	*RAGE*	Increased BW	Knock-out	Leuner, B. 2012. Z Gerentol Geriatr
179	*RAI1*	Obesity in heterozygotes	Knock-out	Bi, W. 2005. Hum Mol Genet
180	*REN*	Adult-onset obesity	Transgenic	Uehara, S. 2003. Int J Mol Med
181	*RETN*	Increased adiposity	Transgenic	Kim, KH. 2004. Proc Natl Acad Sci USA
182	*RPGRIP1L*	Obesity	Knock-out	Vadnais, C. 2013. BMC Genomics
183	*RSC1A1*	Obesity	Knock-out	Osswald, C. 2005. Mol Cell Biol
184	*RSL1*	Females prone to diet-induced obesity	Transgenic	Krebs, CJ. 2014. Mol Cell Biol
185	*SAR1B*	Obesity under HFD	Transgenic	Levy, E. 2014. J Nutr Biochem
186	*SDC1*	Adult-onset obesity	Transgenic	Reizes, O. 2001. Cell
187	*SELM*	Increased BW & adiposity	Transgenic	Pitts, MW. 2013. J Biol Chem
188	*SFRP1*	Increase in BW and adiposity under HFD	Knock-out	Gauger, KJ. 2013. PLoS One
189	*SH2B*	Obesity	Knock-out	Ren, D. 2005. Cell Metab
190	*SHP*	Obesity and increased adiposity	Transgenic	Tabbi-Anneni, I. 2010. Am J Physiol Endocrinol Metab
191	*SIM1*	Obesity in heterozygotes	Knock-out	Michaud, JL. 2001. Hum Mol Genet
192	*SIRT6*	Adult-onset obesity	Transgenic	Schwer, B. 2010. PNAS
193	*SLC2A4*	Increased adiposity	Transgenic	Carvalho, E. 2005. Am J Physiol Endocrinol Metab
194	*SLC6A1*	Obesity	Transgenic	Ma, YH. 2000. Cell Res
195	*SOCS1*	Liver degeneration, obesity	Knock-out	Starr, R. 1998. Proc Natl Acad Sci USA
196	*SOCS3*	Obesity under HFD	Knock-out	Sachithanandan, N. 2010. Hepatology
197	*SPARC*	Increased adiposity	Knock-out	Bradshaw, AD. 2003. Proc Natl Acad Sci USA
198	*SPONDIN 2*	Obesity	Knock-out	Zhu, LH. 2014. J Hepatol
199	*SRC-1*	Obesity	Knock-out	Picard, F. 2002. Cell
200	*STAT3*	Obesity	Cre/LoxP	Cui, Y. 2004. Mol Cell Biol
201	*STAT5B*	Increased adiposity	Knock-out	Gao, Q. 2004. Proc Natl Acad Sci USA
202	*TAp63*	Obesity	Knock-out	Su, X. 2012. Cell Metab
203	*T-BET*	Obesity	Knock-out	Kim, K. 2013. J Nutr Biochem
204	*THRA*	Increased BW & adiposity	Knock-out	Udy, GB. 1997. Proc Natl Acad Sci USA
205	*TIMP-2*	Obesity and hyperphagia	Knock-out	Stradecki, HM. 2011. J Neuroendocrinol
206	*TIS7*	Increased BW & adiposity	Transgenic	Wang, Y. 2005. J Biol Chem
207	*TNF*	Increased BW & adiposity	Transgenic	Liu, YY. 2003. J Biol Chem
208	*TNF-α*	Increased BW & adiposity	Knock-out	Salles, J. 2012. J Nutr Biochem
209	*TRKβ*	Increased BW & adiposity	Knock-in	Byerly, MS. 2013. PLoS One
210	*TRPV4*	Increased BW & adiposity	Knock-out	O’Conor, J. 2013. Ann Rheum Dis
211	*TUB*	Adult-onset obesity	Knock-out	Voros, G. 2004. J Thromb Haemost
212	*TW*	Obesity in heterozygotes	Knock-out	Kurima, K. 2011. PLoS Genet
213	*TXNIP*	Increased fat to muscle ratio	Knock-out	Stubdal, H. 2000. Mol Cell Biol
214	*UCP1*	Late-onset obesity with HFD	Knock-out	Kontani, Y. 2005. Aging Cell
215	*VDR*	Obesity	Transgenic	Wong, KE. 2011. J Biol Chem
216	*WDTC1*	Obesity in heterozygotes	Knock-out	Hader, T. 2003. EMBO
217	*XOR*	Increased BW & adiposity	Knock-out	Murakami, N. 2014. Arterioscler Thromb Vasc Biol
218	*ZEB1*	Obesity	Knock-out	Saykally, JN. 2009. PLoS One
219	*ZFP90*	Obesity	Transgenic	Schadt, EE. 2005. Nat Gen
220	*ZNT7*	Obesity in males only	Knock-out	Huang, L. 2012. J Biol Chem
221	*ZNT8*	Obesity under HFD	Knock-out	Lemaire. K, 2009. Proc Natl Acad Sci USA

**Notes.**

* BW, Body weight; HFD, High fat diet.

#### Leptin (LEP) and Leptin Receptor (LEPR)

The *obese* (*ob*) mutation was first described in 1949 by a team from Jackson Laboratory (Coleman & Hummel, 1973; Kanasaki & Koya, 2011). The *ob* mutation originated in non-inbred heterogeneous stock, but was subsequently transferred onto various inbred strains for further analysis (Clee & Attie, 2007; Coleman & Hummel, 1973; Coleman, 1982). This model exhibits morbid obesity associated with hyperphagia and hyperglycemia along with other neuroendocrine abnormalities (Coleman & Hummel, 1973; Coleman, 1982). Elegant parabiosis studies demonstrated that the *obese* gene encodes a circulating factor, while the *diabetes* gene encodes its receptor (Coleman, 1973). Through ground-breaking positional cloning studies, the *ob* mutation was characterized as a single-base deletion which results in a premature stop codon in the previously unknown leptin gene (Zhang et al., 1994). This landmark study was the first to identify this hormone and largely initiated research efforts on adipokines.

The *diabetes* (*db*) mutation was identified in 1966 in the C57BL/KsJ inbred mouse strain (Coleman & Hummel, 1967; Hummel, Dickie & Coleman, 1966). This mouse model exhibits persistent hyperphagia and obesity, resulting in hyperleptinemia, insulin resistance and increased leptin levels (Coleman & Hummel, 1967). Positional cloning and related studies identified the *diabetes* (*db*) mutation in the leptin receptor gene (Chen et al., 1996; Tartaglia et al., 1995).

After the discovery of leptin, a mutation in the leptin gene (*LEP*) was discovered in two severely obese cousins within a highly consanguineous family of Pakistani origin (Montague et al., 1997). The mutation was characterized as a frameshift mutation resulting in truncated transcription of leptin (Montague et al., 1997). Other reports have confirmed this initial discovery in additional homozygous patients of Pakistani, Turkish and Egyptian origin (Gibson et al., 2004; Mazen et al., 2009). In a study in Pakistan, where consanguineous marriages are preferred, 16.1% of the probands from 62 unrelated children with early onset obesity exhibited mutations in *LEP*. Of these probands, 9 carried a homozygous frameshift mutation. Given the high prevalence of monogenic obesity in this consanguineous population, early detection of this mutation for counselling and management of obesity could be beneficial (Saeed et al., 2012).

Similarly, shortly after its identification in mice, congenital leptin receptor (LEPR) deficiencies were found in severe obese siblings in 1998 (Clément et al., 1998). In a more recent study, 8 other patients with severe early onset obesity with homozygous or compound heterozygous mutations in *LEPR* were identified (Farooqi et al., 2007a; Farooqi et al., 2007b; Farooqi et al., 2007c). These patients exhibited high serum levels of leptin and loss of sensitivity of the receptor (Farooqi et al., 2007a; Farooqi et al., 2007b; Farooqi et al., 2007c).

Patients with mutations in *LEP* or *LEPR* experience rapid weight gain within the first year of life (Farooqi et al., 2002). Patients all experience hyperphagia and display aggression when food is denied (Farooqi et al., 2002). Onset of puberty is often delayed for these patients, due to hypogonadotrophic hypogonadism (Farooqi et al., 2007a; Farooqi et al., 2007b; Farooqi et al., 2007c). Leptin deficient children exhibit defective T-cell mediated immunity, explaining the high rates of infection and mortality in developing countries (Farooqi et al., 2007a; Farooqi et al., 2007b; Farooqi et al., 2007c).

The role of leptin in energy homeostasis has also been demonstrated in studies employing novel tools for genetic studies, such as whole exome sequencing. For example, whole exome sequencing was conducted in extreme obese individuals from four consanguineous families to determine the role of rare coding variants in pathogenesis of obesity (Gill et al., 2014). The study found two novel frameshift mutations (p.C186AfsX27 and p.H160LfsX9) that truncate the LEPR protein, resulting in protein products that lack the necessary binding domain for leptin signaling (Gill et al., 2014).

Considering the symptoms associated with leptin deficiency, the impact of leptin deficiency in the body is reversible *via* leptin treatment. A 9-year-old girl with leptin deficiency experienced reduction in weight mainly due to loss of fat, reduced energy intake, and increase in gonadotropin concentrations after treatment with recombinant human leptin for 12 months (Farooqi et al., 1999). In a different study, leptin-deficient patients in a fed state gave higher ratings to food images, but these ratings were reduced after leptin treatment (Farooqi et al., 2007a). As a result of these findings, leptin treatment has been deemed a promising therapeutic option for leptin deficient patients. It should be noted however that normal leptin levels do not preclude the presence of a deleterious mutation. A recent study described a 2-year-old boy with a deleterious leptin mutation with normal leptin levels (Wabitsch et al., 2015). This mutation had an impact on the protein function rather than expression, which questions the reliability of leptin levels as a prescreening tool for detecting leptin mutation.

#### TUB

Tubby bipartite transcription factor (TUB) is a member of the tubby-like proteins, which present a highly conserved C-terminus domain (Carroll, Gomez & Shapiro, 2004). *TUB* is a substrate for insulin receptor tyrosine kinase (IRTK) and leptin receptor Janus kinase 2 (LEPR JAK2) in the hypothalamus. TUB is translocated to the nucleus after binding to LEPR *via* JAK2. Inhibition of TUB expression in the hypothalamus results in increased food intake, fasting glucose levels, hepatic glucose output, decreased oxygen consumption, and reduced sensitivity of POMC to leptin (Prada et al., 2013). A mutation in the *tubby* gene occurred spontaneously at the Jackson Laboratory in a C57BL/6J mouse (Coleman & Eicher, 1990). These mice developed milder obesity compared to the other mutant models, hyperinsulinemia and mild hypoglycemia (Coleman & Eicher, 1990). Positional cloning of the mutated *tubby* gene identified a single-base change in the splice donor site that results in the incorrect retention of a single intron in the mature tub mRNA transcript (Kleyn et al., 1996).

Mutations in *TUB* were observed in an eleven-year-old boy from a consanguineous Caucasian family. His symptoms included deteriorating vision, obesity, and normal glucose/cholesterol/triglycerides levels but other clinical features were not observed to classify the patient as Bardet-Biedl or Alström syndrome (Borman et al., 2014). The mutation was identified as a homozygous frameshift mutation in *TUB* (c.1194_1195delAG, p.Arg398Serfs*9), which results in a truncated form of *TUB*. Homozygous loss of function of *TUB* is extremely rare in humans (Borman et al., 2014).

#### MC4R

The melanocortin-4 receptor (*Mc4r)* model was identified in 1997 through targeted gene disruption (Huszar et al., 1997). *MC4R* is a G protein couple receptor mainly expressed in the brain that is involved in both energy intake and expenditure (Gantz et al., 1993; Huszar et al., 1997). *Mc4r*^−/−^ mice exhibit obesity, hyperphagia, hyperinsulinemia, hyperglycemia, and increased linear growth (Huszar et al., 1997). Comparatively, *Mc4r*^+/−^ mice display milder forms of obesity, with increased weight gain in response to high-fat diet, suggesting a gene-dosage effect (Srisai et al., 2011).

The first heterozygous mutation in *MC4R* discovered in humans was in 1998 (Vaisse et al., 1998; Yeo et al., 1998). *MC4R* mutations represent the most common form of human monogenic obesity, impacting 0.2–5.6% of individuals with severe early onset obesity (Rouskas et al., 2012). Majority of these mutations are heterozygous, with homozygous mutants having a fully penetrant early-onset severe form of obesity. Not all heterozygote mutants are obese however, which is indicative of the dosage effect described previously in the mouse models (Farooqi et al., 2003; Stutzmann et al., 2008). In addition to obesity, *MC4R* deficient children display hyperinsulinemia and increased linear growth (Farooqi et al., 2000). Also patients experience an increase in adiposity, as well as an increase in lean mass, which is a phenotype that is not observed in other forms of monogenic obesity (Farooqi et al., 2003). Interestingly, the degree of hyperphagia in patients depends on level of receptor dysfunction, which is generally lower than that of leptin deficient patients (Farooqi et al., 2003).

#### MC3R

Both melanocortin receptor 3 (MC3R) and MC4R are expressed in the hypothalamus and are involved in energy homeostasis (Roselli-Rehfuss et al., 1993). *Mc3r* deficient mice exhibit 50–60% more adipose mass and 50% reduction in energy expenditure (Butler et al., 2000). *Mc3r* deficient mice are also hyperleptinaemic and male *Mc3r*^−/−^ mice develop mild hyperinsulinemia (Chen et al., 2000). Mice lacking both *Mc3r* and *Mc4r* become significantly heavier than either mutation alone, suggesting that *Mc3r* and *Mc4r* have non-redundant roles in energy homeostasis (Chen et al., 2000).

While the role of *MC4R* in monogenic obesity is well-defined, the role of *MC3R* mutations in human monogenic obesity is debated (Zegers, Beckers & Hendrickx, 2013). Although mutations in the *MC3R* gene may not be involved in autosomal dominant form of monogenic obesity, these mutations could predispose humans to increased risk of obesity. *MC3R* mutations that result in defective receptors have been associated with obesity in French and Italian populations (Mencarelli et al., 2011). A non-significant two-fold enrichment in *MC3R* loss-of-function mutations was observed in a severe obese population from United States (Calton et al., 2009).

#### POMC

**Figure 2:**
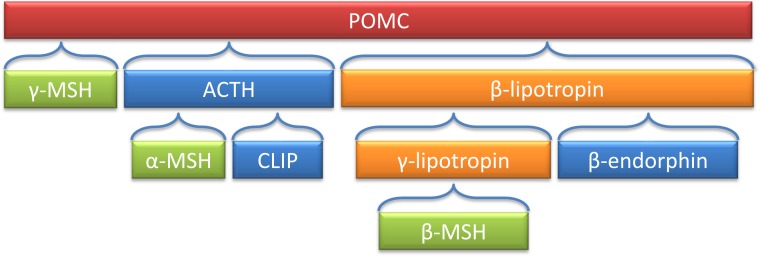
Processing of the POMC precursor protein. Adrenocorticotropic hormone (ACTH) and *β*-lipotropin are products generated in the corticotrophic cells of the anterior pituitary under the control of corticotropin releasing hormone (CRH). Alpha-melanocyte stimulating hormone (*α*-MSH), corticotropin-like intermediate lobe peptide (CLIP), *γ*-lipotropin and *β*-endorphin are products generated in the intermediate lobe of the pituitary under the control of dopamine. *α*-, *β*- and *γ*-MSH are collectively referred to as melanotropin or intermedin.

The pro-opiomelanocortin (*Pomc*) derived peptides have a variety of biological functions, such as pigmentation, adrenocortical function, and energy stores (Smith & Funder, 1988). Figure 2 is a depiction of *POMC*-derived peptides, including *α* and *β*-MSH. Deleting the coding region of *Pomc* in mouse models resulted in obesity, defective adrenal development and altered pigmentation (Yaswen et al., 1999).

Interest in the melanocortin pathway stemmed from the studies of agouti mice. Mouse coat color was a trait studied by the mouse model experts whose stocks founded many of the commonly studied strains (Clee & Attie, 2007). The dominant lethal “yellow” mutation (*A^y^*) was identified in 1905 (Dickies, 1962). A non-lethal, viable, allele (*A^vy^*) occurred as a spontaneous mutation in 1960 in the C3H/HeJ strain at the Jackson Laboratory (Dickies, 1962). In addition to their yellow coat color, mutant mice exhibit adult-onset obesity, type 2 diabetes associated with insulin resistance, hyperleptinemia, higher benign tumor susceptibility and infertility (Klebig et al., 1995). The mutations in the *agouti* gene in carriers of the yellow alleles leads to dysregulation of its expression in multiple tissues (Bultman, Michaud & Woychik, 1992; Duhl et al., 1994; Miller et al., 1993). The agouti model displays a defect in proopiomelanocortin (*POMC*) signaling pathway and is desensitized to leptin signaling (Boston et al., 1997).

The first recessive mutation in *POMC* was discovered in 1998 (Krude et al., 1998). In addition to obesity, patients with *POMC* mutations displayed hypocortisolism, hair and skin hypopigmentation, neonatal hypoglycemia, seizures, cholestasis and voracious appetite (Farooqi et al., 2006; Krude et al., 1998; Krude et al., 2003).The hypopigmentation of the hair and skin is not always observed in non-European populations, and *POMC* mutations should still be considered in individuals with severe early onset obesity even if typical pigmentary phenotype is missing (Cirillo et al., 2012; Clément et al., 2008; Mendiratta et al., 2011). In general, *POMC* deficiencies are extremely rare in human population (Beales, 2010) and the position of the mutation is important, as missense mutations have been reported to directly impact the melanocortin peptide-encoding regions, whereas other missense mutations have been reported to impact the peptide-receptor binding affinity (Challis et al., 2002).

A novel mutation in the alpha-melanocyte stimulating hormone (*α*-*MSH*) gene was found in a 12-year-old girl with early onset obesity (transmitted through the father) (Dubern et al., 2008). The mutation was characterized by dramatic impairment of *α*-*MSH* (Dubern et al., 2008). The patient’s obese father had less pronounced form of obesity in comparison to the daughter, which may be due to a gene-environment interaction. This means the younger generation is more exposed to obesogenic environment, thus more likely to develop obesity (Dubern et al., 2008). Most research has been focused on *α*-*MSH* since rodent models lack beta-melanocyte stimulating hormone (*β*-*MSH)* (Bennett, 1986) but a loss of function missense mutation in *β*-*MSH* has been associated with childhood obesity. The lack of function of *β*-*MSH* reduces the amount of MSH peptide in the POMC/MC4R pathway, resulting in obesity (Biebermann et al., 2006). *β*-*MSH* mutations may result in a non fully penetrant form of monogenic obesity, as some patients with this mutation are not obese (Lee et al., 2006).

#### PCSK1

A mutation was discovered in 1973 in the HRS/J inbred mouse strain which homozygous mice exhibit a slower increase in body weight compared to *ob/ob* and *db/db* mice but ultimately develop severe obesity, with hyperinsulinemia and transient hyperglycemia in males (Coleman & Eicher, 1990). Coding non-synonymous mutation in the carboxypeptidase E (*Cpe*) gene was found to induce the *fat* mouse phenotype (Naggert et al., 1995). *Cpe* gene is involved in processing of prohormone convertase 1 (*PC1)* as illustrated in Fig. 1, which led scientists to study the association of this gene to obesity as well (Li et al., 2014).

PC1/3 functions as a processing enzyme of precursor proteins in the regulated secretory pathways (Creemers, Jackson & Hutton, 1998). *Pc1/3* knock-out mice do not exhibit obesity, but instead show growth retardation and multiple neuroendocrine disorders (Zhu et al., 2002). An ENU mutagenesis experiment resulted in development of a mouse model with mutation on the *Pc1/3* (N222D allele) that exhibits obesity (Lloyd, Bohan & Gekakis, 2006). *Pc1*^*N*222*D*/*N*222*D*^ mice have lower *α*-*MSH* and display defects in *POMC* processing, affecting the melanocortin signaling (Lloyd, Bohan & Gekakis, 2006). *Pc1*^*N*222*D*/*N*222*D*^ mice exhibit abnormal proinsulin processing, multiple endocrinological defects, hyperphagia and obesity, while heterozygous mice exhibit an intermediate phenotype for weight gain and fasting insulin processing (Lloyd, Bohan & Gekakis, 2006).

In human studies, three patients with recessive monogenic form of obesity were deficient in the pro-protein convertase subtilisin/kexin type 1 (*PCSK1)* gene (Farooqi et al., 2007b; Jackson et al., 2003; Jackson et al., 1997). Complete prohormone convertase 1 deficiency results in early on-set severe obesity, hyperphagia, hypoglycemia, and endocrine dysfunction (Farooqi et al., 2007a; Farooqi et al., 2007b; Farooqi et al., 2007c; Jackson et al., 2003; Jackson et al., 1997). Null mutations causing prohormone convertase 1 congenital deficiency also lead to generalized malabsorptive diarrhea and diabetes insipidus in some instances (Farooqi et al., 2007a; Farooqi et al., 2007b; Farooqi et al., 2007c; Frank et al., 2013; Jackson et al., 1997; Martín et al., 2013; Yourshaw et al., 2013). Partial loss of function heterozygous mutations in *PCSK1* present a non-fully penetrant intermediate obesity phenotype (Creemers et al., 2012) However, heterozygous carriers of a null mutation show a dominantly inherited form of Mendelian obesity (Philippe et al., 2014).

#### SH2B1

The SH2B adaptor protein 1 (SH2B1) activates the JAK2 cytoplasmic tyrosine kinase to mediate cell signaling (Ren et al., 2005). SH2-B is a key regulator of leptin sensitivity. *Sh2b1*^−/−^ mice exhibit hyperphagia, hyperlipidemia, hyperglycemia, hyperleptinemia, hyperinsulinemia and hepatic steatosis (Ren et al., 2005).

In humans, loss of function mutations in *SH2B1* patients resulted in severe early onset obesity (Doche et al., 2012; Pearce et al., 2014). These patients exhibit hyperphagia, childhood onset obesity, insulin resistance, and reduced height (Doche et al., 2012). Behavioral abnormalities were also noted in patients, such as social isolation and aggression (Doche et al., 2012). The severity of the phenotype may depend on the impact of mutations on the disruption of different isoforms of SH2B1 (Pearce et al., 2014). Genomic imbalances and recurrent deletions of the *SH2B1* containing region on the short arm of chromosome 16 have been associated with behavioral disorders and obesity (Bachmann-Gagescu et al., 2010). It is interesting to note that while deletion of a region on chromosome 16 that contains *SH2B1* increases the risk of obesity significantly (Bochukova et al., 2010; Walters et al., 2010), reciprocal duplication of this region results in an increase in gene dosage which influences BMI in the reverse manner (leanness) (Jacquemont et al., 2011). The relevance of *SH2B1* locus in human energy balance is strengthened by the identification *via* GWAS of common variants near *SH2B1* associated with BMI variation or obesity risk (Berndt et al., 2013; Willer et al., 2009).

#### BDNF/NTRK2

The brain derived neurotrophic factor (BDNF) model demonstrates the numerous roles of *BDNF* in neural development through activation of TrkB and p75 receptors and involvement in anorexigenic activity (Noble et al., 2011). In the mature central nervous system, *BDNF* is expressed in various hypothalamic nuclei associated with eating behavior and obesity (Kernie, Liebl & Parada, 2000). To circumvent the problem of early mortality associated with total knock-out of the *BDNF* gene, conditional *BDNF* knockout mice were developed. Conditional knockout of *BDNF* in the brain *via* cre-loxP recombinase system resulted in mice exhibiting hyperphagia, hyperactivity and aggression as well as elevated levels of *POMC* (Rios et al., 2001). Since *BDNF* is only absent in the brain, the resulting obesity can be attributed to the lack of *BDNF* function therein (Rios et al., 2001). In another conditional knockout study, selective knockout of *BDNF* in brains of adult mice resulted in impaired hippocampal function, whereas selective knockout of *BDNF* in earlier stages of development resulted in more drastic phenotypes, such as hyperactivity and severe impairments in hippocampal-dependent learning (Monteggia et al., 2004).

Neurotrophin receptor (TrkB) is a member of the neurotrophin family and is known to be involved in development, maintenance and function of peripheral and central neurons and is hypothesized to play a role in mediating neuronal plasticity in the hypothalamus (Gray et al., 2006a). TrkB and its ligand BDNF are also known to be involved in the regulation of food intake and body weight (Gray et al., 2006a; Xu et al., 2003). Homozygous mutations in the gene encoding *TrkB* (*Ntrk2*) are lethal in mice, but heterozygous mutations resulting in 25% of *TrkB* expression display hyperphagia, increased linear growth and obesity as well as complex neurobehavioral phenotypes (Xu et al., 2003).

The neurotrophic tyrosine kinase receptor type 2 (*NTRK2)* gene was screened in a boy with early onset obesity, hyperphagia developmental delay, impairments in short-term memory and impaired nociception, revealing a missense mutation in *NTRK2* (Yeo et al., 2004). Further analysis showed an impairment in *BDNF*-stimulated protein kinase phosphorylation (Yeo et al., 2004). The developmental and neurological impairments in this case is consistent with the wide spread of *TrkB* (encoded *via NTRK2*) throughout the central nervous system, where it assumes the responsibility for neuronal survival and differentiation and regulation of synaptic function (Indo et al., 1996). In another case, a girl with loss of one functional copy of *BDNF* presented with hyperphagia, severe obesity, cognitive impairment and hyperactivity (Gray et al., 2006b). Moreover, hyperphagia and obesity observed in a subgroup of patients with WAGR syndrome has been attributed to deletions on chromosome 11 that induce haploinsufficiency of BDNF (Han et al., 2008).

#### SIM1

Single-minded homolog 1 (SIM1) is a member of the helix-hoop-helix PAS family of nuclear transcription factors (Crews, 1998). Homozygous *Sim1* mice die perinatally (Michaud et al., 1998), but heterozygous mutants exhibit hyperphagic obesity, increased body fat percentage (Holder et al., 2004; Michaud et al., 2001), as well as higher levels of *POMC* expression and resistance to *α*-*MSH* (Kublaoui et al., 2006). They are also more prone to diet-induced obesity (Holder et al., 2004) and are associated with defects in the *MC4R* signaling pathway (Kublaoui et al., 2006). To illustrate these signaling defects, *Sim1* heterozygous mouse injected with a melanocortin agonist showed a blunted suppression of food intake, while wild-type mice exhibited a robust reduction in food intake (Kublaoui et al., 2006).

Severe early-onset obesity was observed in a girl with haploinsufficiency of *SIM1*, possibly acting upstream or downstream of MC4R (Holder, Butte & Zinn, 2000). Further support for the involvement of *SIM1* in obesity came from studies in which patients displayed Prader-Willie like phenotypes due to heterozygous mutations in *SIM1* (Bonnefond et al., 2013). In another study, heterozygous mutations in *SIM1* were associated with severe obesity accompanied by a neurobehavioral phenotype for a majority of them (Ramachandrappa et al., 2013). Deletions on chromosome 6q16, including *SIM1* region, has been similarly associated with obesity and Prader-Willi like phenotype (Bonaglia et al., 2008). *SIM1* is expressed in kidneys and central nervous system and plays an essential role in formation of PVN of the hypothalamus (Michaud et al., 2000). This could be a mechanism in which *SIM1* plays a role in energy homeostasis, as PVN neurons express *MC4R* which inhibits food intake (Harris et al., 2001).

### Polygenic obesity mouse models and candidate gene studies in human

Given the success in identifying mutations causing severe monogenic obesity from mouse models, in parallel with the development of methods for linkage analysis, other mouse models have been developed for genetic studies of polygenic obesity. For example, the New Zealand Obese Mouse (NZO) characterizes a combination of hyperphagia, reduced energy expenditure and insufficient physical activity (Herberg & Coleman, 1977). The Kuo Kondo Mouse displays hyperphagia, hyperinsulinemia, insulin resistance (Igel et al., 1998) which precedes onset of obesity (Ikeda, 1994). Later modifications of this model led to development of KKA^*y*^ from transferring the *A^y^* gene, which is now used for obesity and diabetes research and testing of experimental therapies (Okazaki et al., 2002). The Tsumura Suzuki Obese Diabetes Mouse (TSOD) models polygenic obesity with diabetes (hyperglycemia and hyperinsulinemia) (Suzuki et al., 1999). The M16 mouse was developed to characterize the phenotypic consequences of long-term selective breeding for rapid weight gain (Allan, Eisen & Pomp, 2004). The M16 is an outbred model of early onset polygenic obesity and is characterized by hyperphagia, hyperinsulinemia, and hyperleptinemia (Allan, Eisen & Pomp, 2004). Lastly, the BSB mouse models are a backcross progeny obtained by crossing C57BL/6J x *Mus spretus* F1 females with C57BL/6J males to model polygenic obesity (Fisler et al., 1993; Warden et al., 1993). BSB mice range from 1% to 50% body fat with an increase in both intra-abdominal and subcutaneous fat (Fisler et al., 1993). Obesity in BSB model is associated with hyperinsulinemia, hyperglycemia, and hyperlipidemia (Fisler et al., 1993).

Studies of polygenic mouse models have involved the analysis of numerous inbred strains using multiple experimental designs, and dozens of loci have been mapped across all mouse chromosomes (Pomp, 2007; Rankinen et al., 2006). These QTLs affect body weight, body fat, high fat diet-induced weight gain, the severity of obesity, and more specific traits such as food intake, energy expenditure and exercise habits (Fawcett et al., 2008). Only a few studies revealed QTLs in regions that had been previously identified in monogenic studies. For example, a study using QTL mapping in the BSB mouse model identified a locus that is very proximal to the *LEP* gene, which had been previously identified *via* positional cloning (Warden et al., 1995; Warden et al., 1993; Zhang et al., 1994). To identify the causative variation, each locus identified in a chromosomal region is isolated in a congenic strain, essentially converting it a monogenic study where interactions with other loci are held constant. This facilitates the analysis of the locus under study.

However, positional cloning of genes underlying obesity QTLs has proven to be a difficult task with a limited success rate (Wuschke et al., 2007). Several factors have contributed to this, including the time and cost required to generate and phenotype sufficient congenic and sub-congenic strains to localize the QTL to a region where a single candidate can be identified. Another challenge has been that many of the QTLs that were originally mapped appear to have resulted from the combined effects of multiple nearby QTLs (Buchner et al., 2012; Laplante et al., 2012; Mollah & Ishikawa, 2011; Prevorsek et al., 2010; Shao et al., 2008; Yazbek et al., 2011). Thus, when isolating the loci in progressively smaller congenic strains, the individual effect sizes (i.e., the phenotypic difference between congenic genotypes) can diminish and could even seemingly disappear if they are within a strain that also harbors a locus acting in the opposite direction. Similar to the case in human polygenic obesity, adiposity in mice seems largely controlled by multiple loci having modest effects. Finally, between any pair of strains, there are haplotype blocks where the strains have numerous genetic differences, both coding and non-coding, that could contribute to a QTL.

Despite the relatively low success rate of positional cloning in identifying polygenic obesity genes, few success stories of employing this approach in mouse models are described below.

#### Cntnap2 and Tag1

Pioneering the use of chromosome substitution strains for positional cloning in mice, the Nadeau laboratory has recently identified two genes associated with diet-induced obesity (Buchner et al., 2012). A mutation in the *Cntnap2* gene which is required for proper potassium channel localization at neuronal nodes of Ranvier was identified through congenic analysis. Depending on the genetic background of the mouse model under investigation, this mutation either protected or predisposed mice to diet-induced obesity (Buchner et al., 2012). Using a candidate gene approach based on this finding, the group also assessed its known interacting protein, *Tag1*, in knockout mice and found that this gene also affects obesity by protecting mice against diet-induced obesity (Buchner et al., 2012). These studies have provided further evidence linking neuronal function with the regulation of body weight. Copy number variation in *Cntnap2* has recently been identified in a child with syndromic obesity (Vuillaume et al., 2014).

#### Deptor

The *Fob3a* locus was identified in studies of the Fat and Lean strains generated by long-term selection for these traits (Stylianou et al., 2004). Recently, through congenic analysis, genetic variation in *Deptor* has been identified as a strong obesity candidate gene at the *Fob3a* locus (Laplante et al., 2012). This gene was previously known for its roles in mammalian target of rapamycin (mTor) signaling, but its role in obesity development was unknown. Through the subsequent generation of transgenic mice, *Deptor* overexpression was associated with increased adipogenesis (Laplante et al., 2012).

#### Other obesity candidate genes identified through congenic analysis

Studies of the *Nob3* QTL have led to the identification of a microdeletion that eliminates expression of *Ifi202b* (Vogel et al., 2012). The authors showed that this altered the expression of several genes including *11 β*-*Hsd1*. *11 β*-*Hsd1* encodes the cortisone reductase and is a relevant candidate gene for energy balance. Another gene recently identified from mouse positional cloning studies is *Bhlhe40*, which affects muscle fatty acid oxidation (Takeshita et al., 2012).

Mouse models have also been helpful in elucidating genes that play a role in polygenic obesity risk/protection in humans. For example, candidate gene studies of *MC4R* common genetic variants revealed that the gain-of-function mutation of the variants lower the risk of obesity (Geller et al., 2004; Stutzmann et al., 2007). Additionally, the study of *BDNF* as a candidate gene led to the association of a coding non-synonymous variant (Val66Met) with BMI variation in healthy adults (Gunstad et al., 2006), an association confirmed later on by hypothesis-free GWAS for BMI (Thorleifsson et al., 2009; Willer et al., 2009). Similarly, two non-synonymous variants on *PCSK1* were consistently associated with childhood and adult severe obesity in a study of 13,659 participants of European ancestry, making *PCSK1* a candidate gene for polygenic obesity (Benzinou et al., 2008).

## From Human Obesity to Mouse Models: The Back and Forth

Mouse models have been invaluable in dissecting the genetic origin of human monogenic and polygenic obesity. The reverse approach also holds true as gene discoveries in humans have pioneered new mouse models for obesity. This section will review how genuine genetic discoveries in humans using high throughput, agnostic approaches such as positional cloning, GWAS or WES have inspired new experiments in mice to investigate the function of the genes.

### Positional cloning

#### BBS

Bardet-Biedl Syndrome (BBS) is a rare recessive developmental disorder, and people with heterozygous mutations in *BBS* gene are more prone to obesity (Croft et al., 1995; Sheffield, 2010). Positional cloning, homozygosity mapping, candidate gene and whole exome sequencing approaches have led to the discovery of 18 genes that are linked to BBS so far (Scheidecker et al., 2014). Follow-up studies in animal models revealed the association of *BBS* genes with cilia function, and in intracellular and intraflagellar trafficking (Sheffield, 2010). More specifically, mice homozygous for a single *BBS* mutation lack spermatozoa flagella (Sheffield, 2010). Further analysis of BBS resulted in development of *BBS* knockout mice (*Bbs2, Bbs4* and *Bbs6*) that developed hyperphagia, reduced energy expenditure and increased circulating leptin levels, which suggests that leptin deficiency is not the mechanism of obesity in BBS (Rahmouni et al., 2008). This was further confirmed by administration of leptin, which failed to change body weight or food intake, indicative of leptin resistance in BBS mice (Rahmouni et al., 2008). On the other hand, expression of *Pomc* in *BBS* knock-out mice were significantly lower than that of controls, while *Agrp* and *Npy* levels were comparable to controls, pointing to the idea that *Pomc* is the main player in obesity in *BBS* mice (Rahmouni et al., 2008). This phenomenon is compatible with the role of *BBS* in cilia function, as abrogating cilia in POMC neurons increases food intake and causes obesity in mice (Davenport et al., 2007).

#### TBC1D1

A major predisposing locus for obesity was identified at 4p.15-14, affecting more females than males (Arya et al., 2004; Meyre et al., 2008; Stone et al., 2002). Positional cloning of this 4p15-14 linkage peak led to the identification of a coding non-synonymous variant (R125W) in the TBC1 domain family member 1 (*TBC1D1*) gene associated with female familial obesity in populations from Utah and France (Meyre et al., 2008; Stone et al., 2006). TBC1D1 is a Rab-GTPase activating protein and is closely related to insulin signaling protein AS160. It is predominantly expressed in skeletal muscle, as it is involved in regulation of lipid utilization in skeletal muscles (An et al., 2010; Chadt et al., 2008; Sano et al., 2003). These discoveries in humans were followed-up by animal work. Studies of the *Nob1* QTL identified a mutation in the *Tbc1d1* gene that protects against obesity (Chadt et al., 2008). *Tbc1d1* knockout mice were shown to exhibit reduction in body weight, impaired glucose utilization, and increased lipid oxidation in skeletal muscles (Dokas et al., 2013). Further analysis of the deleterious effects of the human R125W mutation has been confirmed by *in vivo* overexpression of wild-type and mutant *TBC1D1* proteins in mouse tibialis anterior muscles (An et al., 2010). In this study, the R125W mutation impaired insulin-stimulated glucose transport, but did not impair contraction-stimulated glucose transport. Experiments conducted on phosphorylation sites of other *TBC1D1* mutations had opposing effects, as these mutations impaired contraction-stimulated glucose transport but did not impact insulin-stimulated glucose transport (An et al., 2010). Overall, impairment of muscle glucose transport could lead to increased fat accumulation in adipose tissue and result in subsequent obesity (An et al., 2010).

#### ENPP1

A positional cloning experiment led to identification of ectonucleotide pyrophosphatase/phosphodiesterase 1 (*ENPP1*) as a possible contributor to obesity and type 2 diabetes in humans. A significant linkage for childhood obesity was detected on chromosome 6q22.23 in French pedigrees (Meyre et al., 2004). By using overlapping published linkage studies on obesity (Atwood et al., 2002), insulin secretion (Abney, Ober & McPeek, 2002; Duggirala et al., 2001) and type 2 diabetes (Demenais et al., 2003; Ehm et al., 2000; Ghosh et al., 2000; Xiang et al., 2004), the *ENPP1* gene was picked for further analysis. *ENPP1* directly inhibits insulin-induced conformational changes of the insulin receptor, and affects its activation and signaling (Maddux & Goldfin, 2000; Maddux et al., 1995). A risk haplotype of *ENPP1* was associated with childhood obesity, glucose intolerance and type 2 diabetes (Meyre et al., 2005). Subsequent development of *Enpp1* knockout mice highlighted a phenotype of more efficient adipocyte maturation in mesenchymal embryonal cells compared to wild-type (Liang et al., 2007). Transgenic mice overexpressing human *ENPP1* in muscle and liver tissue exhibited elevation in glucose and insulin levels compared to wild-type, conveying that *ENPP1* plays a role in insulin resistance and hyperglycemia (Dong et al., 2005; Maddux et al., 2006).

### Genome-wide association studies

GWAS in obesity field have identified 119 independent loci associated with BMI and obesity status (Choquet & Meyre, 2011; Locke et al., 2015). Table 2 provides a summary of these identified genes and associated SNPs. Interestingly, GWAS showed that almost all genes involved in Mendelian forms of obesity in mice and humans (*LEPR, POMC, MC4R, BDNF, PCSK1, TUB, NTRK2, SH2B1*) display common variants associated with BMI and polygenic obesity as well (Dickson et al., 2010). Genetic animal models were developed prior to GWAS discoveries for these genes whose functions were well-established. However, most of the genes located in or near GWAS signals were of unknown function. This prompted the scientific community to develop new genetic mouse models for some of these genes that we describe below.

**Table 2:** List of genes and SNPs associated with body mass index (BMI) or binary obesity from genome-wide association studies (GWAS).

Gene	SNPs	Chrom.	Phenotype	Reference
ADCY9	rs2531995	16	Obesity	Berndt, SI. 2013. Nat Genet
AGBL4	rs657452	1	BMI	Locke, AE. 2015. Nature
ASB4	rs6465468	7	BMI	Locke, AE. 2015. Nature
BDNF	rs6265, rs4923461,rs10767664, rs2030323, rs988712	11	BMI, Obesity, Overweight	Thorleifsson, G. 2009. Nat Genet, Speliotes, EK. 2010. Nat Genet, Jiao, H. 2011. BMC Med Genomics, Okada, Y. 2012. Nat Genet, Wen, W. 2012. Nat Genet 2012
BRE	rs116612809	2	BMI	Gong, J. 2013. Am J Hum Genet
C9orf93	rs4740619	9	BMI	Locke, AE. 2015. Nature
CADM1	rs12286929	11	BMI	Locke, AE. 2015. Nature
CADM2	rs13078807	3	BMI, Overweight	Speliotes, EK. 2010. Nat Genet, Berndt, SI. 2013. Nat Genet
CALCR	rs9641123	7	BMI	Locke, AE. 2015. Nature
CBLN1	rs2080454	16	BMI	Locke, AE. 2015. Nature
CDKAL1	rs2206734, rs9356744	6	BMI	Okada, Y. 2012. Nat Genet, Wen, W. 2012. Nat Genet 2012
CLIP1	rs11057405	12	BMI	Locke, AE. 2015. Nature
CREB1, KLF7	rs17203016	2	BMI	Locke, AE. 2015. Nature
EHBP1	rs11688816	2	BMI	Locke, AE. 2015. Nature
ELAVL4	rs11583200	1	BMI	Locke, AE. 2015. Nature
EPB41L4B, C9orf4	rs6477694	9	BMI	Locke, AE. 2015. Nature
ERBB4	rs7599312	2	BMI	Locke, AE. 2015. Nature
ETS2	rs2836754	21	BMI	Locke, AE. 2015. Nature
ETV5	rs7647305, rs9816226	3	BMI, Obesity, Overweight	Thorleifsson, G. 2009. Nat Genet, Speliotes, EK. 2010. Nat Genet, Berndt, SI. 2013. Nat Genet
FAIM2	rs7138803, rs7132908	12	BMI, Obesity, Overweight	Thorleifsson, G. 2009. Nat Genet, Speliotes, EK. 2010. Nat Genet, Paternoster, L. 2011. PLoS One, Bradfield, JP. 2012. Nat Genet, Berndt, SI. 2013. Nat Genet
FANCL	rs887912	2	BMI, Obesity, Overweight	Speliotes, EK. 2010. Nat Genet, Berndt, SI. 2013. Nat Genet
FANCL, FLJ30838	rs12617233	2	BMI	Guo, Y. 2012. Hum Mol Genet
FHIT	rs2365389	3	BMI	Locke, AE. 2015. Nature
FIGN	rs1460676	2	BMI	Locke, AE. 2015. Nature
FLJ35779	rs2112347	5	BMI, Obesity, Overweight	Speliotes, EK. 2010. Nat Genet, Berndt, SI. 2013. Nat Genet
FOXO3, HSS00296402	rs9400239	6	BMI	Locke, AE. 2015. Nature
FTO	rs9939609, rs9930506, rs1121980, rs1421085, rs8050136, rs1558902, rs17817449, rs12149832, rs9940128, rs62033400, rs1421085, rs1121980, rs9936385, rs9941349, rs3751812, rs1558902, rs17817449	16	BMI, Obesity, Childhood Obesity	Dina, C. 2007. Nat Genet, Hinney, A. PLoS One. 2007, Frayling, TM. 2007. Science, Scuteri, A. 2007. PLoS Genet, Loos, RJ. 2008. Nat Genet, Meyre, D. 2009. Nat Genet, Thorleifsson, G. 2009. Nat Genet, Willer, CJ. 2009. Nat Genet, Cho, YS. 2009. Nat Genet, Speliotes, EK. 2010. Nat Genet, Scherag, A. 2010. PLoS Genet, Paternoster, L. 2011. PLoS One, Wang, K. 2011. PLoS One, Bradfield, JP. 2012. Nat Genet, Wen, W. 2012. Nat Genet, Okada, Y. 2012. Nat Genet, Guo, Y. 2012. Hum Mol Genet, Graff, M. 2013. Hum Mol Genet, Pei, YF. 2013. Hum Mol Genet, Berndt, SI. 2013. Nat Genet, Wheeler, E. 2013. Nat Genet
GBE1	rs3849570	3	BMI	Locke, AE. 2015. Nature
GDF15, PGPEP1	rs17724992	19	BMI	Locke, AE. 2015. Nature
GIPR	rs2287019, rs11671664	19	BMI	Speliotes, EK. 2010. Nat Genet, Wen, W. 2012. Nat Genet, Okada, Y. 2012. Nat Genet
GNAT2	rs17024258	1	Obesity	Berndt, SI. 2013. Nat Genet
GNPDA2	rs10938397, rs13130484, rs348495	4	BMI, Obesity, Overweight	Willer, CJ. 2009. Nat Genet, Speliotes, EK. 2010. Nat Genet, Graff, M. 2013. Hum Mol Genet, Berndt, SI. 2013. Nat Genet
GP2	rs12597579	16	BMI	Wen, W. 2012. Nat Genet 2012
GPRC5BB	rs12444979	16	BMI, Obesity, Overweight	Speliotes, EK. 2010. Nat Genet, Berndt, SI. 2013. Nat Genet
GRID1	rs7899106	10	BMI	Locke, AE. 2015. Nature
GRP	rs7243357	18	BMI	Locke, AE. 2015. Nature
GRP120	rs116454156	10	Obesity	Ichimura, A. 2012. Nature
HHIP	rs11727676	4	BMI	Locke, AE. 2015. Nature
HIF1AN	rs17094222	10	BMI	Locke, AE. 2015. Nature
HIP1, PMS2L3, PMS2P5, WBSCR16	rs1167827	7	BMI	Locke, AE. 2015. Nature
HMGA1	rs206936	6	BMI	Speliotes, EK. 2010. Nat Genet
HNF4G	rs4735692	8	Obesity	Berndt, SI. 2013. Nat Genet
HOXB5	rs9299	17	Childhood Obesity	Bradfield, JP. 2012. Nat Genet
HS6ST3	rs7989336	13	Obesity	Berndt, SI. 2013. Nat Genet
HSD17B12	rs2176598	11	BMI	Locke, AE. 2015. Nature
IFNGR1, OLIG3	rs13201877	6	BMI	Locke, AE. 2015. Nature
KAT8, ZNF646, VKORC1, ZNF668, STX1B, FBXL19	rs9925964	16	BMI	Locke, AE. 2015. Nature
KCNK3	rs11126666	2	BMI	Locke, AE. 2015. Nature
KCNMA1	rs2116830	101	Obesity	Jiao, H. 2011. BMC Med Genomics
KCTD15	rs11084753, rs29941	19	BMI	Willer, CJ. 2009. Nat Genet, Thorleifsson, G. 2009. Nat Genet, Speliotes, EK. 2010. Nat Genet
KLF9	rs11142387	9	BMI	Okada, Y. 2012. Nat Genet
LEPR	rs11208659	1	Childhood Obesity	Wheeler, E. 2013. Nat Genet
LMX1B	rs10733682	9	BMI	Locke, AE. 2015. Nature
LOC100287559, BBS4	rs7164727	15	BMI	Locke, AE. 2015. Nature
LOC284260, RIT2	rs7239883	18	BMI	Locke, AE. 2015. Nature
LOC285762	rs9374842	6	BMI	Locke, AE. 2015. Nature
LPIN2	rs643507	18	Obesity (Asthmatic patients)	Melen, E. 2013. Clin Exp Allergy
LRP1B	rs2890652	2	BMI	Speliotes, EK. 2010. Nat Genet
LRRN6C	rs10968576	9	BMI, Obesity	Speliotes, EK. 2010. Nat Genet, Berndt, SI. 2013. Nat Genet
MAF	rs1424233	16	Obesity	Meyre, D. 2009. Nat Genet
MAP2K5	rs2241423, rs4776970, rs997295	15	BMI, Obesity, Overweight	Speliotes, EK. 2010. Nat Genet, Wen, W. 2012. Nat Genet 2012, Guo, Y. 2012. Hum Mol Genet, Berndt, SI. 2013. Nat Genet
MAPK3, KCTD13, INO80E, TAOK2, YPEL3, DOC2A, FAM57B	rs4787491	16	BMI	Locke, AE. 2015. Nature
MC4R	rs17782313, rs571312, rs12970134, rs2331841, rs6567160, rs8089364, rs7234864, rs723486, rs7227255, rs2229616, rs17782313, rs17700144,rs663129, rs571312, rs476828	18	BMI, Obesity, Overweight	Loos, RJ. 2008. Nat Genet, Thorleifsson, G. 2009. Nat Genet, Meyre, D. 2009. Nat Genet, Speliotes, EK. 2010. Nat Genet, Scherag, A. 2010. PLoS Gene, Paternoster, L. 2011. PLoS One, Guo, Y. 2012. Hum Mol Genet, Okada, Y. 2012. Nat Genet, Wen, W. 2012. Nat Genet, Bradfield, JP. 2012. Nat Genet, Berndt, SI. 2013. Nat Genet, Wheeler, E. 2013. Nat Genet, Graff, M. Hum Mol Genet. 2013, Pei, YF. Hum Mol Genet. 2013
MIR548A2	rs1441264	13	BMI	Locke, AE. 2015. Nature
MIR548X2, PCDH9	rs9540493	13	BMI	Locke, AE. 2015. Nature
MRPS33P4	rs13041126	20	Obesity	Berndt, SI. 2013. Nat Genet
MTCH2	rs10838738, rs3817334	11	BMI, Obesity, Overweight	Willer, CJ. 2009. Nat Genet, Speliotes, EK. 2010. Nat Genet, Berndt, SI. 2013. Nat Genet
MTIF3	rs4771122	13		Speliotes, EK. 2010. Nat Genet
NAV1	rs2820292	1	BMI	Locke, AE. 2015. Nature
NEGR1	rs2815752, rs2568958, rs1993709	1	BMI, Obesity, Overweight	Willer, CJ. 2009. Nat Genet, Thorleifsson, G. 2009. Nat Genet, Speliotes, EK. 2010. Nat Genet, Berndt, SI. 2013. Nat Genet, Wheeler, E. 2013. Nat Genet
NLRC3	rs758747	16	BMI	Locke, AE. 2015. Nature
NPC1	rs1805081	18	Obesity	Meyre, D. 2009. Nat Genet
NRXN3	rs10150332	14	BMI, Obesity	Speliotes, EK. 2010. Nat Genet, Berndt, SI. 2013. Nat Genet
NT5C2, CYP17A1, SFXN2	rs11191560	10	BMI	Locke, AE. 2015. Nature
NTRK2	rs1211166	9	BMI	Guo, Y. 2012. Hum Mol Genet
NUP54, SCARB2	rs17001654	4	BMI	Locke, AE. 2015. Nature
OLFM4	rs9568856, rs9568867	13	Obesity	Bradfield, JP. 2012. Nat Genet, Berndt, SI. 2013. Nat Genet
PACS1	rs564343	11	Childhood Obesity	Wheeler, E. 2013. Nat Genet
PARK2	rs13191362	6	BMI	Locke, AE. 2015. Nature
PCSK1	rs261967, rs6232, rs6234, rs6235	5	BMI, Obesity	Benzinou, M. 2008. Nat Genet, Wen, W. 2012. Nat Genet
PLCD4, CYP27A1, USP37, TTLL4, STK36, ZNF142,RQCD1	rs492400	2	BMI	Locke, AE. 2015. Nature
PMS2L11	rs2245368	7	BMI	Locke, AE. 2015. Nature
POMC	rs713586, rs6545814, rs1561288, rs6752378, rs10182181	2	BMI, Obesity, Overweight	Speliotes, EK. 2010. Nat Genet, Wen, W. 2012. Nat Genet 2012, Bradfield, JP. 2012. Nat Genet, Berndt, SI. 2013. Nat Genet, Graff, M. 2013. Hum Mol Genet
PRKCH	rs1957894	14	Childhood Obesity	Wheeler, E. 2013. Nat Genet
PRKD1	rs11847697, rs12885454	14	BMI	Speliotes, EK. 2010. Nat Genet, Locke, AE. 2015. Nature
PTBP2	rs1555543	1	BMI	Speliotes, EK. 2010. Nat Genet
QPCTL	rs2287019	19	Obesity, Overweight	Berndt, SI. 2013. Nat Genet
RABEP1	rs1000940	17	BMI	Locke, AE. 2015. Nature
RALYL	rs2033732	8	BMI	Locke, AE. 2015. Nature
RARB	rs6804842	3	BMI	Locke, AE. 2015. Nature
RASA2	rs16851483	3	BMI	Locke, AE. 2015. Nature
RMST	rs11109072	12	Childhood Obesity	Wheeler, E. 2013. Nat Genet
RPL27A	rs11042023	11	Obesity	Berndt, SI. 2013. Nat Genet
RPTOR	rs7503807	17	Overweight	Berndt, SI. 2013. Nat Genet
SBK1, APOBR	rs2650492	16	BMI	Locke, AE. 2015. Nature
SCG3, DMXL2	rs3736485	15	BMI	Locke, AE. 2015. Nature
SDCCAG8	rs12145833	1	Childhood Obesity	Scherag, A. 2010. PLoS Gene
SEC16B	rs10913469, rs543874, rs574367, rs516636, rs591120	1	BMI, Obesity, Overweight	Thorleifsson, G. 2009. Nat Genet, Speliotes, EK. 2010. Nat Genet, Bradfield, JP. 2012. Nat Genet, Berndt, SI. 2013. Nat Genet, Graff, M. 2013. Hum Mol Genet, Wen, W. 2012. Nat Genet, Okada,Y. 2012. Nat Genet
SH2B1	rs7498665, rs4788102, rs7359397, rs4788099	16	BMI, Obesity, Overweight	Willer, CJ. 2009. Nat Genet, Thorleifsson, G. 2009. Nat Genet, Speliotes, EK. 2010. Nat Genet, Guo, Y. 2012. Hum Mol Genet, Berndt, SI. 2013. Nat Genet
SLC39A8	rs13107325	4	BMI	Speliotes, EK. 2010. Nat Genet
SMG6, N29617	rs9914578	17	BMI	Locke, AE. 2015. Nature
STXBP6	rs10132280	14	BMI	Locke, AE. 2015. Nature
TAL1	rs977747	1	BMI	Locke, AE. 2015. Nature
TCF7L2	rs7903146	10	BMI	Locke, AE. 2015. Nature
TDRG1, LRFN2	rs2033529	6	BMI	Locke, AE. 2015. Nature
TFAP2B	rs987237, rs734597, rs2272903	6	BMI, Obesity, Overweight	Speliotes, EK. 2010. Nat Genet, Paternoster, L. 2011. PLoS One, Guo, Y. 2012. Hum Mol Genet, Berndt, SI. 2013. Nat Genet
TLR4	rs1928295	9	BMI	Locke, AE. 2015. Nature
TMEM160	rs3810291, rs3810291	19	BMI, Obesity	Speliotes, EK. 2010. Nat Genet, Berndt, SI. 2013. Nat Genet
TMEM18	rs6548238, rs7561317, rs2867125, rs12463617, rs4854344	2	BMI, Obesity, Overweight	Willer, CJ. 2009. Nat Genet, Thorleifsson, G. 2009. Nat Genet, Speliotes, EK. 2010. Nat Genet, Guo, Y. 2012. Hum Mol Genet, Bradfield, JP. 2012. Nat Genet, Berndt, SI. 2013. Nat Genet, Wheeler, E. 2013. Nat Genet, Graff, M. 2013. Hum Mol Genet
TNKS	rs17150703	8	Childhood Obesity	Scherag, A. 2010. PLoS Gene
TNNI3K	rs1514175, rs12142020, rs1040070, rs1514174	1	BMI, Obesity	Speliotes, EK. 2010. Nat Genet, Bradfield, JP. 2012. Nat Genet, Graff, M. 2013. Hum Mol Genet, Berndt, SI. 2013. Nat Genet
TOMM40, APOE, APOC1	rs2075650	19	BMI	Guo, Y. 2012. Hum Mol Genet
TUB	rs4929949	11	BMI	Speliotes, EK. 2010. Nat Genet
UBE2E3	rs1528435	2	BMI	Locke, AE. 2015. Nature
ZBTB10	rs16907751	8	BMI	Locke, AE. 2015. Nature
ZNF608	rs48361333	5	BMI	Speliotes, EK. 2010. Nat Genet
ZZZ3	rs17381664	1	Obesity	Berndt, SI. 2013. Nat Genet

#### FTO

The fat mass and obesity associated gene (*FTO*) is the first gene that has been convincingly associated with obesity using GWAS. The role of *FTO* as an important contributor to polygenic obesity was confirmed in GWAS for type 2 diabetes, BMI, early onset obesity, and incidentally in a population stratification approach (Dina et al., 2007; Frayling et al., 2007; Hinney et al., 2007; Scuteri et al., 2007). Initial GWAS on *FTO* identified *FTO* as an unknown gene in an unknown pathway (Frayling et al., 2007). The exact molecular mechanism of how *FTO* might contribute to obesity is still under investigation, but high level of expression of *FTO* in the hypothalamus is suggestive of its role in food intake (Fredriksson et al., 2008). This required further studies of *FTO* in knockout and overexpression animal models to understand the mechanism in which *FTO* influences obesity (Choquet & Meyre, 2011).

*Fto* knockout mice exhibit high perinatal lethality, significant reduction in body length, fat mass and lean mass, indicative of the role of *Fto* in energy homeostasis (Church et al., 2009; Fischer et al., 2009; McMurray et al., 2013). Deletion of *Fto* in the hypothalamus *via* adeno-associated viral vectors encoding Cre recombinase resulted in small reduction in food intake and decreased weight gain with no effect on energy expenditure (McMurray et al., 2013). Overexpression of Fto in mice results in increased food intake, increase in body and fat mass (Church et al., 2010).

Despite the metabolic phenotypes found in FTO overexpression/inactivation rodent models and the location of SNPs associated with human obesity in the intron 1 region of *FTO*, the implication of other neighboring genes in obesity is not excluded. As an illustration, the obesity-associated *FTO* sequence directly interacts with the promoters of *IRX3* as well as *FTO* in the human, mouse and zebrafish genomes (Smemo et al., 2014). Expression QTL studies in human brains revealed that obesity associated SNPs in *FTO* are associated with expression of *IRX3*, but not *FTO* itself (Smemo et al., 2014). *Irx3* knockout mice show reduction in body weight and increase in basal metabolic rate, indicative of a direct link between *Irx3* and body composition (Smemo et al., 2014). The rs8050136 SNP located in the first intron of *FTO* modulates the binding for CUX1 P110 and P200 isoforms which in turn regulate the expression of *FTO* and of the nearby ciliary gene *RPGRIPL1* (Stratigopoulos et al., 2014; Stratigopoulos et al., 2011). Homozygous mutants of *Rpgripl1* are lethal, but *Rpgrip1l* ^+/−^ mice are leptin resistant, hyperphagic and obese (Stratigopoulos et al., 2014). Overall, these animal experiments suggest that the genes *IRX3* and *RPGRIPL1* may mediate at least in part the association of SNPs in *FTO* with obesity.

#### NPC1

Besides *FTO*, genome wide association studies revealed that two non-synonymous variants in high linkage disequilibrium (H215R/I858V) in the Niemann-Pick C1 (*NPC1*) gene were associated with extreme obesity in European adults (Meyre et al., 2009). Subsequent mouse models of partial inactivation of *Npc1* evidenced significant weight gain in mice fed a high-fat diet, indicating a possible gene-diet interaction (Jelinek et al., 2010). More recently, SNPs at the *Npc1* locus have been associated with differences of body fat (%) in response to high-fat high-sucrose diet in a GWAS performed in mice (Parks et al., 2013).

#### ETV5

Ets variant 5 (ETV5) is a transcription factor that can act either as an activator or repressor of transcription of genes involved in cell proliferation, differentiation, apoptosis and cell–cell or cell–matrix interaction (Sementchenko & Watson, 2000; Sharrocks, 2001). Two GWAS in populations of European ancestry identified SNPs near *ETV5* as associated with BMI and obesity (Berndt et al., 2013; Speliotes et al., 2010; Thorleifsson et al., 2009). This association triggered further research on *ETV5* in animal models. *Etv5* knockout mice exhibit lean bodies, resistance to diet-induced obesity and severe glucose intolerance due to impaired insulin exocytosis and hypoinsulinaemia (Gutierrez-Aguilar et al., 2014).

#### NEGR1

GWAS identified SNPs near the neuronal growth regulator 1 (*NEGR1*) gene associated with BMI variation and obesity (Berndt et al., 2013; Thorleifsson et al., 2009; Wheeler et al., 2013; Willer et al., 2009). *NEGR1* is expressed in the brain and participates in the neurite outgrowth in the developing brain (Marg et al., 1999). With the use of ENU mutagenesis, mice carrying a loss of function of *Negr1* displayed reduced food intake and physical activity, unchanged energy expenditure and reduction in overall body mass (Lee et al., 2012).

### Next generation sequencing (NGS)

#### SDCCAG8

Nephronophthisis-related ciliopathies (NPHP-RCs) are developmental problems that impact kidneys and are associated with renal degeneration, intellectual disability and obesity. Exome sequencing identified 12 truncating mutations on serologically defined colon cancer antigen 8 (*SDCCAG8)* gene, showing that a loss of function of *SDCCAG8* is causal for human retinal-renal ciliopathy (Otto et al., 2010). The candidacy of *SDCCAG8* gene was strengthened by the identification of common variants associated with childhood obesity through a GWAS in German and French populations (Scherag et al., 2010). To better understand the function of *SDCCAG8*, a gene-trap mouse line (*Sdccag8*^*gt*/*gt*^) was subsequently developed (Airik et al., 2014). The *Sdccag8*^*gt*/*gt*^ mice exhibited the human phenotype of NPHP-RCs and revealed that retinal degeneration associated with the disorder exhibits early and leads to progressive loss of vision, whereas the renal degeneration occur later due to DNA damage from signaling activity (Airik et al., 2014).

## The Waltz Between Mouse and Human Genetic Studies

Recent attempts in understanding genetics of obesity utilizing both human and animal genetic approaches are discussed below.

### Linkage study

Arrestin domain-containing 3 protein (*ARRDC3*) is a regulator of cell receptor signaling, and also plays a role in metabolism (Luan et al., 2009). Genome wide linkage for human obesity identified a linkage peak on chromosome 5, and positional cloning identified *ARRDC3* associated with higher BMI in males but not in females (Patwari et al., 2011). Higher *ARRDC3* expression is associated with visceral adipose tissue and obesity in males. Animal models such as the *Arrdc3* deficient mice have validated the role of *ARRDC3* in metabolism by being resistant to obesity in a dosage dependent manner (both genders, but with greater impact on males than females) (Patwari et al., 2011).

### Candidate gene approach

G-protein coupled receptor 120 (GPR120) is a receptor for unsaturated long chain fatty acids, and plays a role in adipogenesis, regulation of appetite and food preference (Hirasawa et al., 2005). *Gpr120* deficient mice fed a high fat diet exhibit obesity, glucose intolerance, fatty liver, decreased adipocyte differentiation and lipogenesis (Ichimura et al., 2012), but no difference in body weight between *Gpr120* deficient and wild type mice was observed when both groups were fed a normal diet (Ichimura et al., 2012). When assessed in humans, *GPR120* was expressed in adipose tissue, with obese individuals having a higher expression in both subcutaneous and omental adipose tissue (1.8 fold increase) (Ichimura et al., 2012). In order to study the contribution of *GPR120* to human obesity, the four *GPR120* exons were sequenced in 312 non-consanguineous extremely obese French children and adults. Exon sequencing revealed a deleterious non-synonymous variant (p.R270H) of minor allele frequency (MAF) of 3% that inhibits *GPR120* signaling activity and increases the risk of obesity by 62% in 6,942 obese individuals and 7,654 control subjects from Europe (Ichimura et al., 2012). Thus, *GPR120* plays a role in sensing dietary fat, and is important in energy balance.

Melanocortin 2 receptor accessory protein 2 (MRAP2) is a homologue of MRAP, expressed in the brain and adrenal gland (Chan et al., 2009). *MRAP2* can interact with all melanocortin receptors, which results in *MC2R* surface expression and signaling. *MRAP2* can also reduce the responsiveness of *MC1R, MC3R, MC4R* and *MC5R* to *α*-*MSH* (Chan et al., 2009). Mouse models deficient in *Mrap2* exhibit obesity (Asai et al., 2013). Selective knockout of *Mrap2* in neurons expressing *Sim1* also exhibit obesity, similar to global knockout of *Mrap2*, consistent with the idea that *Sim1* expressing neurons are key regulators of energy balance (Asai et al., 2013). Four rare heterozygous mutations of *MRAP2* have been identified in obese humans (Asai et al., 2013).

#### Whole exome sequencing

Kinase suppressor of Ras 2 (KSR2) is a scaffolding protein involved in multiple signaling pathways through kinase cascades (Dougherty et al., 2010; Pearce et al., 2013) that are linked to regulation of food intake, body fat content and glucose homeostasis (Revelli et al., 2011). By using a whole-exome sequencing strategy, *KSR2* loss-of-function mutations were identified in humans and were associated with hyperphagia, early-onset obesity, low heart rate, reduced basal metabolic rate and severe insulin resistance (Pearce et al., 2013). *Ksr 2*^−/−^ mice display obesity, high insulin levels, and impaired glucose tolerance (Pearce et al., 2013). Obesity persisted in *Ksr 2*^−/−^ mice despite being fed the same amount of diet as *Ksr2*^+/+^ littermates (Pearce et al., 2013).

## Perspectives

To conclude this review, we provide helpful suggestions to accelerate the identification of obesity predisposing genes as well as their functional interrogation in mouse and human.

The use of approaches integrating multiple types of data (system biology/functional genomics) could boost the identification of genes predisposing to obesity (Yang, Jiang & He, 2009). Such studies could benefit from the use of mice to access tissues that are not readily available in humans or to perform deep phenotyping that is too expensive in humans. The co-mapping of gene expression levels (eQTLs), protein levels (pQTLs) and even metabolites (mQTLs) to a location in the genome associated with a disease may generate novel hypotheses for direct testing in humans and mice (Davis et al., 2012; Mackay, Stone & Ayroles, 2009; Yang et al., 2009). As an illustration, a study combining positional cloning and high-throughput transcriptome approaches identified two novel candidate genes driving adiposity in mice (*Akr1b8* and *Rgs2*) that deserve further investigation in humans (Derry et al., 2010).

Stringent P-value thresholds are classically used in GWAS (*P* < 5 × 10^−8^) to adjust the experiment for the many hypotheses tested (Dudbridge & Gusnanto, 2008). As a result, many true associations that do not reach stringent P-value thresholds are missed by conventional GWAS (Stahl et al., 2012). Similar issues are now experienced in next generation sequencing (NGS) studies (Do, Kathiresan & Abecasis, 2012). Identifying these true positive associations is challenging and so far has been addressed by the ever expanding meta-analyses (Stahl et al., 2012). Another way to ‘separate the wheat from the chaff’ could include the utilization of hypothesis-driven GWAS and next generation sequencing (NGS) approaches as opposed to hypothesis-free strategies (Li & Meyre, 2013). This method is beneficial in its decreased number of statistical tests and less stringent significance thresholds for the hypotheses being tested (Li & Meyre, 2013). Narrowing down the hypothesis to a specific linkage region or molecular pathway for example, could lead to identification of association signals previously missed by conventional GWAS (Li & Meyre, 2013). These high-throughput hypothesis-driven experiments would greatly benefit from the inclusion of data collected in mouse models of obesity (Li & Meyre, 2013).

It is also important to emphasize on the value of expression studies in future experiments. Expression studies in mice can add valuable knowledge of the expression and regulation of genes under diverse environmental exposures (Yoganathan, Karunakaran & Clee, 2012) especially when studying the expression of a gene in a certain tissue is difficult to obtain in human studies. In a recent study, the expression of a subset of GWAS obesity candidate genes was observed to be different in the hypothalamus and/or adipose tissue of fed vs. fasted animals (Yoganathan, Karunakaran & Clee, 2012). These experiments are helpful in moving from GWAS association signals to relevant candidate genes for obesity (Yoganathan, Karunakaran & Clee, 2012).

Improving on methodology and techniques used in animal studies could also provide better insight in upcoming genetics studies. Employing more recent tools in genome editing such as CRISPR/Cas9 could allow for more precise targeted mutagenesis (Zhang, Wen & Guo, 2014). This method depends on small RNA for sequence-specific cleavage (Jinek et al., 2012) for DNA targeting which is relatively cheap and easy to produce (Zhang, Wen & Guo, 2014). It involves a non-specific Cas9 nuclease and a set of programmable sequence-specific CRIPS RNA (crRNA) which can guide Cas9 to cleave the DNA and generate double-strand breaks at target sites (Zhang, Wen & Guo, 2014). CRISP/Cas9 is able to simultaneously allow for genomic modifications at multiple independent sites (Cong et al., 2013), but it can also induce non desired insertion deletions (Lin et al., 2014).

In light of improvement in animal models, utilizing tissue or time specific knockouts, knock-in or transgenic mice could help in better understanding the function of genes that were previously associated with obesity. For example, mice with global *PPAR γ* inactivation showed reduced adipose tissue and mild glucose intolerance (Koutnikova et al., 2003). In comparison, fat-specific *PPAR γ* knockout animals showed complete lipoatrophy, impaired adipokine secretion, profound insulin resistance and hyperglycemia, abnormal bone, mammary gland and skin metabolism (Wang et al., 2013). Although not frequently used in the obesity genetics field, time-specific knockouts are helpful in understanding gene expression at different developmental stages (Loebel et al., 2014). For instance, complete post-natal inactivation of *BDNF* in mice was associated with hyperphagic obesity, whereas pre-natal inactivation of the same gene was lethal (Rios et al., 2001).

Understanding the importance of gene-gene interactions in development of obesity is another key area of investigation. Studying gene-gene interactions in humans are experimentally demanding because they require large sample sizes to detect significant interactions, and are statistically challenging due to multiple testing issues (Hu, Wang & Wang, 2014). Therefore model organisms are an important tool for studying gene-gene interactions (MacKay, 2014). Genetic studies in mice may also facilitate the discovery of gene-gene interactions or loci whose effects are only evident in the context of specific alleles at other loci. This approach is based on the hypothesis that a QTL for a trait in mouse that maps to a homologous location for the same trait in humans, is most likely caused by the same gene (Leduc et al., 2011). The BSB mouse model is an example of mouse model used to study epistatic effects on obesity QTLs (Yi et al., 2004). Mapping and identification of gene × gene interactions in mice could be examined in humans, again, since the homologous regions of mouse and human chromosome regions are well-defined (Chiu et al., 2006). Double-knockout mouse models, where two genes are inactivated, can reveal valuable information about gene-gene interactions (Chiu et al., 2006). Double-knockout mice have not been commonly studied in obesity field, so we present an example from the diabetes field. *Irs*^1−/−^ have mild glucose intolerance and *Irs*^3−/−^ have no detectable phenotype, but *Irs*^1−/−^*/Irs*^3−/−^ are hyperglycemic and display severe lipoatrophy (Terauchi et al., 2003), indicative of their interaction in developing the type 2 diabetes phenotype. Complex mouse models showing evidence of epistasis can be tested in human genetic epidemiology studies. Although the number of known gene-gene interactions in human obesity is relatively small, we hope that increased sample size in epidemiological studies could help elucidate more of these interactions. Double knock out or transgenic mouse models are an attractive model to confirm the interactions identified in humans.

Aside from the importance of gene-gene interactions, recognizing the importance of gene-environment interactions in developing obesity is crucial (Andreasen & Andersen, 2009). For instance, physical activity could offset the aggregated genetic risk of multiple obesity loci (Ahmad et al., 2013; Li et al., 2010). Gene-environment studies can help in targeting populations that may respond well or poorly to a specific lifestyle or therapeutic interventions (Ahmad et al., 2013). Establishing large case-control studies in population-based cohorts, precise phenotyping of quantitative trait studies with precise measurements of lifestyle exposure, and target testing of interaction in existing lifestyle trials will provide the best understanding of gene-environment interactions (Franks et al., 2007).

To effectively demonstrate how genetic variations at a specific locus modify the effect of an environmental stimuli on a metabolic trait requires a combination of environmental modifications on animal models and human etiological trials (Franks & Roth, 2008). Different environmental modifications can be tested in homozygous or heterozygous knock out or knock in models and they can eventually be studied in inbred strains with naturally occurring allelic variations. Testing the gene-environment interactions in genes with prior evidence of their role in interacting with an environmental stimuli in mouse models can improve the probability that an observed effect is true (Franks & Roth, 2008), which may help to better control for the high likelihood of false positives in gene-environment interactions in human studies. Studying gene-environment interactions in knock-out animal models may be used as a first step to prioritize genes for gene environment interaction studies in human populations. For instance, *Gipr* knockout mice are protected against obesity and disturbance to their glucose homeostasis under a high-fat diet (Sonestedt et al., 2012). Human studies of *GIPR* common gene variants found a significant interaction between the rs10423928 SNP and a high-fat/low carbohydrate diet on risk of type 2 diabetes (Sonestedt et al., 2012).

Lastly, genes involved in diet and immune response have been preferential targets in positive selection during mammalian evolution, highlighting the importance of nutrient availability and pathogens as powerful driving forces of evolution (Kosiol & Vinar, 2008). Evolutionary assessment of mutations associated with highly penetrant from of obesity through mammal evolution (including rodents) may be complementary to *in vitro* and *in vivo* characterization studies in evaluating their functional relevance (Stäubert et al., 2007). This approach may be less relevant to common variants of obesity with modest effect. For instance, more than 90% of 70 missense mutations in *MC4R* identified in obese patients were located at amino acid positions that are highly conserved during 450 million years of MC4R evolution in vertebrates (Stäubert et al., 2007).

**Figure 3:**
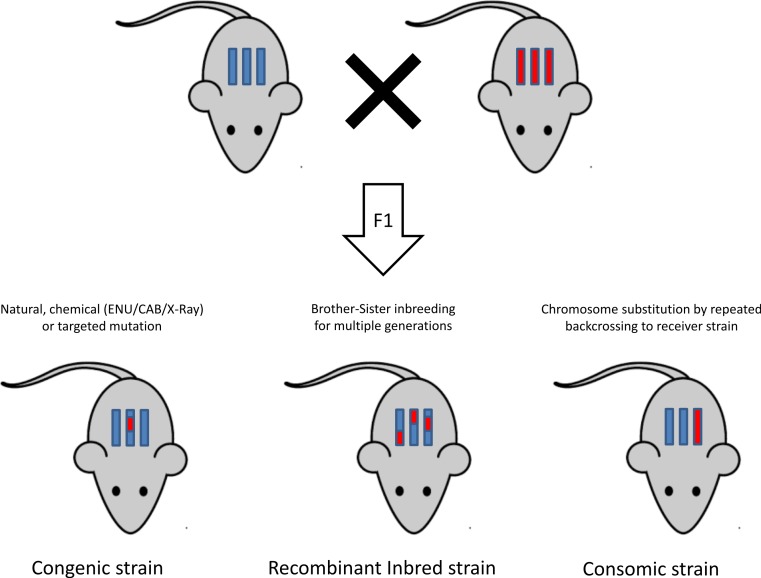
General overview of mutagenesis and inbreeding in mice. Congenic, recombinant inbred and consomic mice are obtained when part of the genome of one mouse strain is transferred to another strain by backcrossing the donor mouse to the receiver strain. In congenic mouse, the offspring resembles the parent strain except for the mutated chromosomal segment, whereas in consomic strain, the offspring carries an entire chromosome from the donor strain. Recombinant inbred strains are obtained by cross breeding of inbred mice to increase their genotypic diversity and carrying a series of brother-sister mating for multiple generations.

**Figure 4:**
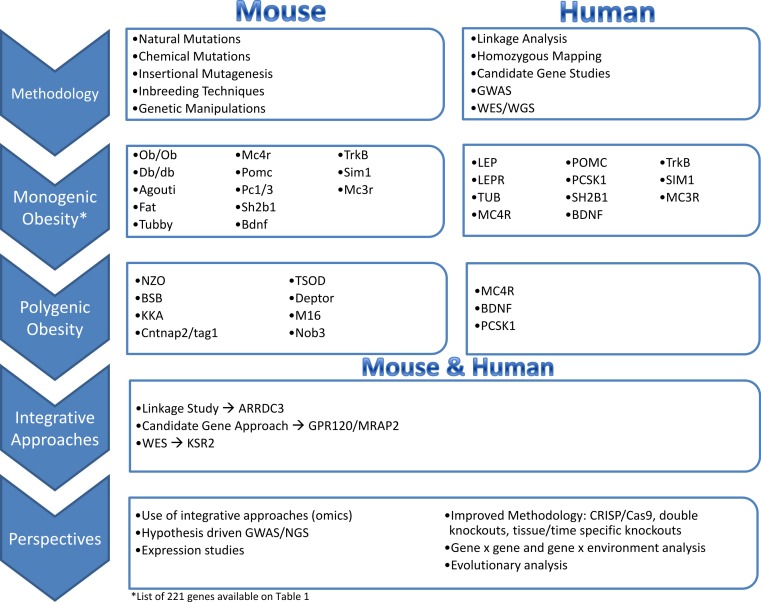
Graphical representation of the main concepts of the review. Summary of the major concepts in methodology of mouse include natural, chemical and insertional mutagenesis, as well as inbreeding and genetic manipulation techniques. In humans, linkage analysis, homozygous mapping, candidate gene studies, genome-wide association studies (GWAS) and whole exome/genome studies (WES/WGS) are discussed. These methodologies have led to genetic studies in monogenic and polygenic obesity, where mouse models paved the way for genetic discoveries in humans. The reverse concept also holds true, as genetic discoveries in humans led to development of new mouse models. In recent attempts of genetic discovery, an integrative approach of animal and human studies have promoted new gene discoveries as well as functional analysis. Current studies are utilizing innovative approaches such as use of omics, hypothesis driven GWAS, expression studies, improved genetic manipulation techniques, gene × gene or gene x environment analysis as well as evolutionary analysis to improve our understanding of the genetic architecture of obesity.

## Conclusions

We have reviewed the synthesis between mouse and human genetics in the field of obesity. We describe the approaches and techniques that are available for mouse and human geneticists, and provide a striking illustration of the synergy between these approaches that led to successful obesity causing gene identifications these last decades. We list innovative approaches to not only ensure a higher yield of novel obesity genes, but also a deeper understanding of their function. Figure 4 is an illustration that summarizes the discussions in this review paper. Integrative mouse human strategies have the potential to lead to the identification of more genes responsible for common Mendelian forms of obesity, as well as gene × gene and gene × environment interactions. This may help to unravel the missing heritability of obesity. We believe that an exhaustive understanding of obesity genetics will help to identify novel drug targets and to design more efficient and personalized obesity prevention and management programs that, with the support of populations and stakeholders, will ultimately curb the obesity epidemic (Agurs-Collins et al., 2008).

## References

[ref-1] Abney M, Ober C, McPeek MS (2002). Quantitative-trait homozygosity and association mapping and empirical genomewide significance in large, complex pedigrees: fasting serum-insulin level in the Hutterites. American Journal of Human Genetics.

[ref-2] Acevedo-Arozena A, Wells S, Potter P, Kelly M, Cox RD, Brown SDM (2008). ENU mutagenesis, a way forward to understand gene function. Annual Review of Genomics and Human Genetics.

[ref-3] Agurs-Collins T, Khoury MJ, Simon-Morton D, Olster DH, Harris JR, Milner JA (2008). Public health genomics: translating obesity genomics research into population health benefits. Obesity (Silver Spring, Md.).

[ref-4] Ahmad S, Rukh G, Varga TV, Ali A, Kurbasic A, Shungin D, Ericson U, Koivula RW, Chu AY, Rose LM, Ganna A, Qi Q, Stanáková A, Sandholt CH, Elks CE, Curhan G, Jensen MK, Tamimi RM, Allin KH, Jørgensen T, Brage S, Langenberg C, Aadahl M, Grarup N, Linneberg A, Paré G, Magnusson PKE, Pedersen NL, Boehnke M, Hamsten A, Mohlke KL, Pasquale LT, Pedersen O, Scott RA, Ridker PM, Ingelsson E, Laakso M, Hansen T, Qi L, Wareham NJ, Chasman DI, Hallmans G, Hu FB, Renström F, Orho-Melander M, Franks PW (2013). Gene × physical activity interactions in obesity: combined analysis of 111,421 individuals of European ancestry. PLoS Genetics.

[ref-5] Ahmad S, Varga T, Franks P (2013). Gene × environment interactions in obesity: the state of the evidence. Human Heredity.

[ref-6] Airik R, Slaats GG, Guo Z, Weiss A-C, Khan N, Ghosh A, Hurd TW, Bekker-Jensen S, Schrøder JM, Elledge SJ, Andersen JS, Kispert A, Castelli M, Boletta A, Giles RH, Hildebrandt F (2014). Renal-retinal ciliopathy gene Sdccag8 regulates DNA damage response signaling. Journal of the American Society of Nephrology.

[ref-7] Allan MF, Eisen EJ, Pomp D (2004). The M16 mouse: an outbred animal model of early onset polygenic obesity and diabesity. Obesity Research.

[ref-8] An D, Toyoda T, Taylor EB, Yu H, Fujii N, Hirshman MF, Goodyear LJ (2010). Glucose transport in mouse skeletal muscle. Diabetes.

[ref-9] Andreasen CH, Andersen G (2009). Gene-environment interactions and obesity–further aspects of genomewide association studies. Nutrition (Burbank, Los Angeles County, Calif.).

[ref-10] Arya R, Duggirala R, Jenkinson CP, Almasy L, Blangero J, O’Connell P, Stern MP (2004). Evidence of a novel quantitative-trait locus for obesity on chromosome 4p in Mexican Americans. American Journal of Human Genetics.

[ref-11] Asai M, Ramachandrappa S, Joachim M, Shen Y, Zhang R, Nuthalapati N, Ramanathan V, Strochlic DE, Ferket P, Linhart K, Ho C, Novoselova TV, Garg S, Ridderstrale M, Marcus C, Hirschhorn JN, Keogh JM, O’Rahilly S, Chan LF, Clark AJ, Farooqi IS, Majzoub JA (2013). Loss of function of the melanocortin 2 receptor accessory protein 2 is associated with mammalian obesity. Science.

[ref-12] Atwood LD, Heard-Costa NL, Cupples LA, Jaquish CE, Wilson PWF, D’Agostino RB (2002). Genomewide linkage analysis of body mass index across 28 years of the Framingham Heart Study. American Journal of Human Genetics.

[ref-13] Ayala JE, Samuel VT, Morton GJ, Obici S, Croniger CM, Shulman GI, Wasserman DH, McGuinness OP (2010). Standard operating procedures for describing and performing metabolic tests of glucose homeostasis in mice. Disease Models and Mechanisms.

[ref-14] Aylor DL, Valdar W, Foulds-Mathes W, Buus RJ, Verdugo RA, Baric RS, Ferris MT, Frelinger JA, Heise M, Frieman MB, Gralinski LE, Bell TA, Didion JD, Hua K, Nehrenberg DL, Powell CL, Steigerwalt J, Xie Y, Kelada SNP, Collins FS, Yang IV, Schwartz DA, Branstetter LA, Chesler EJ, Miller DR, Spence J, Liu EY, McMillan L, Sarkar A, Wang J, Wang W, Zhang Q, Broman KW, Korstanje R, Durrant C, Mott R, Iraqi FA, Pomp D, Threadgill D, Pardo-Manuel de Villena F, Churchill GA (2011). Genetic analysis of complex traits in the emerging Collaborative Cross. Genome Research.

[ref-15] Bachmann-Gagescu R, Mefford HC, Cowan C, Glew GM, Hing AV, Wallace S, Bader PI, Hamati A, Reitnauer PJ, Smith R, Stockton DW, Muhle H, Helbig I, Eichler EE, Ballif BC, Rosenfeld J, Tsuchiya KD (2010). Recurrent 200-kb deletions of 16p11.2 that include the SH2B1 gene are associated with developmental delay and obesity. Genetics in Medicine.

[ref-16] Bahreini N, Noor MI, Koon PB, Talib RA, Lubis SH, Dashti MG, Salehi-Abargouei A, Esmaillzadeh A (2013). Weight status among Iranian adolescents: comparison of four different criteria. Journal of Research in Medical Sciences: The Official Journal of Isfahan University of Medical Sciences.

[ref-17] Beales P (2010). Obesity in single gene disorder. Genes and obesity.

[ref-18] Belizário JE, Akamini P, Wolf P, Strauss B, Xavier-Neto J (2012). New routes for transgenesis of the mouse. Journal of Applied Genetics.

[ref-19] Bennett HP (1986). Biosynthetic fate of the amino-terminal fragment of pro-opiomelanocortin within the intermediate lobe of the mouse pituitary. Peptides.

[ref-20] Benzinou M, Creemers JWM, Choquet H, Lobbens S, Dina C, Durand E, Guerardel A, Boutin P, Jouret B, Heude B, Balkau B, Tichet J, Marre M, Potoczna N, Horber F, Le Stunff C, Czernichow S, Sandbaek A, Lauritzen T, Borch-Johnsen K, Andersen G, Kiess W, Körner A, Kovacs P, Jacobson P, Carlsson LMS, Walley AJ, Jørgensen T, Hansen T, Pedersen O, Meyre D, Froguel P (2008). Common nonsynonymous variants in PCSK1 confer risk of obesity. Nature Genetics.

[ref-21] Berndt SI, Gustafsson S, Mägi R, Ganna A, Wheeler E, Feitosa MF, Justice AE, Monda KL, Croteau-Chonka DC, Day FR, Esko T, Fall T, Ferreira T, Gentilini D, Jackson AU, Luan J, Randall JC, Vedantam S, Willer CJ, Winkler TW, Wood AR, Workalemahu T, Hu Y-J, Lee SH, Liang L, Lin D-Y, Min JL, Neale BM, Thorleifsson G, Yang J, Albrecht E, Amin N, Bragg-Gresham JL, Cadby G, den Heijer M, Eklund N, Fischer K, Goel A, Hottenga J-J, Huffman JE, Jarick I, Johansson Å, Johnson T, Kanoni S, Kleber ME, König IR, Kristiansson K, Kutalik Z, Lamina C, Lecoeur C, Li G, Mangino M, McArdle WL, Medina-Gomez C, Müller-Nurasyid M, Ngwa JS, Nolte IM, Paternoster L, Pechlivanis S, Perola M, Peters MJ, Preuss M, Rose LM, Shi J, Shungin D, Smith AV, Strawbridge RJ, Surakka I, Teumer A, Trip MD, Tyrer J, Van Vliet-Ostaptchouk JV, Vandenput L, Waite LL, Zhao JH, Absher D, Asselbergs FW, Atalay M, Attwood AP, Balmforth AJ, Basart H, Beilby J, Bonnycastle LL, Brambilla P, Bruinenberg M, Campbell H, Chasman DI, Chines PS, Collins FS, Connell JM, Cookson WO, de Faire U, de Vegt F, Dei M, Dimitriou M, Edkins S, Estrada K, Evans DM, Farrall M, Ferrario MM, Ferrières J, Franke L, Frau F, Gejman PV, Grallert H, Grönberg H, Gudnason V, Hall AS, Hall P, Hartikainen A-L, Hayward C, Heard-Costa NL, Heath AC, Hebebrand J, Homuth G, Hu FB, Hunt SE, Hyppönen E, Iribarren C, Jacobs KB, Jansson J-O, Jula A, Kähönen M, Kathiresan S, Kee F, Khaw K-T, Kivimäki M, Koenig W, Kraja AT, Kumari M, Kuulasmaa K, Kuusisto J, Laitinen JH, Lakka TA, Langenberg C, Launer LJ, Lind L, Lindström J, Liu J, Liuzzi A, Lokki M-L, Lorentzon M, Madden PA, Magnusson PK, Manunta P, Marek D, März W, Leach IM, McKnight B, Medland SE, Mihailov E, Milani L, Montgomery GW, Mooser V, Mühleisen TW, Munroe PB, Musk AW, Narisu N, Navis G, Nicholson G, Nohr EA, Ong KK, Oostra BA, Palmer CNA, Palotie A, Peden JF, Pedersen N, Peters A, Polasek O, Pouta A, Pramstaller PP, Prokopenko I, Pütter C, Radhakrishnan A, Raitakari O, Rendon A, Rivadeneira F, Rudan I, Saaristo TE, Sambrook JG, Sanders AR, Sanna S, Saramies J, Schipf S, Schreiber S, Schunkert H, Shin S-Y, Signorini S, Sinisalo J, Skrobek B, Soranzo N, Stanáková A, Stark K, Stephens JC, Stirrups K, Stolk RP, Stumvoll M, Swift AJ, Theodoraki EV, Thorand B, Tregouet D-A, Tremoli E, Van der Klauw MM, van Meurs JBJ, Vermeulen SH, Viikari J, Virtamo J, Vitart V, Waeber G, Wang Z, Widén E, Wild SH, Willemsen G, Winkelmann BR, Witteman JCM, Wolffenbuttel BHR, Wong A, Wright AF, Zillikens MC, Amouyel P, Boehm BO, Boerwinkle E, Boomsma DI, Caulfield MJ, Chanock SJ, Cupples LA, Cusi D, Dedoussis GV, Erdmann J, Eriksson JG, Franks PW, Froguel P, Gieger C, Gyllensten U, Hamsten A, Harris TB, Hengstenberg C, Hicks AA, Hingorani A, Hinney A, Hofman A, Hovingh KG, Hveem K, Illig T, Jarvelin M-R, Jöckel K-H, Keinanen-Kiukaanniemi SM, Kiemeney LA, Kuh D, Laakso M, Lehtimäki T, Levinson DF, Martin NG, Metspalu A, Morris AD, Nieminen MS, Njølstad I, Ohlsson C, Oldehinkel AJ, Ouwehand WH, Palmer LJ, Penninx B, Power C, Province MA, Psaty BM, Qi L, Rauramaa R, Ridker PM, Ripatti S, Salomaa V, Samani NJ, Snieder H, Sørensen TIA, Spector TD, Stefansson K, Tönjes A, Tuomilehto J, Uitterlinden AG, Uusitupa M, van der Harst P, Vollenweider P, Wallaschofski H, Wareham NJ, Watkins H, Wichmann H-E, Wilson JF, Abecasis GR, Assimes TL, Barroso I, Boehnke M, Borecki IB, Deloukas P, Fox CS, Frayling T, Groop LC, Haritunian T, Heid IM, Hunter D, Kaplan RC, Karpe F, Moffatt MF, Mohlke KL, O’Connell JR, Pawitan Y, Schadt EE, Schlessinger D, Steinthorsdottir V, Strachan DP, Thorsteinsdottir U, van Duijn CM, Visscher PM, Di Blasio AM, Hirschhorn JN, Lindgren CM, Morris AP, Meyre D, Scherag A, McCarthy MI, Speliotes EK, North KE, Loos RJF, Ingelsson E (2013). Genome-wide meta-analysis identifies 11 new loci for anthropometric traits and provides insights into genetic architecture. Nature Genetics.

[ref-22] Bessesen DH (2008). Update on obesity. The Journal of Clinical Endocrinology and Metabolism.

[ref-23] Biebermann H, Castañeda TR, van Landeghem F, von Deimling A, Escher F, Brabant G, Hebebrand J, Hinney A, Tschöp MH, Grüters A, Krude H (2006). A role for beta-melanocyte-stimulating hormone in human body-weight regulation. Cell Metabolism.

[ref-24] Boch J (2011). News and views TALEs of genome targeting A mucosal gateway for vaccines. Nature Biotechnology.

[ref-25] Bochukova EG, Huang N, Keogh J, Henning E, Purmann C, Blaszczyk K, Saeed S, Hamilton-Shield J, Clayton-Smith J, O’Rahilly S, Hurles ME, Farooqi IS (2010). Large, rare chromosomal deletions associated with severe early-onset obesity. Nature.

[ref-26] Bonaglia MC, Ciccone R, Gimelli G, Gimelli S, Marelli S, Verheij J, Giorda R, Grasso R, Borgatti R, Pagone F, Rodrìguez L, Martinez-Frias M-L, van Ravenswaaij C, Zuffardi O (2008). Detailed phenotype-genotype study in five patients with chromosome 6q16 deletion: narrowing the critical region for Prader-Willi-like phenotype. European Journal of Human Genetics.

[ref-27] Bonnefond A, Raimondo A, Stutzmann F, Ghoussaini M, Ramachandrappa S, Bersten DC, Durand E, Vatin V, Balkau B, Lantieri O, Raverdy V, Pattou F, Van Hul W, Van Gaal L, Peet DJ, Weill J, Miller JL, Horber F, Goldstone AP, Driscoll DJ, Bruning JB, Meyre D, Whitelaw ML, Froguel P (2013). Brief report loss-of-function mutations in SIM1 contribute to obesity Prader-Willi—like features. Journal of Clinical Investigation.

[ref-28] Borman AD, Pearce LR, Mackay DS, Nagel-Wolfrum K, Davidson AE, Henderson R, Garg S, Waseem NH, Webster AR, Plagnol V, Wolfrum U, Farooqi IS, Moore AT (2014). A homozygous mutation in the TUB gene associated with retinal dystrophy and obesity. Human Mutation.

[ref-29] Boston BA, Blaydon KM, Varnerin J, Cone RD (1997). Independent and additive effects of central POMC and leptin pathways on murine obesity. Science.

[ref-30] Brown CY, Sadlon T, Gargett T, Melville E, Zhang R, Drabsch Y, Ling M, Strathdee CA, Gonda TJ, Barry SC (2010). Robust reversible gene knockdown using a sing lentiviral short hairpin RNA vector. Human Gene Therapy.

[ref-31] Buchner DA, Geisinger JM, Glazebrook PA, Morgan MG, Nadeau JH (2012). The juxtaparanodal proteins CNTNAP2 and TAG1 regulate dietinduced obesity. Mammalian Genome.

[ref-32] Bultman SJ, Michaud EJ, Woychik RP (1992). Molecular characterization of the mouse agouti locus. Cell.

[ref-33] Butler AA, Kesteson RA, Khong K, Cullen MJ, Pelleymounter MA, Dekoning J, Baetscher M, Cone RD (2000). A unique metabolic syndrome causes obesity in the Melanocortin-3 receptor-deficient mouse. Endocrinology.

[ref-34] Butler A, Kozak LP (2010). A recurring problem with the analysis of energy expenditure in genetic models expressing lean and obese phenotypes. Diabetes.

[ref-35] Calton MA, Ersoy BA, Zhang S, Kane JP, Malloy MJ, Pullinger CR, Bromberg Y, Pennacchio LA, Dent R, McPherson R, Ahituv N, Vaisse C (2009). Association of functionally significant Melanocortin-4 but not Melanocortin-3 receptor mutations with severe adult obesity in a large North American case-control study. Human Molecular Genetics.

[ref-36] Carroll K, Gomez C, Shapiro L (2004). Tubby proteins: the plot thickens. Nature Reviews. Molecular Cell Biology.

[ref-37] Casola S (2010). Mouse models for miRNA expression: the ROSA26 locus. Methods in Molecular Biology.

[ref-38] Chadt A, Leicht K, Deshmukh A, Jiang LQ, Scherneck S, Bernhardt U, Dreja T, Vogel H, Schmolz K, Kluge R, Zierath JR, Hultschig C, Hoeben RC, Schürmann A, Joost H-G, Al-Hasani H (2008). Tbc1d1 mutation in lean mouse strain confers leanness protects from diet-induced obesity. Nature Genetics.

[ref-39] Challis BG, Pritchard LE, Creemers JWM, Delplanque J, Keogh JM, Luan J, Wareham NJ, Yeo GSH, Bhattacharyya S, Froguel P, White A, Sadaf Farooqi I, O’Rahilly S (2002). A missense mutation disrupting a dibasic prohormone processing site in pro-opiomelanocortin (POMC) increases susceptibility to early-onset obesity through a novel molecular mechanism. Human Molecular Genetics.

[ref-40] Chan LF, Webb TR, Chung T-T, Meimaridou E, Cooray SN, Guasti L, Chapple JP, Egertova M, Elphick MR, Cheetham ME, Metherell LA, Clark AJL (2009). MRAP and MRAP2 are bidirectional regulators of the melanocortin receptor family. Proceedings of the National Academy of Sciences of the United States of America.

[ref-41] Chen H, Charlat O, Tartaglia LA, Woolf EA, Weng X, Ellis SJ, Lakey ND, Culpepper J, More KJ, Breitbart RE, Duyk GM, Tepper RI, Morgenstern JP (1996). Evidence that the diabetes gene encodes the leptin receptor: identification of a mutation in the leptin receptor gene in db/db mice. Cell.

[ref-42] Chen AS, Marsh DJ, Trumbauer ME, Frazier EG, Guan XM, Yu H, Rosenblum CI, Vongs A, Feng Y, Cao L, Metzger JM, Strack AM, Camacho RE, Mellin TN, Nunes CN, Min W, Fisher J, Gopal-Truter S, Euan MacIntyre D, Howard Y, Chen LHT, Van der Ploeg (2000). Inactivation of the mouse melanocortin-3 receptor results in increased fat mass and reduced lean body mass. Nature Genetics.

[ref-43] Chiu S, Diament A, Fisler J, Warden C (2006). Gene–gene epistasis and gene-environment interactions influence diabetes and obesity. Nutritional genomics: discovering the path to personalized nutrition.

[ref-44] Choquet H, Meyre D (2011). Genetics of obesity: what have we learned?. Current Genomics.

[ref-45] Church C, Lee S, Bagg EAL, McTaggart JS, Deacon R, Gerken T, Lee A, Moir L, Mecinov J, Quwailid MM, Schofield CJ, Ashcroft FM, Cox RD (2009). A mouse model for the metabolic effects of the human fat mass and obesity associated FTO gene. PLoS Genetics.

[ref-46] Church C, Moir L, McMurray F, Girard C, Banks GT, Teboul L, Wells S, Brüning JC, Nolan PM, Ashcroft FM, Cox RD (2010). Overexpression of Fto leads to increased food intake results in obesity. Nature Genetics.

[ref-47] Cirillo G, Marini R, Ito S, Wakamatsu K, Scianguetta S, Bizzarri C, Romano A, Grandone A, Perrone L, Cappa M, Miraglia del Giudice E (2012). Lack of red hair phenotype in a North-African obese child homozygous for a novel POMC null mutation: nonsense-mediated decay RNA evaluation hair pigment chemical analysis. The British Journal of Dermatology.

[ref-48] Clee SM, Attie AD (2007). The genetic landscape of type 2 diabetes in mice. Endocrine Reviews.

[ref-49] Clément K, Dubern B, Mencarelli M, Czernichow P, Ito S, Wakamatsu K, Barsh GS, Vaisse C, Leger J (2008). Unexpected endocrine features normal pigmentation in a young adult patient carrying a novel homozygous mutation in the POMC gene. The Journal of Clinical Endocrinology and Metabolism.

[ref-50] Clément K, Vaisse C, Lahlou N, Cabrol S, Pelloux V, Cassuto D, Gourmelen M, Dina C, Chambaz J, Lacorte J-M, Basdevant A, Bougnéres P, Lebouc Y, Froguel P, Guy-Grand B (1998). A mutation in the human leptin receptor gene causes obesity and pituitary dysfunction. Nature.

[ref-51] Coleman DL (1973). Effects of parabiosis of obese with diabetes and normal mice. Diabetologia.

[ref-52] Coleman DL (1982). Diabetes-obesity syndromes in mice. Diabetes.

[ref-53] Coleman DL, Eicher EM (1990). Fat (fat) and tubby (tub): two autosomal recessive mutations causing obesity syndromes in the mouse. The Journal of Heredity.

[ref-54] Coleman DL, Hummel KP (1967). Studies with the mutation, diabetes, in the mouse. Diabetologia.

[ref-55] Coleman DL, Hummel K (1973). The influence of genetic background on the expression of the obese. Diabetologia.

[ref-56] Cong L, Ran FA, Cox D, Lin S, Barretto R, Habib N, Hsu PD, Wu X, Jiang W, Marraffini LA, Zhang F (2013). Multiplex genome engineering using CRISPR/Cas systems. Science.

[ref-57] Couturier C, Sarkis C, Seron K, Belouzard S, Chen P, Lenain A, Corset L, Dam J, Vauthier V, Dubart A, Mallet J, Froguel P, Rouille Y, Jockers R (2007). Silencing of OB-RGRP in mouse hypothalamic arcuate nucleus increases leptin receptor signaling and prevents diet-induced obesity. Proceedings of the National Academy of Sciences of the United States of America.

[ref-58] Cox RD, Church CD (2011). Mouse models and the interpretation of human GWAS in type 2 diabetes and obesity. Disease Models and Mechanisms.

[ref-59] Creemers JWM, Choquet H, Stijnen P, Vatin V, Pigeyre M, Beckers S, Meulemans S, Than ME, Yengo L, Tauber M, Balkau B, Elliott P, Jarvelin M-R, Van Hul W, Van Gaal L, Horber F, Pattou F, Froguel P, Meyre D (2012). Heterozygous mutations causing partial prohormone convertase 1 deficiency contribute to human obesity. Diabetes.

[ref-60] Creemers JW, Jackson RS, Hutton JC (1998). Molecular and cellular regulation of prohormone processing. Seminars in Cell and Developmental Biology.

[ref-61] Crews ST (1998). Control of cell lineage-specific development and transcription by bHLH-PAS proteins. Genes and Development.

[ref-62] Croft JB, Morrell D, Chase CL, Swift M (1995). Obesity in heterozygous carriers of the gene for the Bardet-Biedl syndrome. American Journal of Medical Genetics.

[ref-63] Davenport JR, Watts AJ, Roper VC, Croyle MJ, van Groen T, Wyss JM, Nagy TR, Kesterson RA, Yoder BK (2007). Disruption of intraflagellar transport in adult mice leads to obesity slow-onset cystic kidney disease. Current Biology.

[ref-64] Davey RA, MacLean HE (2006). Current and future approaches using genetically modified mice in endocrine research. American Journal of Physiology. Endocrinology and Metabolism.

[ref-65] Davis RC, van Nas A, Castellani LW, Zhao Y, Zhou Z, Wen P, Yu S, Qi H, Rosales M, Schadt EE, Broman KW, Peterfy M, Lusis AJ (2012). Systems genetics of susceptibility to obesity-induced diabetes in mice. Physiological Genomics.

[ref-66] Demenais F, Kanninen T, Lindgren CM, Wiltshire S, Gaget S, Dandrieux C, Almgren P, Sjogren M, Hattersley A, Dina C, Tuomi T, McCarthy MI, Froguel P, Groop LC (2003). A meta-analysis of four European genome screens (GIFT Consortium) shows evidence for a novel region on chromosome 17p11.2-q22 linked to type 2 diabetes. Human Molecular Genetics.

[ref-67] Derry JMJ, Zhong H, Molony C, MacNeil D, Guhathakurta D, Zhang B, Mudgett J, Small K, El Fertak L, Guimond A, Selloum M, Zhao W, Champy MF, Monassier L, Vogt T, Cully D, Kasarskis A, Schadt EE (2010). Identification of genes and networks driving cardiovascular and metabolic phenotypes in a mouse F2 intercross. PLoS ONE.

[ref-68] De Souza AT, Dai X, Spencer AG, Reppen T, Menzie A, Roesch PL, He Y, Caguyong MJ, Bloomer S, Herweijer H, Wolff JA, Hagstrom JE, Lewis DL, Linsley PS, Ulrich RG (2006). Transcriptional phenotypic comparisons of Ppara knockout siRNA knockdown mice. Nucleic Acids Research.

[ref-69] Diament AL, Fisler JS, Warden CH (2003). Studies of natural allele effects in mice can be used to identify genes causing common human obesity. Obesity Reviews.

[ref-70] Dickies MM (1962). A new viable yellow mutation in the house mouse. The Journal of Heredity.

[ref-71] Dickson SP, Wang K, Krantz I, Hakonarson H, Goldstein DB (2010). Rare variants create synthetic genome-wide associations. PLoS Biology.

[ref-72] Dina C, Meyre D, Gallina S, Durand E, Körner A, Jacobson P, Carlsson LMS, Kiess W, Vatin V, Lecoeur C, Delplanque J, Vaillant E, Pattou F, Ruiz J, Weill J, Levy-Marchal C, Horber F, Potoczna N, Hercberg S, Le Stunff C, Bougnères P, Kovacs P, Marre M, Balkau B, Cauchi S, Chèvre J-C, Froguel P (2007). Variation in FTO contributes to childhood obesity and severe adult obesity. Nature Genetics.

[ref-73] Dittgen T, Nimmerjahn A, Komai S, Licznerski P, Waters J, Margrie TW, Helmchen F, Denk W, Brecht M, Osten P (2004). Lentivirus-based genetic manipulations of cortical neurons their optical electrophysiological monitoring *in vivo*. Proceedings of the National Academy of Sciences of the United States of America.

[ref-74] Do R, Kathiresan S, Abecasis GR (2012). Exome sequencing and complex disease: practical aspects of rare variant association studies. Human Molecular Genetics.

[ref-75] Doche ME, Bochukova EG, Su H, Pearce LR, Keogh JM, Henning E, Cline JM, Saeed S, Dale A, Cheetham T, Barroso I, Argetsinger LS, O’Rahilly S, Rui L, Carter-Su C, Farooqi IS (2012). Human SH2B1 mutations are associated with maladaptive behaviors and obesity. The Journal of Clinical Invesitigation.

[ref-76] Dokas J, Chadt A, Nolden T, Himmelbauer H, Zierath JR, Joost H-G, Al-Hasani H (2013). Conventional knockout of Tbc1d1 in mice impairs insulin- and AICAR-stimulated glucose uptake in skeletal muscle. Endocrinology.

[ref-77] Dong H, Maddux BA, Altomonte J, Meseck M, Accili D, Terkeltaub R, Johnson K, Youngren JF, Goldfine ID (2005). Increased hepatic levels of the insulin receptor inhibitor, PC-1/NPP1, induce insulin resistance and glucose intolerance. Diabetes.

[ref-78] Dougherty MK, Ritt DA, Zhou M, Specht S, Monson DM, Veenstra TD, Morrison DK (2010). KSR2 is a calcineurin substrate that promotes ERK cascade activation in response to calcium signals. Molecular and Cellular Biology.

[ref-79] Dubern B, Lubrano-Berthelier C, Mencarelli M, Ersoy B, Frelut M-L, Bouglé D, Costes B, Simon C, Tounian P, Vaisse C, Clement K (2008). Mutational analysis of the pro-opiomelanocortin gene in French obese children led to the identification of a novel deleterious heterozygous mutation located in the alpha-melanocyte stimulating hormone domain. Pediatric Research.

[ref-80] Dudbridge F, Gusnanto A (2008). Estimation of significance thresholds for genomewide association scans. Genetic Epidemiology.

[ref-81] Duggirala R, Blangero J, Almasy L, Arya R, Dyer TD, Williams KL, Leach RJ, O’Connell P, Stern MP (2001). A major locus for fasting insulin concentrations insulin resistance on chromosome 6q with strong pleiotropic effects on obesity-related phenotypes in nondiabetic Mexican Americans. American Journal of Human Genetics.

[ref-82] Duhl DM, Vrieling H, Miller KA, Wolff GL, Barsh GS (1994). Neomorphic agouti mutations in obese yellow mice. Nature Genetics.

[ref-83] Efthimiou M, Andrianopoulos C, Stephanou G, Demopoulos NA, Nikolaropoulos SS (2007). Aneugenic potential of the nitrogen mustard analogues melphalan, chlorambucil and p-N,N-bis(2-chloroethyl)aminophenylacetic acid in cell cultures *in vitro*. Mutation Research.

[ref-84] Ehm MG, Karnoub MC, Sakul H, Gottschalk K, Holt DC, Weber JL, Vaske D, Briley D, Briley L, Kopf J, McMillen P, Nguyen Q, Reisman M, Lai EH, Joslyn G, Shepherd NS, Bell C, Wagner MJ, Burns DK (2000). Genomewide search for type 2 diabetes susceptibility genes in four American populations. American Journal of Human Genetics.

[ref-85] Elbashir SM, Harborth J, Lendeckel W, Yalcin A, Weber K, Tuschl T (2001). Duplexes of 21-nucleotide RNAs mediate RNA interference in cultured mammalian cells. Nature.

[ref-86] Ellacott KLJ, Morton GJ, Woods SC, Tso P, Schwartz MW (2010). Assessment of feeding behavior in laboratory mice. Cell Metabolism.

[ref-87] Erickson JC, Hollopeter G, Palmiter RD (1996). Attenuation of the obesity syndrome of ob/ob mice by the loss of neuropeptide Y. Science.

[ref-88] Erikson J, Clegg K, Palmiter R (1996). Sensitivity to leptin and susceptibility to seizures of mice lacking neuropeptide Y. Nature.

[ref-89] Farooqi IS, Bullmore E, Keogh J, Gillard J, Rahilly SO, Fletcher PC (2007a). Leptin regulates striatal regions and human eating behavior. Science.

[ref-90] Farooqi IS, Drop S, Clements A, Keogh JM, Biernacka J, Lowenbein S, Challis BG, O’Rahilly S (2006). Heterozygosity for a POMC-null mutation increased obesity risk in humans. Diabetes.

[ref-91] Farooqi IS, Jebb SA, Langmack G, Lawrence E, Cheetham CH, Prentice AM, Hughes IA, McCamish MA, O’Rahilly S (1999). Effects of recombinant leptin therapy in a child with congenital leptin deficiency. New England Journal of Medicine.

[ref-92] Farooqi IS, Keogh JM, Yeo GSH, Lank EJ, Cheetham T (2003). Clinical Spectrum of Obesity and Mutations in the Melanocortin 4 Receptor Gene. New England Journal of Medicine.

[ref-93] Farooqi IS, Matarese G, Lord GM, Keogh JM, Lawrence E, Agwu C, Sanna V, Jebb SA, Perna F, Fontana S, Lechler RI, DePaoli AM, O’Rahilly S (2002). Beneficial effects of leptin on obesity, T cell hyporesponsiveness, neuroendocrine/metabolic dysfunction of human congenital leptin deficiency. Journal of Clinical Investigation.

[ref-94] Farooqi IS, O’Rahilly S (2008). Mutations in ligands and receptors of the leptin–melanocortin pathway that lead to obesity. Nature Reviews Endocrinology.

[ref-95] Farooqi IS, Volders K, Stanhope R, Heuschkel R, White A, Lank E, Keogh J, O’Rahilly S, Creemers JWM (2007b). Hyperphagia and early-onset obesity due to a novel homozygous missense mutation in prohormone convertase 1/3. The Journal of Clinical Endocrinology Metabolism.

[ref-96] Farooqi IS, Wangensteen T, Collins S, Kimber W, Matarese G, Keogh JM, Lank E, Bottomley B, Lopez-Fernandez J, Ferraz-Amaro I, Dattani MT, Ercan O, Myhre AG, Retterstol L, Stanhope R, Edge JA, McKenzie S, Lessan N, Ghodsi M, De Rosa V, Perna F, Fontana S, Barroso I, Undlien DE, O’Rahilly S (2007c). Clinical and molecular genetic spectrum of congenital deficiency of the leptin receptor. The New England Journal of Medicine.

[ref-97] Farooqi IS, Yeo GSH, Keogh JM, Aminian S, Jebb SA, Butler G, Cheetham T, O’Rahilly S (2000). Dominant and recessive inheritance of morbid obesity associated with melanocortin 4 receptor deficiency. The Journal of Clinical Investigation.

[ref-98] Fawcett GL, Roseman CC, Jarvis JP, Wang B, Wolf JB, Cheverud JM (2008). Genetic architecture of adiposity and organ weight using combined generation QTL analysis. Obesity.

[ref-99] Fischer J, Koch L, Emmerling C, Vierkotten J, Peters T, Brüning JC, Rüther U (2009). Inactivation of the Fto gene protects from obesity. Nature.

[ref-100] Fisler JS, Warden CH, Pace MJ, Lusis AJ (1993). BSB: a new mouse model of multigenic obesity. Obesity Research.

[ref-101] Flint J, Eskin E (2013). Genome wide association studies in mice. Nature Reviews Genetics.

[ref-102] Frank GR, Fox J, Candela N, Jovanovic Z, Bochukova E, Levine J, Fox J, Candela N, Jovanovic Z, Bochukova E, Levine J, Papenhausen PR, O’Rahilly S, Farooqi IS (2013). Severe obesity and diabetes insipidus in a patient with PCSK1 deficiency. Molecular Genetics and Metabolism.

[ref-103] Franks PW, Mesa J-L, Harding AH, Wareham NJ (2007). Gene-lifestyle interaction on risk of type 2 diabetes. Nutrition, Metabolism, and Cardiovascular Diseases?.

[ref-104] Franks PW, Roth SM (2008). Interaction between physical activity and genetic factors in complex metabolic disease. Physical activity and genetic factors in complex metabolic disease.

[ref-105] Frayling TM, Timpson NJ, Weedon MN, Zeggini E, Freathy RM, Lindgren CM, Perry JRB, Elliott KS, Lango H, Rayner NW, Shields B, Harries LW, Barrett JC, Ellard S, Groves CJ, Knight B, Patch A-M, Ness AR, Ebrahim S, Lawlor DA, Ring SM, Ben-Shlomo Y, Jarvelin M-R, Sovio U, Bennett AJ, Melzer D, Ferrucci L, Loos RJF, Barroso I, Wareham NJ, Karpe F, Owen KR, Cardon LR, Walker M, Hitman GA, Palmer CNA, Doney ASF, Morris AD, Smith GD, Hattersley AT, McCarthy MI, The Wellcome Trust Case Control Consortium (2007). A common variant in the FTO gene is associated with body mass index and predisposes to childhood and adult obesity. Science.

[ref-106] Fredriksson R, Hägglund M, Olszewski PK, Stephansson O, Jacobsson JA, Olszewska AM, Levine AS, Lindblom J, Schiöth HB (2008). The obesity gene FTO is of ancient origin up-regulated during food deprivation expressed in neurons of feeding-related nuclei of the brain. Endocrinology.

[ref-107] Freedman BD, Lee E-J, Park Y, Jameson JL (2005). A dominant negative peroxisome proliferator-activated receptor-gamma knock-in mouse exhibits features of the metabolic syndrome. The Journal of Biological Chemistry.

[ref-108] Gantz I, Miwa H, Konda Y, Shimoto Y, Tashiro T, Watson SJ, DelValle J, Yamada T (1993). Molecular cloning expression gene localization of a fourth melanocortin receptor. The Journal of Biological Chemistry.

[ref-109] Geller F, Reichwald K, Dempfle A, Illig T, Vollmert C, Herpertz S, Siffert W, Platzer M, Hess C, Gudermann T (2004). Melanocortin-4 receptor gene variant I103 is negatively associated with obesity. American Journal of Human Genetics.

[ref-110] Ghosh S, Watanabe RM, Valle TT, Hauser ER, Magnuson VL, Langefeld CD, Ally DS, Mohlke KL, Silander K, Kohtamäki K, Chines P, Balow J, Birznieks G, Chang J, Eldridge W, Erdos MR, Karanjawala ZE, Knapp JI, Kudelko K, Martin C, Morales-Mena A, Musick A, Musick T, Pfahl C, Porter R, Rayman JB (2000). The Finland-United States investigation of non-insulin-dependent diabetes mellitus genetics (FUSION) study. I. An autosomal genome scan for genes that predispose to type 2 diabetes. American Journal of Human Genetics.

[ref-111] Gibson WT, Farooqi IS, Moreau M, DePaoli AM, Lawrence E, O’Rahilly S, Trussell RA (2004). Congenital leptin deficiency due to homozygosity for the Δ133G mutation: report of another case and evaluation of response to four years of leptin therapy. Journal of Clinical Endocrinology and Metabolism.

[ref-112] Gill R, Cheung YH, Shen Y, Lanzano P, Mirza NM, Ten S, Maclaren NK, Motaghedi R, Han JC, Yanovski JA, Leibel RL, Chung WK (2014). Whole-exome sequencing identifies novel LEPR mutations in individuals with severe early onset obesity. Obesity.

[ref-113] Gordon JW, Ruddle FH (1981). Integration and stable germ line transmission of genes injected into mouse pronuclei. Science.

[ref-114] Gray J, Yeo G, Hung C, Keogh J, Clayton P, Banerjee K, McAulay A, O’Rahilly S, Farooqi IS (2006a). Functional characterization of human NTRK2 mutations identified in patients with severe early-onset obesity. International Journal of Obesity (2005).

[ref-115] Gray J, Yeo GSH, Cox JJ, Morton J, Adlam A-L R., Keogh JM, Yanovski JA, El Gharbawy A, Han JC, Tung YCL, Hodges JR, Raymond FL, O’Rahilly S, Farooqi IS (2006b). Hyperphagia severe obesity impaired cognitive function hyperactivity associated with functional loss of one copy of the brain-derived neurotrophic factor (BDNF) gene. Diabetes.

[ref-116] Gunstad J, Schofield P, Paul R, Spitznagel M, Ronald C, Williams LM, Kohn M, Gordon E (2006). BNDF Val66Met polymorphism associated with body mass index in healthy adults. Neuropsychobiology.

[ref-117] Gutierrez-Aguilar R, Kim D-H, Casimir M, Dai X-Q, Pfluger PT, Park J, Haller A, Donelan E, Park J, D’Alessio D, Woods SC, MacDonald PE, Seeley RJ (2014). The role of the transcription factor ETV5 in insulin exocytosis. Diabetologia.

[ref-118] Han JC, Liu Q-R, Jones MaryP., Levinn RL, Menzie CM, Jefferson-George KS, Adler-Wailes DC, Sanford EL, Lacbawan FL, Uhl GR, Rennert OM, Yanovski JA (2008). Brain-derived neurotrophic factor and obesity in the WAGR syndrome. The New England Journal of Medicine.

[ref-119] Harbers K, Jahner D, Jaenisch R (1981). Microinjection of cloned retroviral genomes into mouse zygotes: integration and expression in the animal. Nature.

[ref-120] Harris M, Aschkenasi C, Elias CF, Chandrankunnel A, Nillni EA, Bjørbæk C, Elmquist JK, Flier JS, Hollenberg AN (2001). Transcriptional regulation of the thyrotropin-releasing hormone gene by leptin and melanocortin signaling. The Journal of Clinical Investigation.

[ref-121] Herberg L, Coleman DL (1977). Laboratory animals exhibiting obesity and diabetes syndromes. Metabolism: Clinical and Experimental.

[ref-122] Hill J (1998). Environmental contributions to the obesity epidemic. Science.

[ref-123] Hinney A, Nguyen TT, Scherag A, Friedel S, Brönner G, Müller TD, Grallert H, Illig T, Wichmann H-E, Rief W, Schäfer H, Hebebrand J (2007). Genome wide association (GWA) study for early onset extreme obesity supports the role of fat mass and obesity associated gene (FTO) variants. PLoS ONE.

[ref-124] Hirasawa A, Tsumaya K, Awaji T, Katsuma S, Adachi T, Yamada M, Sugimoto Y, Miyazaki S, Tsujimoto G (2005). Free fatty acids regulate gut incretin glucagon-like peptide-1 secretion through GPR120. Nature Medicine.

[ref-125] Hirschhorn JN, Lohmueller K, Byrne E, Hirschhorn K (2002). A comprehensive review of genetic association studies. Genetics in Medicine: Official Journal of the American College of Medical Genetics.

[ref-126] Holder JL, Butte NF, Zinn AR (2000). Profound obesity associated with a balanced translocation that disrupts the SIM1 gene. Human Molecular Genetics.

[ref-127] Holder L, Zhang L, Kublaoui BM, Dileone RJ, Bair Oz, Lee OK, Zinn CH (2004). Sim1 gene dosage modulates the homeostatic feeding response to increased dietary fat in mice. American Journal of Physiology. Endocrinology and Metabolism.

[ref-128] Horvat S, Bünger L, Falconer VM, Mackay P, Law A, Bulfield G, Keightley PD (2000). Mapping of obesity QTLs in a cross between mouse lines divergently selected on fat content. Mammalian Genome: Official Journal of the International Mammalian Genome Society.

[ref-129] Hu JK, Wang X, Wang P (2014). Testing gene-gene interactions in genome wide association studies. Genetic Epidemiology.

[ref-130] Huang S, Kamihira M (2013). Development of hybrid viral vectors for gene therapy. Biotechnology Advances.

[ref-131] Hummel KP, Dickie MM, Coleman DL (1966). Diabetes, a new mutation in the mouse. Science.

[ref-132] Huszar D, Lynch CA, Fairchild-Huntress V, Dunmore JH, Fang Q, Berkemeier LR, Gu W, Kesterson RA, Boston BA, Cone RD, Smith FJ, Campfield LA, Burn P, Lee F (1997). Targeted disruption of the melanocortin-4 receptor results in obesity in mice. Cell.

[ref-133] Ichimura A, Hirasawa A, Poulain-Godefroy O, Bonnefond A, Hara T, Yengo L, Kimura I, Leloire A, Liu N, Iida K, Choquet H, Besnard P, Lecoeur C, Vivequin S, Ayukawa K, Takeuchi M, Ozawa K, Tauber M, Maffeis C, Morandi A, Buzzetti R, Elliott P, Pouta A, Jarvelin M-R, Körner A, Kiess W, Pigeyre M, Caiazzo R, Van Hul W, Van Gaal L, Horber F, Balkau B, Lévy-Marchal C, Rouskas K, Kouvatsi A, Hebebrand J, Hinney A, Scherag A, Pattou F, Meyre D, Koshimizu Taka-aki, Wolowczuk I, Tsujimoto G, Froguel P (2012). Dysfunction of lipid sensor GPR120 leads to obesity in both mouse and human. Nature.

[ref-134] Igel M, Taylor BA, Phillips SJ, Becker W, Herberg L, Joost HG (1998). Hyperleptinemia and leptin receptor variant Asp600Asn in the obese, hyperinsulinemic KK mouse strain. Journal of Molecular Endocrinology.

[ref-135] Ikeda H (1994). KK Mouse. Diabetes Research and Clinical Practice.

[ref-136] Indo Y, Tsuruta M, Hayashida Y, Karim M, Ohta K, Kawano T, Mitsubuchi H, Tonoki H, Awaya Y, Matsuda I (1996). Mutations in the TRKA/NGF receptor gene in patients with congenital insensitivity to pain with anhidrosis. Nature Genetics.

[ref-137] Jackson RS, Creemers JWM, Ohagi S, Raffin-Sanson M-L, Sanders L, Montague CT, Hutton JC, O’Rahilly S (1997). Obesity and impaired prohormone processing associated with mutations in the human prohormone convertase 1 gene. Nature Genetics.

[ref-138] Jackson RS, Creemers JWM, Farooqi IS, Raffin-Sanson M-L, Varro A, Dockray GJ, Holst JJ, Brubaker PL, Corvol P, Polonsky KS, Ostrega D, Becker KL, Bertagna X, Hutton JC, White A, Dattani MT, Hussain K, Middleton SJ, Nicole TM, Milla PJ, Lindley KJ, O’Rahilly S (2003). Small-intestinal dysfunction accompanies the complex endocrinopathy of human proprotein convertase 1 deficiency. The Journal of Clinical Invesitigation.

[ref-139] Jacquemont S, Reymond A, Zufferey F, Harewood L, Walters RG, Kutalik Z, Martinet D, Shen Y, Valsesia A, Beckmann ND, Thorleifsson G, Belfiore M, Bouquillon S, Campion D, de Leeuw N, de Vries BBA, Esko T, Fernandez BA, Fernández-Aranda F, Fernández-Real JM, Gratacòs M, Guilmatre A, Hoyer J, Jarvelin M-R, Frank Kooy R, Kurg A, Le Caignec C, Männik K, Platt OS, Sanlaville D, Van Haelst MM, Villatoro Gomez S, Walha F, Wu Bai-lin, Yu Y, Aboura A, Addor M-C, Alembik Y, Antonarakis SE, Arveiler B, Barth M, Bednarek N, Béna F, Bergmann S, Beri M, Bernardini L, Blaumeiser B, Bonneau D, Bottani A, Boute O, Brunner HG, Cailley D, Callier P, Chiesa J, Chrast J, Coin L, Coutton C, Cuisset J-M, Cuvellier J-C, David A, de Freminville B, Delobel B, Delrue M-A, Demeer B, Descamps D, Didelot G, Dieterich K, Disciglio V, Doco-Fenzy M, Drunat S, Duban-Bedu B, Dubourg C, El-Sayed Moustafa JS, Elliott P, Faas BHW, Faivre L, Faudet A, Fellmann F, Ferrarini A, Fisher R, Flori E, Forer L, Gaillard D, Gerard M, Gieger C, Gimelli S, Gimelli G, Grabe HJ, Guichet A, Guillin O, Hartikainen A-L, Heron D, Hippolyte L, Holder M, Homuth G, Isidor B, Jaillard S, Jaros Z, Jiménez-Murcia S, Joly Helas G, Jonveaux P, Kaksonen S, Keren B, Kloss-Brandstätter A, Knoers Nine V. A. M., Koolen DA, Kroisel PM, Kronenberg F, Labalme A, Landais E, Lapi E, Layet V, Legallic S, Leheup B, Leube B, Lewis S, Lucas J, MacDermot KD, Magnusson P, Marshall C, Mathieu-Dramard M, McCarthy MI, Meitinger T, Antonietta Mencarelli M, Merla G, Moerman A, Mooser V, Morice-Picard F, Mucciolo M, Nauck M, Coumba Ndiaye N, Nordgren A, Pasquier L, Petit F, Pfundt R, Plessis G, Rajcan-Separovic E, Paolo Ramelli G, Rauch A, Ravazzolo R, Reis A, Renieri A, Richart C, Ried JS, Rieubland C, Roberts W, Roetzer KM, Rooryck C, Rossi M, Saemundsen E, Satre V, Schurmann C, Sigurdsson E, Stavropoulos DJ, Stefansson H, Tengström C, Thorsteinsdóttir U, Tinahones FJ, Touraine R, Vallée L, van Binsbergen E, Van der Aa N, Vincent-Delorme C, Visvikis-Siest S, Vollenweider P, Völzke H, Vulto-van Silfhout AT, Waeber G, Wallgren-Pettersson C, Witwicki RM, Zwolinksi S, Andrieux J, Estivill X, Gusella JF, Gustafsson O, Metspalu A, Scherer SW, Stefansson K, Blakemore AIF, Beckmann JS, Froguel P (2011). Mirror extreme BMI phenotypes associated with gene dosage at the chromosome 16p11.2 locus. Nature.

[ref-140] Jelinek D, Millward V, Birdi A, Trouard TP, Heidenreich RA, Garver WS (2010). Npc1 haploinsufficiency promotes weight gain and metabolic features associated with insulin resistance. Human Molecular Genetics.

[ref-141] Jinek M, Chylinski K, Fonfara I, Hauer M, Doudna JA, Charpentier E (2012). A programmable dual-RNA-guided DNA endonuclease in adaptive bacterial immunity. Science.

[ref-142] Justice MJ (2000). Capitalizing on large-scale mouse mutagenesis screens. Nature Reviews. Genetics.

[ref-143] Justice MJ, Siracusa LD, Stewart AF (2011). Technical approaches for mouse models of human disease. Disease Models and Mechanisms.

[ref-144] Kaiyala KJ, Morton GJ, Leroux BG, Ogimoto K, Wisse B, Schwartz MW (2010). Identification of body fat mass as a major determinant of metabolic rate in mice. Diabetes.

[ref-145] Kanasaki K, Koya D (2011). Biology of obesity: lessons from animal models of obesity. Journal of Biomedicine and Biotechnology.

[ref-146] Kaspar BK, Vissel B, Bengoechea T, Crone S, Randolph-Moore L, Muller R, Brandon EP, Schaffer D, Verma IM, Lee K-F, Heinemann SF, Gage FH (2002). Adeno-associated virus effectively mediates conditional gene modification in the brain. Proceedings of the National Academy of Sciences of the United States of America.

[ref-147] Kernie SG, Liebl DJ, Parada LF (2000). BDNF regulates eating behavior and locomotor activity in mice. The EMBO Journal.

[ref-148] Kirk SFL, Penney TL, McHugh T-LF (2010). Characterizing the obesogenic environment: the state of the evidence with directions for future research. Obesity Reviews: An Official Journal of the International Association for the Study of Obesity.

[ref-149] Kirk SFL, Penney TL, Mchugh T-LF, Sharma AM (2012). Effective weight management practice: a review of the lifestyle intervention evidence. International Journal of Obesity.

[ref-150] Klebig ML, Wilkinson JE, Geisler JG, Woychik RP (1995). Ectopic expression of the agouti gene in transgenic mice causes obesity, features of type II diabetes, and yellow fur. Proceedings of the National Academy of Sciences of the United States of America.

[ref-151] Kleyn PW, Fan W, Kovats SG, Lee JJ, Pulido JC, Wu Y, Berkemeier LR, Misumi DJ, Holmgren L, Charlat O, Woolf EA, Tayber O, Brody T, Shu P, Hawkins F, Kennedy B, Baldini L, Ebeling C, Alperin GD, Deeds J, Lakey ND, Culpepper J, Chen H, Glücksmann-Kuis MA, Carlson GA, Duyk GM, Moore KJ (1996). Identification and characterization of the mouse obesity gene tubby: a member of a novel gene family. Cell.

[ref-152] Kootstra NA, Verma IM (2003). Gene therapy with viral vectors. Annual Review of Pharmacology and Toxicology.

[ref-153] Kosiol C, Vinar T, da Fonseca RR, Hubisz MJ, Bustamante CD, Nielsen R, Siepel A (2008). Patterns of positive selection in six Mammalian genomes. PLoS Genetics.

[ref-154] Koutnikova H, Cock T-A, Watanabe M, Houten SM, Champy M-F, Dierich A, Auwerx J (2003). Compensation by the muscle limits the metabolic consequences of lipodystrophy in PPAR gamma hypomorphic mice. Proceedings of the National Academy of Sciences of the United States of America.

[ref-155] Krude H, Biebermann H, Luck W, Horn R, Brabant G, Grüters A (1998). Severe early-onset obesity, adrenal insufficiency and red hair pigmentation caused by POMC mutations in humans. Nature Genetics.

[ref-156] Krude H, Biebermann H, Schnabel D, Tansek MZ, Theunissen P, Mullis PE, Grüters A (2003). Obesity due to proopiomelanocortin deficiency: three new cases and treatment trials with thyroid hormone and ACTH4-10. The Journal of Clinical Endocrinology and Metabolism.

[ref-157] Kublaoui BM, Holder JL, Gemelli T, Zinn AR (2006). Sim1 haploinsufficiency impairs melanocortin-mediated anorexia and activation of paraventricular nucleus neurons. Molecular Endocrinology.

[ref-158] Kühn R, Torres R, Clarke A (2002). Cre/loxp recombination system and gene targeting. Methods in molecular biology.

[ref-159] Lander ES, Botstein D (1987). Homozygosity mapping: a way to map human recessive traits with the DNA of inbred children. Science.

[ref-160] Laplante M, Horvat S, Festuccia WT, Birsoy K, Prevorsek Z, Efeyan A, Sabatini DM (2012). DEPTOR cell-autonomously promotes adipogenesis and its expression is associated with obesity. Cell Metabolism.

[ref-161] Leduc MS, Lyons M, Darvishi K, Walsh K, Sheehan S, Amend S, Cox A, Orho-Melander M, Kathiresan S, Paigen B, Korstanje R (2011). The mouse QTL map helps interpret human genome-wide association studies for HDL cholesterol. Journal of Lipid Research.

[ref-162] Lee AWS, Cox RD (2011). Use of mouse models in studying type 2 diabetes mellitus. Expert Reviews in Molecular Medicine.

[ref-163] Lee AWS, Hengstler H, Schwald K, Berriel-Diaz M, Loreth D, Kirsch M, Kretz O, Haas CA, de Angelis MH, Herzig S, Brümmendorf T, Klingenspor M, Rathjen FG, Rozman J, Nicholson G, Cox RD, Schäfer MKE (2012). Functional inactivation of the genome-wide association study obesity gene neuronal growth regulator 1 in mice causes a body mass phenotype. PLoS ONE.

[ref-164] Lee YS, Challis BG, Thompson DA, Yeo GSH, Keogh JM, Madonna ME, Wraight V, Sims M, Vatin V, Meyre D, Shield J, Burren C, Ibrahim Z, Cheetham T, Swift P, Blackwood A, Hung Chiao-Chien C., Wareham NJ, Froguel P, Millhauser GL, O’Rahilly S, Farooqi IS (2006). A POMC variant implicates beta-melanocyte-stimulating hormone in the control of human energy balance. Cell Metabolism.

[ref-165] Lentz TB, Gray SJ, Samulski RJ (2012). Viral vectors for gene delivery to the central nervous system. Neurobiology of Disease.

[ref-166] Li A, Meyre D (2013). Challenges in reproducibility of genetic association studies: lessons learned from the obesity field. International Journal of Obesity (2005).

[ref-167] Li A, Meyre D (2014). Jumping on the train of personalized medicine: a primer for non-geneticist clinicians: Part 2. Fundamental concepts in genetic epidemiology. Current Psychiatry Reviews.

[ref-168] Li P, Tiwari HK, Lin W-Y, Allison DB, Chung WK, Leibel RL, Yi N, Liu N (2014). Genetic association analysis of 30 genes related to obesity in a European American population. International Journal of Obesity.

[ref-169] Li S, Zhao JH, Luan Jian’an, Ekelund U, Luben RN, Khaw K-T, Wareham NJ, Loos RJF (2010). Physical activity attenuates the genetic predisposition to obesity in 20,000 men and women from EPIC-Norfolk prospective population study. PLoS Medicine.

[ref-170] Liang J, Fu M, Ciociola E, Chandalia M, Abate N (2007). Role of ENPP1 on adipocyte maturation. PLoS ONE.

[ref-171] Lin Y, Cradick TJ, Brown MT, Deshmukh H, Ranjan P, Sarode N, Wile BM, Vertino PM, Stewart FJ, Bao G (2014). CRISPR/Cas9 systems have off-target activity with insertions or deletions between target DNA guide RNA sequences. Nucleic Acids Research.

[ref-172] Lloyd DJ, Bohan S, Gekakis N (2006). Obesity, hyperphagia and increased metabolic efficiency in Pc1 mutant mice. Human Molecular Genetics.

[ref-173] Locke AE, Kahali B, Berndt SI, Justice AE, Pers TH, Day FR, Powell C, Vedantam S, Buchkovich ML, Yang J, Croteau-Chonka DC, Esko T, Fall T, Ferreira T, Gustafsson S, Kutalik Z, Luan J, Mägi R, Randall JC, Winkler TW, Wood AR, Workalemahu T, Faul JD, Smith JA, Hua Zhao J, Zhao W, Chen J, Fehrmann R, Hedman ÅK, Karjalainen J, Schmidt EM, Absher D, Amin N, Anderson D, Beekman M, Bolton JL, Bragg-Gresham JL, Buyske S, Demirkan A, Deng G, Ehret GB, Feenstra B, Feitosa MF, Fischer K, Goel A, Gong J, Jackson AU, Kanoni S, Kleber ME, Kristiansson K, Lim U, Lotay V, Mangino M, Mateo Leach I, Medina-Gomez C, Medland SE, Nalls MA, Palmer CD, Pasko D, Pechlivanis S, Peters MJ, Prokopenko I, Shungin D, Stanáková A, Strawbridge RJ, Ju Sung Y, Tanaka T, Teumer A, Trompet S, van der Laan SW, van Setten J, Van Vliet-Ostaptchouk JV, Wang Z, Yengo L, Zhang W, Isaacs A, Albrecht E, Ärnlöv J, Arscott GM, Attwood AP, Bandinelli S, Barrett A, Bas IN, Bellis C, Bennett AJ, Berne C, Blagieva R, Blüher M, Böhringer S, Bonnycastle LL, Böttcher Y, Boyd HA, Bruinenberg M, Caspersen IH, Ida Chen Y-D, Clarke R, Warwick Daw E, de Craen AJM, Delgado G, Dimitriou M, Doney ASF, Eklund N, Estrada K, Eury E, Folkersen L, Fraser RM, Garcia ME, Geller F, Giedraitis V, Gigante B, Go AS, Golay A, Goodall AH, Gordon SD, Gorski M, Grabe HJ, Grallert H, Grammer TB, Gräßler J, Grönberg H, Groves CJ, Gusto G, Haessler J, Hall P, Haller T, Hallmans G, Hartman CA, Hassinen M, Hayward C, Heard-Costa NL, Helmer Q, Hengstenberg C, Holmen O, Hottenga J-J, James AL, Jeff JM, Johansson Åsa, Jolley J, Juliusdottir T, Kinnunen L, Koenig W, Koskenvuo M, Kratzer W, Laitinen J, Lamina C, Leander K, Lee NR, Lichtner P, Lind L, Lindström J, Sin Lo K, Lobbens S, Lorbeer R, Lu Y, Mach F, Magnusson PKE, Mahajan A, McArdle WL, McLachlan S, Menni C, Merger S, Mihailov E, Milani L, Moayyeri A, Monda KL, Morken MA, Mulas A, Müller G, Müller-Nurasyid M, Musk AW, Nagaraja R, Nöthen MM, Nolte IM, Pilz S, Rayner NW, Renstrom F, Rettig R, Ried JS, Ripke S, Robertson NR, Rose LM, Sanna S, Scharnagl H, Scholtens S, Schumacher FR, Scott WR, Seufferlein T, Shi J, Vernon Smith A, Smolonska J, Stanton AV, Steinthorsdottir V, Stirrups K, Stringham HM, Sundström J, Swertz MA, Swift AJ, Syvänen A-C, Tan S-T, Tayo BO, Thorand B, Thorleifsson G, Tyrer JP, Uh H-W, Vandenput L, Verhulst FC, Vermeulen SH, Verweij N, Vonk JM, Waite LL, Warren HR, Waterworth D, Weedon MN, Wilkens LR, Willenborg C, Wilsgaard T, Wojczynski MK, Wong A, Wright AF, Zhang Q, Brennan EP, Choi M, Dastani Z, Drong AW, Eriksson P, Franco-Cereceda A, Gådin JR, Gharavi AG, Goddard ME, Handsaker RE, Huang J, Karpe F, Kathiresan S, Keildson S, Kiryluk K, Kubo M, Lee J-Y, Liang L, Lifton RP, Ma B, McCarroll SA, McKnight AJ, Min JL, Moffatt MF, Montgomery GW, Murabito JM, Nicholson G, Nyholt DR, Okada Y, Perry JRB, Dorajoo R, Reinmaa E, Salem RM, Sandholm N, Scott RA, Stolk L, Takahashi A, Tanaka T, van’t Hooft FM, Vinkhuyzen AAE, Westra H-J, Zheng W, Zondervan KT, Heath AC, Arveiler D, Bakker SJL, Beilby J, Bergman RN, Blangero J, Bovet P, Campbell H, Caulfield MJ, Cesana G, Chakravarti A, Chasman DI, Chines PS, Collins FS, Crawford DC, Adrienne Cupples L, Cusi D, Danesh J, de Faire U, den Ruijter HM, Dominiczak AF, Erbel R, Erdmann J, Eriksson JG, Farrall M, Felix SB, Ferrannini E, Ferrières J, Ford I, Forouhi NG, Forrester T, Franco OH, Gansevoort RT, Gejman PV, Gieger C, Gottesman O, Gudnason V, Gyllensten U, Hall AS, Harris TB, Hattersley AT, Hicks AA, Hindorff LA, Hingorani AD, Hofman A, Homuth G, Kees Hovingh G, Humphries SE, Hunt SC, Hyppönen E, Illig T, Jacobs KB, Jarvelin M-R, Jöckel K-H, Johansen B, Jousilahti P, Wouter Jukema J, Jula AM, Kaprio J, Kastelein JJP, Keinanen-Kiukaanniemi SM, Kiemeney LA, Knekt P, Kooner JS, Kooperberg C, Kovacs P, Kraja AT, Kumari M, Kuusisto J, Lakka TA, Langenberg C, Le Marchand L, Lehtimäki T, Lyssenko V, Männistö S, Marette A, Matise TC, McKenzie CA, McKnight B, Moll FL, Morris AD, Morris AP, Murray JC, Nelis M, Ohlsson C, Oldehinkel AJ, Ong KK, Madden PAF, Pasterkamp G, Peden JF, Peters A, Postma DS, Pramstaller PP, Price JF, Qi L, Raitakari OT, Rankinen T, Rao DC, Rice TK, Ridker PM, Rioux JD, Ritchie MD, Rudan I, Salomaa V, Samani NJ, Saramies J, Sarzynski MA, Schunkert H, Schwarz PEH, Sever P, Shuldiner AR, Sinisalo J, Stolk RP, Strauch K, Tönjes A, Trégouët D-A, Tremblay A, Tremoli E, Virtamo J, Vohl M-C, Völker U, Waeber G, Willemsen G, Witteman JC, Carola Zillikens M, Adair LS, Amouyel P, Asselbergs FW, Assimes TL, Bochud M, Boehm BO, Boerwinkle E, Bornstein SR, Bottinger EP, Bouchard C, Cauchi S, Chambers JC, Chanock SJ, Cooper RS, de Bakker PIW, Dedoussis G, Ferrucci L, Franks PW, Froguel P, Groop LC, Haiman CA, Hamsten A, Hui J, Hunter DJ, Hveem K, Kaplan RC, Kivimaki M, Kuh D, Laakso M, Liu Y, Martin NG, März W, Melbye M, Metspalu A, Moebus S, Munroe PB, Njølstad I, Oostra BA, Palmer CNA, Pedersen NL, Perola M, Pérusse L, Peters U, Power C, Quertermous T, Rauramaa R, Rivadeneira F, Saaristo TE, Saleheen D, Sattar N, Schadt EE, Schlessinger D, Eline Slagboom P, Snieder H, Spector TD, Thorsteinsdottir U, Stumvoll M, Tuomilehto J, Uitterlinden AG, Uusitupa M, van der Harst P, Walker M, Wallaschofski H, Wareham NJ, Watkins H, Weir DR, Wichmann H-E, Wilson JF, Zanen P, Borecki IB, Deloukas P, Fox CS, Heid IM, O’Connell JR, Strachan DP, Stefansson K, van Duijn CM, Abecasis GR, Franke L, Frayling TM, McCarthy MI, Visscher PM, Scherag A, Willer CJ, Boehnke M, Mohlke KL, Lindgren CM, Beckmann JS, Barroso I, North KE, Ingelsson E, Hirschhorn JN, Loos RJF, Speliotes EK (2015). Genetic studies of body mass index yield new insights for obesity biology. Nature.

[ref-174] Loebel DAF, Hor ACC, Bildsoe HK, Tam PPL (2014). Timed deletion of twist1 in the limb bud reveals age-specific impacts on autopod and zeugopod patterning. PLoS ONE.

[ref-175] Luan B, Zhao J, Wu H, Duan B, Shu G, Wang X, Li D, Jia W, Kang J, Pei G (2009). Deficiency of a beta-arrestin-2 signal complex contributes to insulin resistance. Nature.

[ref-176] MacKay T (2014). Epistasis and quantitative traits: using model organisms to study gene-gene interactions. Nature Reviews Genetics.

[ref-177] Mackay TFC, Stone EA, Ayroles JF (2009). The genetics of quantitative traits: challenges and prospects. Nature Reviews. Genetics.

[ref-178] Maddux BA, Chang Y, Accili D, Mcguinness OP, Youngren JF, Goldfine ID, Over IDG (2006). Overexpression of the insulin receptor inhibitor PC-1/ENPP1 induces insulin resistance and hyperglycemia. American Journal of Physiology, Endocrinology and Metabolism.

[ref-179] Maddux BA, Goldfin ID (2000). Membrane glycoprotein PC-1 inhibition of insulin receptor function occurs via direct interaction with the receptor alpha-subunit. Diabetes.

[ref-180] Maddux BA, Sbraccia P, Kumakura S, Sasson S, Youngren J, Fisher A, Spencer S, Grupe A, Henzel W, Stewart TA, Reaven GM, Goldfine ID (1995). Membrane glycoprotein PC-1 and insulin resistance in non-insulin dependent diabetes mellitus. Nature.

[ref-181] Marg A, Sirim P, Spaltmann F, Plagge A, Kauselmann G, Buck F, Rathjen FG, Brümmendorf T (1999). Neurotractin, a novel neurite outgrowth-promoting Ig-like protein that interacts with CEPU-1 and LAMP. Journal of Cell Biology.

[ref-182] Martín MG, Lindberg I, Solorzano–Vargas RS, Wang J, Avitzur Y, Bandsma R, Sokollik C, Lawrence S, Pickett LA, Chen Z, Egritas O, Dalgic B, Albornoz V, de Ridder L, Hulst J, Gok F, Aydoan A, Al–Hussaini A, Gok DE, Yourshaw M, Wu SV, Cortina G, Stanford S, Georgia S (2013). Congenital proprotein convertase 1/3 deficiency causes malabsorptive diarrhea and other endocrinopathies in a pediatric cohort. Gastroenterology.

[ref-183] May C, Rivella S, Callegari J, Heller G, Gaensler KM, Luzzatto L, Sadelain M (2000). Therapeutic haemoglobin synthesis in beta-thalassaemic mice expressing lentivirus-encoded human beta-globin. Nature.

[ref-184] Mazen I, El-Gammal M, Abdel-Hamid M, Amr K (2009). A novel homozygous missense mutation of the leptin gene (N103K) in an obese Egyptian patient. Molecular Genetics and Metabolism.

[ref-185] McClurg P, Janes J, Wu C, Delano DL, Walker JR, Batalov S, Takahashi JS, Shimomura K, Kohsaka A, Bass J, Wiltshire T, Su AI (2007). Genomewide association analysis in diverse inbred mice: power and population structure. Genetics.

[ref-186] McMurray F, Church CD, Larder R, Nicholson G, Wells S, Teboul L, Tung YCL, Rimmington D, Bosch F, Jimenez V, Yeo GSH, O’Rahilly S, Ashcroft FM, Coll AP, Cox RD (2013). Adult onset global loss of the fto gene alters body composition and metabolism in the mouse. PLoS Genetics.

[ref-187] Mencarelli M, Dubern B, Alili R, Maestrini S, Benajiba L, Tagliaferri M, Galan P, Rinaldi M, Simon C, Tounian P, Hercberg S, Liuzzi A, Di Blasio AM, Clement K (2011). Rare melanocortin-3 receptor mutations with in vitro functional consequences are associated with human obesity. Human Molecular Genetics.

[ref-188] Mendiratta MS, Yang Y, Balazs AE, Willis AS, Eng CM, Karaviti LP, Potocki L (2011). Early onset obesity and adrenal insufficiency associated with a homozygous POMC mutation. International Journal of Pediatric Endocrinology.

[ref-189] Menke DB (2013). Engineering subtle targeted mutations into the mouse genome. Genesis.

[ref-190] Meyre D, Bouatia-Naji N, Tounian A, Samson C, Lecoeur C, Vatin V, Ghoussaini M, Wachter C, Hercberg S, Charpentier G, Patsch W, Pattou F, Charles M-A, Tounian P, Clément K, Jouret B, Weill J, Maddux BA, Goldfine ID, Walley A, Boutin P, Dina C, Froguel P (2005). Variants of ENPP1 are associated with childhood and adult obesity and increase the risk of glucose intolerance and type 2 diabetes. Nature Genetics.

[ref-191] Meyre D, Delplanque J, Chèvre J-C, Lecoeur C, Lobbens S, Gallina S, Durand E, Vatin V, Degraeve F, Proença C, Gaget S, Körner A, Kovacs P, Kiess W, Tichet J, Marre M, Hartikainen A-L, Horber F, Potoczna N, Hercberg S, Levy-Marchal C, Pattou F, Heude B, Tauber M, McCarthy MI, Blakemore AIF, Montpetit A, Polychronakos C, Weill J, Coin LJM, Asher J, Elliott P, Järvelin M-R, Visvikis-Siest S, Balkau B, Sladek R, Balding D, Walley A, Dina C, Froguel P (2009). Genome-wide association study for early-onset and morbid adult obesity identifies three new risk loci in European populations. Nature Genetics.

[ref-192] Meyre D, Farge M, Lecoeur C, Proenca C, Durand E, Allegaert F, Tichet J, Marre M, Balkau B, Weill J, Delplanque J, Froguel P (2008). R125W coding variant in TBC1D1 confers risk for familial obesity contributes to linkage on chromosome 4p14 in the French population. Human Molecular Genetics.

[ref-193] Meyre D, Lecoeur C, Delplanque J, Francke S, Vatin V, Durand E, Weill J, Dina C, Froguel P (2004). A genome-wide scan for childhood obesity-associated traits in French families shows significant linkage on chromosome 6q22.31-q23.2. Diabetes.

[ref-194] Michaud JL, Boucher F, Melnyk A, Gauthier F, Goshu E, Lévy E, Mitchell GA, Himms-Hagen J, Fan CM (2001). Sim1 haploinsufficiency causes hyperphagia obesity reduction of the paraventricular nucleus of the hypothalamus. Human Molecular Genetics.

[ref-195] Michaud JL, DeRossi C, May NR, Holdener BC, Fan CM (2000). ARNT2 acts as the dimerization partner of SIM1 for the development of the hypothalamus. Mechanisms of Development.

[ref-196] Michaud JL, Rosenquist T, May NR, Fan CM (1998). Development of neuroendocrine lineages requires the bHLH-PAS transcription factor SIM1. Genes and Development.

[ref-197] Miller MW, Duhl DM, Vrieling H, Cordes SP, Ollmann MM, Winkes BM, Barsh GS (1993). Cloning of the mouse agouti gene predicts a secreted protein ubiquitously expressed in mice carrying the lethal yellow mutation. Genes and Development.

[ref-198] Misra A, Khurana L (2008). Obesity and the metabolic syndrome in developing countries. The Journal of Clinical Endocrinology and Metabolism.

[ref-199] Mollah MBR, Ishikawa A (2011). Intersubspecific subcongenic mouse strain analysis reveals closely linked QTLs with opposite effects on body weight. Mammalian Genome: Official Journal of the International Mammalian Genome Society.

[ref-200] Montague CT, Farooqi IS, Whitehead JP, Soos MA, Rau H, Wareham NJ, Sewter CP, Digby JE, Mohammed SN, Hurst JA, Cheetham CH, Earley AR, Barnett AH, Prins JB, O’Rahilly S (1997). Congenital leptin deficiency is associated with severe early-onset obesity in humans. Nature.

[ref-201] Monteggia LM, Barrot M, Powell CM, Berton O, Galanis V, Gemelli T, Meuth S, Nagy A, Greene RW, Nestler EJ (2004). Essential role of brain-derived neurotrophic factor in adult hippocampal function. Proceedings of the National Academy of Sciences of the United States of America.

[ref-202] Morrison AC, Voorman A, Johnson AD, Liu X, Yu J, Li A, Muzny D, Yu F, Rice K, Zhu C, Bis J, Heiss G, O’Donnell CJ, Psaty BM, Cupples LA, Gibbs R, Boerwinkle E (2013). Whole-genome sequence-based analysis of high-density lipoprotein cholesterol. Nature Genetics.

[ref-203] Must A, Spadano J, Coakley EH, Field AE, Colditz G, Dietz WH (1999). The disease burden associated with overweight and obesity. JAMA: The Journal of the American Medical Association.

[ref-204] Nadeau JH, Singer JB, Matin A, Lander ES (2000). Analysing complex genetic traits with chromosome substitution strains. Nature.

[ref-205] Naggert JK, Fricker LD, Varlamov O, Nishina PM, Rouille Y, Steiner DF, Carroll RJ, Paigen BJ, Leiter EH (1995). Hyperproinsulinaemia in obese fat/fat mice associated with a carboxypeptidase E mutation which reduses enzyme activity. Nature Genetics.

[ref-206] Noble EE, Billington CJ, Kotz CM, Wang C (2011). The lighter side of BDNF. American Journal of Physiology—Regulatory, Integrative and Comparative Physiology.

[ref-207] O’Kane C, Gehrin W (1987). Detection *in situ* of genomic regulatory elements in Drosophila. Proceedings of the National Academy of Sciences of the United States of America.

[ref-208] O’Rahilly S, Farooqi IS (2008). Human obesity: a heritable neurobehavioral disorder that is highly sensitive to environmental conditions. Diabetes.

[ref-209] Okazaki M, Saito Y, Udaka Y, Maruyama M, Murakami H, Ota S, Kikuchi T, Oguchi K (2002). Diabetic nephrophathy in KK and KK-Ay mice. Experimental Animals.

[ref-210] Olshansky SJ, Passaro DJ, Hershow RC, Layden J, Carnes BA, Brody J, Hayflick L, Butler RN, Allison DB, Ludwig DS (2005). A potential decline in life expectancy in the United States in the 21st century. The New England Journal of Medicine.

[ref-211] Osten P, Dittgen T, Licznerski P (2006). Lentivirus-based genetic manipulations in neurons *in vivo*. The dynamic synapse: molecular methods in ionotropic receptor biology.

[ref-212] Otto EA, Hurd TW, Airik R, Chaki M, Zhou W, Stoetzel C, Patil SB, Levy S, Ghosh AK, Murga-Zamalloa CA, van Reeuwijk J, Letteboer SJF, Sang L, Giles RH, Liu Q, Coene KLM, Estrada-Cuzcano A, Collin RWJ, McLaughlin HM, Held S, Kasanuki JM, Ramaswami G, Conte J, Lopez I, Washburn J, MacDonald J, Hu J, Yamashita Y, Maher ER, Guay-Woodford LM, Neumann HPH, Obermüller N, Koenekoop RK, Bergmann C, Bei X, Lewis RA, Katsanis N, Lopes V, Williams DS, Lyons RH, Dang CV, Brito DA, Dias MB, Zhang X, Cavalcoli JD, Nürnberg G, Nürnberg P, Pierce EA, Jackson PK, Antignac C, Saunier S, Roepman R, Dollfus H, Khanna H, Hildebrandt F (2010). Candidate exome capture identifies mutation of SDCCAG8 as the cause of a retinal-renal ciliopathy. Nature Genetics.

[ref-213] Paigen K (2003). One hundred years of mouse genetics: an intellectual history. I. The classical period (1902–1980). Genetics.

[ref-214] Parks BW, Nam E, Org E, Kostem E, Norheim F, Hui ST, Pan C, Civelek M, Rau CD, Bennett BJ, Mehrabian M, Ursell LK, He A, Castellani LW, Zinker B, Kirby M, Drake TA, Drevon CA, Knight R, Gargalovic P, Kirchgessner T, Eskin E, Lusis AJ (2013). Genetic control of obesity and gut microbiota composition in response to high-fat, high-sucrose diet in mice. Cell Metabolism.

[ref-215] Patwari P, Emilsson V, Schadt EE, Chutkow WA, Lee S, Marsili A, Zhang Y, Dobrin R, Cohen DE, Larsen PR, Zavacki AM, Fong LG, Young SG, Lee RT (2011). The arrestin domain-containing 3 protein regulates body mass and energy expenditure. Cell Metabolism.

[ref-216] Pearce LR, Atanassova N, Banton MC, Bottomley B, Van der Klaauw AA, Revelli J-P, Hendricks A, Keogh JM, Henning E, Doree D, Jeter-Jones S, Garg S, Bochukova EG, Bounds R, Ashford S, Gayton E, Hindmarsh PC, Shield JPH, Crowne E, Barford D, Wareham NJ, O’Rahilly S, Murphy MP, Powell DR, Barroso I, Farooqi IS, UK10K consortium (2013). KSR2 mutations are associated with obesity, insulin resistance, and impaired cellular fuel oxidation. Cell.

[ref-217] Pearce LR, Joe R, Doche ME, Su H-W, Keogh JM, Henning E, Argetsinger LS, Bochukova EG, Cline JM, Garg S, Saeed S, Shoelson S, O’Rahilly S, Barroso I, Rui L, Farooqi IS, Carter-Su C (2014). Functional characterisation of obesity-associated variants involving the alpha and beta isoforms of human SH2B1. Endocrinology.

[ref-218] Philip VM, Sokoloff G, Ackert-Bicknell CL, Striz M, Branstetter L, Beckmann MA, Spence JS, Jackson BL, Galloway LD, Barker P, Wymore AM, Hunsicker PR, Durtschi DC, Shaw GS, Shinpock S, Manly KF, Miller DR, Donohue KD, Culiat CT, Churchill GA, Lariviere WR, Palmer AA, O’Hara BF, Voy BH, Chesler EJ (2011). Genetic analysis in the Collaborative Cross breeding population. Genome Research.

[ref-219] Philippe J, Stijnen P, Meyre D, De Graeve F, Thuillier D, Delplanque J, Gyapay G, Sand O, Creemers JW, Froguel P, Bonnefond A (2014). A nonsense loss-of-function mutation in PCSK1 contributes to dominantly inherited human obesity. International Journal of Obesity.

[ref-220] Pomp D (2007). Natural polygenic models. Obesity: genomics and postgenomics.

[ref-221] Prada PO, Quaresma PGF, Caricilli AM, Santos AC, Guadagnini D, Morari J, Weissmann L, Ropelle ER, Carvalheira JBC, Velloso LA, Saad MJA (2013). Tub has a key role in insulin and leptin signaling and action in vivo in hypothalamic nuclei. Diabetes.

[ref-222] Prevorsek Z, Gorjanc G, Paigen B, Horvat S (2010). Congenic and bioinformatics analyses resolved a major-effect Fob3b QTL on mouse Chr 15 into two closely linked loci. Mammalian Genome: Official Journal of the International Mammalian Genome Society.

[ref-223] Rahmouni K, Fath MA, Seo S, Thedens DR, Berry CJ, Weiss R, Nishimura DY, Sheffield VC (2008). Leptin resistance contributes to obesity and hypertension in mouse models of Bardet-Biedl syndrome. The Journal of Clinical Invesitigation.

[ref-224] Ramachandrappa S, Raimondo A, Cali AMG, Keogh JM, Henning E, Saeed S, Thompson A, Garg S, Bochukova EG, Brage S, Trowse V, Wheeler E, Sullivan AE, Dattani M, Clayton PE, Datta V, Bruning JB, Wareham NJ, O’Rahilly S, Peet DJ, Barroso I, Whitelaw ML, Farooqi IS (2013). Rare variants in single-minded 1 (SIM1) are associated with severe obesity. The Journal of Clinical Invesitigation.

[ref-225] Rankinen T, Zuberi A, Chagnon YC, Weisnagel SJ, Argyropoulos G, Walts B, Pérusse L, Bouchard C (2006). The human obesity gene map: the 2005 update. Obesity.

[ref-226] Reed DR, Lawler MP, Tordoff MG (2008). Reduced body weight is a common effect of gene knockout in mice. BMC Genetics.

[ref-227] Rees DA, Alcolado JC (2005). Animal models of diabetes mellitus. Diabetic Medicine: A Journal of the British Diabetic Association.

[ref-228] Ren D, Li M, Duan C, Rui L (2005). Identification of SH2-B as a key regulator of leptin sensitivity, energy balance, and body weight in mice. Cell Metabolism.

[ref-229] Revelli J-P, Smith D, Allen J, Jeter-Jones S, Shadoan MK, Desai U, Schneider M, Van Sligtenhorst I, Kirkpatrick L, Platt KA, Suwanichkul A, Savelieva K, Gerhardt B, Mitchell J, Syrewicz J, Zambrowicz B, Hamman BD, Vogel P, Powell DR (2011). Profound obesity secondary to hyperphagia in mice lacking kinase suppressor of ras 2. Obesity.

[ref-230] Rios M, Fan G, Fekete C, Kelly J, Bates B, Kuehn R, Lechan RM, Jaenisch R (2001). Conditional deletion of brain-derived neurotrophic factor in the postnatal brain leads to obesity hyperactivity. Molecular Endocrinology.

[ref-231] Rogner UC, Avner P (2003). Congenic mice: cutting tools for complex immune disorders. Nature Reviews. Immunology.

[ref-232] Roselli-Rehfuss KG, Mountjoy LS, Robbins MT, Mortrud MJ, Low JB, Tatro ML, Entwistle RB, Simerly RD, Cone (1993). Identification of a receptor for gamma melanotropin other proopiomelanocortin peptides in the hypothalamus limbic system. Proceedings of the National Academy of Sciences of the United States of America.

[ref-233] Rosenthal N, Brown S (2007). The mouse ascending: perspectives for human-disease models. Nature Cell Biology.

[ref-234] Rouskas K, Meyre D, Stutzmann F, Paletas K, Papazoglou D, Vatin V, Marchand M, Kouvatsi A, Froguel P (2012). Loss-of-function mutations in MC4R are very rare in the Greek severely obese adult population. Obesity.

[ref-235] Russell LB, Hunsicker PR, Cacheiro NL, Bangham JW, Russell WL, Shelby MD (1989). Chlorambucil effectively induces deletion mutations in mouse germ cells. Proceedings of the National Academy of Sciences of the United States of America.

[ref-236] Saeed S, Butt TA, Anwer M, Arslan M, Froguel P (2012). High prevalence of leptin and melanocortin-4 receptor gene mutations in children with severe obesity from Pakistani consanguineous families. Molecular Genetics and Metabolism.

[ref-237] Sano H, Kane S, Sano E, Miinea CP, Asara JM, Lane WS, Garner CW, Lienhard GE (2003). Insulin-stimulated phosphorylation of a Rab GTPase-activating protein regulates GLUT4 translocation. The Journal of Biological Chemistry.

[ref-238] Scheidecker S, Etard C, Pierce NW, Geoffroy V, Schaefer E, Muller J, Chennen K, Flori E, Pelletier V, Poch O, Marion V, Stoetzel C, Strahle U, Nachury MV, Dollfus H (2014). Exome sequencing of Bardet-Biedl syndrome patient identifies a null mutation in the BBSome subunit BBIP1 (BBS18). Journal of Medical Genetics.

[ref-239] Scherag A, Dina C, Hinney A, Vatin V, Scherag S, Vogel CIG, Müller TD, Grallert H, Wichmann H-E, Balkau B, Heude B, Jarvelin M-R, Hartikainen A-L, Levy-Marchal C, Weill J, Delplanque J, Körner A, Kiess W, Kovacs P, Rayner NW, Prokopenko I, McCarthy MI, Schäfer H, Jarick I, Boeing H, Fisher E, Reinehr T, Heinrich J, Rzehak P, Berdel D, Borte M, Biebermann H, Krude H, Rosskopf D, Rimmbach C, Rief W, Fromme T, Klingenspor M, Schürmann A, Schulz N, Nöthen MM, Mühleisen TW, Erbel R, Jöckel K-H, Moebus S, Boes T, Illig T, Froguel P, Hebebrand J, Meyre D (2010). Two new Loci for body-weight regulation identified in a joint analysis of genome-wide association studies for early-onset extreme obesity in French and german study groups. PLoS Genetics.

[ref-240] Scuteri A, Sanna S, Chen W-M, Uda M, Albai G, Strait J, Najjar S, Nagaraja R, Orrú M, Usala G, Dei M, Lai S, Maschio A, Busonero F, Mulas A, Ehret GB, Fink AA, Weder AB, Cooper RS, Galan P, Chakravarti A, Schlessinger D, Cao A, Lakatta E, Abecasis GR (2007). Genome-wide association scan shows genetic variants in the FTO gene are associated with obesity-related traits. PLoS Genetics.

[ref-241] Sementchenko VI, Watson DK (2000). Ets target genes: past, present and future. Oncogene.

[ref-242] Seppen J, Barry SC, Harder B, Osborne WR (2001). Lentivirus administration to rat muscle provides efficient sustained expression of erythropoietin. Blood.

[ref-243] Shao H, Burrage LC, Sinasac DS, Hill AE, Ernest SR, O’Brien W, Courtland H-W, Jepsen KJ, Kirby A, Kulbokas EJ, Daly MJ, Broman KW, Lander ES, Nadeau JH (2008). Genetic architecture of complex traits: large phenotypic effects pervasive epistasis. Proceedings of the National Academy of Sciences of the United States of America.

[ref-244] Sharrocks AD (2001). The ETS-domain transcription factor family. Nature Reviews: Molecular Cell Biology.

[ref-245] Sheffield V (2010). The blind leading the obese: the molecular pathophysiology of a human obesity syndrome. Transactions of the American Clinical and Climatological Association.

[ref-246] Shendure J (2011). Next-generation human genetics. Genome Biology.

[ref-247] Silver L (1995). Mouse genetics: concepts and applications.

[ref-248] Singer J, Hill A, Burrage L, Olszens K, Song J, Justice M, Nadeau J (2004). Genetic dissection of complex traits with chromosome substitution strains of mice. Science.

[ref-249] Singleton A (2011). Exome sequencing: a transformative technology. The Lancet Neurology.

[ref-250] Smemo S, Tena JJ, Kim K-H, Gamazon ER, Sakabe NJ, Gómez-Marín C, Aneas I, Credidio FL, Sobreira DR, Wasserman NF, Lee JH, Puviindran V, Tam D, Shen M, Son JE, Vakili NA, Sung H-K, Naranjo S, Acemel RD, Manzanares M, Nagy A, Cox NJ, Hui C-C, Gomez-Skarmeta JL, Nóbrega MA (2014). Obesity-associated variants within FTO form long-range functional connections with IRX3. Nature.

[ref-251] Smith A, Funder J (1988). Proopiomelanocortin processing in the pituitary, central nervous system, and peripheral tissues. Endocrine Reviews.

[ref-252] Sonestedt E, Lyssenko V, Ericson U, Gullberg B, Wirfält E, Groop L, Orho-Melander M (2012). Genetic variation in the glucose-dependent insulinotropic polypeptide receptor modifies the association between carbohydrate and fat intake and risk of type 2 diabetes in the Malmo Diet and Cancer cohort. The Journal of Clinical Endocrinology and Metabolism.

[ref-253] Speakman J, Hambly C, Mitchell S, Król E (2007). Animal models of obesity. Obesity Reviews: An Official Journal of the International Association for the Study of Obesity.

[ref-254] Speliotes EK, Willer CJ, Berndt SI, Monda KL, Thorleifsson G, Jackson AU, Allen HL, Lindgren CM, Luan Jian’an, Mägi R, Randall JC, Vedantam S, Winkler TW, Qi L, Workalemahu T, Heid IM, Steinthorsdottir V, Stringham HM, Weedon MN, Wheeler E, Wood AR, Ferreira T, Weyant RJ, Segrè AV, Estrada K, Liang L, Nemesh J, Park J-H, Gustafsson S, Kilpeläinen TO, Yang J, Bouatia-Naji N, Esko T, Feitosa MF, Kutalik Z, Mangino M, Raychaudhuri S, Scherag A, Smith AV, Welch R, Zhao JH, Aben KK, Absher DM, Amin N, Dixon AL, Fisher E, Glazer NL, Goddard ME, Heard-Costa NL, Hoesel V, Hottenga J-J, Johansson Å, Johnson T, Ketkar S, Lamina C, Li S, Moffatt MF, Myers RH, Narisu N, Perry JRB, Peters MJ, Preuss M, Ripatti S, Rivadeneira F, Sandholt C, Scott LJ, Timpson NJ, Tyrer JP, van Wingerden S, Watanabe RM, White CC, Wiklund F, Barlassina C, Chasman DI, Cooper MN, Jansson J-O, Lawrence RW, Pellikka N, Prokopenko I, Shi J, Thiering E, Alavere H, Alibrandi MTS, Almgren P, Arnold AM, Aspelund T, Atwood LD, Balkau B, Balmforth AJ, Bennett AJ, Ben-Shlomo Y, Bergman RN, Bergmann S, Biebermann H, Blakemore AIF, Boes T, Bonnycastle LL, Bornstein SR, Brown MJ, Buchanan TA, Busonero F, Campbell H, Cappuccio FP, Cavalcanti-Proença C, Chen Y-DI, Chen C-M, Chines PS, Clarke R, Coin L, Connell J, Day INM, Heijer Martin den, Duan J, Ebrahim S, Elliott P, Elosua R, Eiriksdottir G, Erdos MR, Eriksson JG, Facheris MF, Felix SB, Fischer-Posovszky P, Folsom AR, Friedrich N, Freimer NB, Fu M, Gaget S, Gejman PV, Geus EJC, Gieger C, Gjesing AP, Goel A, Goyette P, Grallert H, Gräßler J, Greenawalt DM, Groves CJ, Gudnason V, Guiducci C, Hartikainen A-L, Hassanali N, Hall AS, Havulinna AS, Hayward C, Heath AC, Hengstenberg C, Hicks AA, Hinney A, Hofman A, Homuth G, Hui J, Igl W, Iribarren C, Isomaa B, Jacobs KB, Jarick I, Jewell E, John U, Jørgensen T, Jousilahti P, Jula A, Kaakinen M, Kajantie E, Kaplan LM, Kathiresan S, Kettunen J, Kinnunen L, Knowles JW, Kolcic I, König IR, Koskinen S, Kovacs P, Kuusisto J, Kraft P, Kvaløy K, Laitinen J, Lantieri O, Lanzani C, Launer LJ, Lecoeur C, Lehtimäki T, Lettre G, Liu J, Lokki M-L, Lorentzon M, Luben RN, Ludwig B, Manunta P, Marek D, Marre M, Martin NG, McArdle WL, McCarthy A, McKnight B, Meitinger T, Melander O, Meyre D, Midthjell K, Montgomery GW, Morken MA, Morris AP, Mulic R, Ngwa JS, Nelis M, Neville MJ, Nyholt DR, O’Donnell CJ, O’Rahilly S, Ong KK, Oostra B, Paré G, Parker AN, Perola M, Pichler I, Pietiläinen KH, Platou CGP, Polasek O, Pouta A, Rafelt S, Raitakari O, Rayner NW, Ridderstråle M, Rief W, Ruokonen A, Robertson NR, Rzehak P, Salomaa V, Sanders AR, Sandhu MS, Sanna S, Saramies J, Savolainen MJ, Scherag S, Schipf S, Schreiber S, Schunkert H, Silander K, Sinisalo J, Siscovick DS, Smit JH, Soranzo N, Sovio U, Stephens J, Surakka I, Swift AJ, Tammesoo M-L, Tardif J-C, Teder-Laving M, Teslovich TM, Thompson JR, Thomson B, Tönjes A, Tuomi T, van Meurs JBJ, van Ommen G-J, Vatin V, Viikari J, Visvikis-Siest S, Vitart V, Vogel CIG, Voight BF, Waite LL, Wallaschofski H, Walters GB, Widen E, Wiegand S, Wild SH, Willemsen G, Witte DR, Witteman JC, Xu J, Zhang Q, Zgaga L, Ziegler A, Zitting P, Beilby JP, Farooqi IS, Hebebrand J, Huikuri HV, James AL, Kähönen M, Levinson DF, Macciardi F, Nieminen MS, Ohlsson C, Palmer LJ, Ridker PM, Stumvoll M, Beckmann JS, Boeing H, Boerwinkle E, Boomsma DI, Caulfield MJ, Chanock SJ, Collins FS, Cupples LA, Smith GD, Erdmann J, Froguel P, Grönberg H, Gyllensten U, Hall P, Hansen T, Harris TB, Hattersley AT, Hayes RB, Heinrich J, Hu FB, Hveem K, Illig T, Jarvelin M-R, Kaprio J, Karpe F, Khaw K-T, Kiemeney LA, Krude H, Laakso M, Lawlor DA, Metspalu A, Munroe PB, Ouwehand WH, Pedersen O, Penninx BW, Peters A, Pramstaller PP, Quertermous T, Reinehr T, Rissanen A, Rudan I, Samani NJ, Schwarz PEH, Shuldiner AR, Spector TD, Tuomilehto J, Uda M, Uitterlinden A, Valle TT, Wabitsch M, Waeber G, Wareham NJ, Watkins H, Wilson JF, Wright AF, Zillikens MC, Chatterjee N, McCarroll SA, Purcell S, Schadt EE, Visscher PM, Assimes TL, Borecki IB, Deloukas P, Fox CS, Groop LC, Haritunians T, Hunter DJ, Kaplan RC, Mohlke KL, O’Connell JR, Peltonen L, Schlessinger D, Strachan DP, van Duijn CM, Wichmann H-E, Frayling TM, Thorsteinsdottir U, Abecasis GR, Barroso I, Boehnke M, Stefansson K, North KE, I McCarthy M, Hirschhorn JN, Ingelsson E, Loos RJF (2010). Association analyses of 249,796 individuals reveal eighteen new loci associated with body mass index. Nature Genetics.

[ref-255] Srisai D, Gillum MP, Panaro BL, Zhang X-M, Kotchabhakdi N, Shulman GI, Ellacott KLJ, Cone RD (2011). Characterization of the hyperphagic response to dietary fat in the MC4R knockout mouse. Endocrinology.

[ref-256] Stahl EA, Wegmann D, Trynka G, Gutierrez-Achury J, Do R, Voight BF, Kraft P, Chen R, Kallberg HJ, Kurreeman FAS, Kathiresan S, Wijmenga C, Gregersen PK, Alfredsson L, Siminovitch KA, Worthington J, de Bakker PIW, Raychaudhuri S, Plenge RM (2012). Bayesian inference analyses of the polygenic architecture of rheumatoid arthritis. Nature Genetics.

[ref-257] Stanford WL, Cohn JB, Cordes SP (2001). Gene-trap mutagenesis: past, present and beyond. Nature Reviews. Genetics.

[ref-258] Stäubert C, Tarnow P, Brumm H, Pitra C, Gudermann T, Grüters A, Schöneberg T, Biebermann H, Römpler H (2007). Evolutionary aspects in evaluating mutations in the melanocortin 4 receptor. Endocrinology.

[ref-259] Stone S, Abkevich V, Hunt SC, Gutin A, Russell DL, Neff CD, Riley R, Frech GC, Hensel CH, Jammulapati S, Potter J, Sexton D, Tran T, Gibbs D, Iliev D, Gress R, Bloomquist B, Amatruda J, Peter Rae MM, Ted Adams D, Mark Skolnick H, Shattuck D (2002). A major predisposition locus for severe obesity at 4p15-p14. American Journal of Human Genetics.

[ref-260] Stone S, Abkevich V, Russell DL, Riley R, Timms K, Tran T, Trem D, Frank D, Jammulapati S, Neff CD, Iliev D, Gress R, He G, Frech GC, Adams TD, Skolnick MH, Lanchbury JS, Gutin A, Hunt SC, Shattuck D (2006). TBC1D1 is a candidate for a severe obesity gene evidence for a gene/gene interaction in obesity predisposition. Human Molecular Genetics.

[ref-261] Strachan T, Read A (1999). Genetic manipulation of animals. Human molecular genetics.

[ref-262] Stratigopoulos G, Leduc CA, Cremona ML, Chung WK, Leibel RL (2011). Cut-like homeobox 1 (CUX1) regulates expression of the fat mass and obesity-associated and retinitis pigmentosa GTPase regulator-interacting protein-1-like (RPGRIP1L) genes and coordinates leptin receptor signaling. The Journal of Biological Chemistry.

[ref-263] Stratigopoulos G, Martin Carli JF, O’Day DR, Wang L, LeDuc CA, Lanzano P, Chung WK, Rosenbaum M, Egli D, Doherty DA, Leibel RL (2014). Hypomorphism for RPGRIP1L, a ciliary gene vicinal to the FTO locus, causes increased adiposity in mice. Cell Metabolism.

[ref-264] Stutzmann F, Tan K, Vatin V, Dina C, Jouret B, Tichet J, Balkau B, Potoczna N, Horber F, O’Rahilly S, Farooqi IS, Froguel P, Meyre D (2008). Prevalence of melanocortin-4 receptor deficiency in europeans their age-dependent penetrance in multigenerational pedigrees. Diabetes.

[ref-265] Stutzmann F, Vatin V, Cauchi S, Morandi A, Jouret B, Landt O, Tounian P, Levy-Marchal C, Buzzetti R, Pinelli L, Balkau B, Horber F, Bougneres P, Froguel P, Meyre D (2007). Non-synonymous polymorphisms in melanocortin-4 receptor protect against obesity: the two facets of a Janus obesity gene. Human Molecular Genetics.

[ref-266] Stylianou IM, Christians JK, Keightley PD, Bünger L, Clinton M, Bulfield G, Horvat S (2004). Genetic complexity of an obesity QTL (Fob3) revealed by detailed genetic mapping. Mammalian Genome: Official Journal of the International Mammalian Genome Society.

[ref-267] Styrkarsdottir U, Thorleifsson G, Helgadottir HT, Bomer N, Metrustry S, Bierma-Zeinstra S, Strijbosch AM, Evangelou E, Hart D, Stefansson K (2014). Severe osteoarthritis of the hand associates with common variants within the ALDH1A2 gene and with rare variants at 1p31. Nature Genetics.

[ref-268] Sun N, Abil Z, Zhao H (2012). Recent advances in targeted genome engineering in mammalian systems. Biotechnology Journal.

[ref-269] Suzuki W, Iizuka S, Tabuchi M, Funo S, Yanagisawa T, Kimura M, Sato T, Endo T, Kawamura H (1999). A new mouse model of spontaneous Diabetes derived from ddY strain. Experimental Animals.

[ref-270] Svenson K, Bogue M, Peters L (2003). Invited review: identifying new mouse models of cardiovascular disease: a review of high-throughput screens of mutagenized and inbred strains. Journal of Applied Physiology.

[ref-271] Switzer NJ, Mangat HS, Karmali S (2013). Current trends in obesity: body composition assessment, weight regulation, and emerging techniques in managing severe obesity. Journal of Interventional Gastroenterology.

[ref-272] Takeshita S, Suzuki T, Kitayama S, Moritani M (2012). Bhlhe40, a potential diabetic modifier gene on Dbm1 locus , negatively controls myocyte fatty acid oxidation. Genes & Genetic Systems.

[ref-273] Tartaglia LA, Dembski M, Weng X, Deng N, Culpepper J, Devos R, Richards GJ, Campfield LA, Clark FT, Deeds J, Muir C, Sanker S, Moriarty A, Moore KJ, Smutko JS, Mays GG, Wool EA, Monroe CA, Tepper RI (1995). Identification expression of a leptin receptor OB-R. Cell.

[ref-274] Teare MD, Barrett JH, Road BH, Sheffield S (2005). Genetic Epidemiology 2 Genetic linkage studies. Lancet.

[ref-275] Terauchi Y, Matsui J, Suzuki R, Kubota N, Komeda K, Aizawa S, Eto K, Kimura S, Nagai R, Tobe K, Lienhard GE, Kadowaki T (2003). Impact of genetic background ablation of insulin receptor substrate (IRS)-3 on IRS-2 knock-out mice. The Journal of Biological Chemistry.

[ref-276] Thomas K, Capecchi M (1987). Site-directed mutagenesis by gene targeting in mouse embroyonic stem cells. Cell.

[ref-277] Thorleifsson G, Walters GB, Gudbjartsson DF, Steinthorsdottir V, Sulem P, Helgadottir A, Styrkarsdottir U, Gretarsdottir S, Thorlacius S, Jonsdottir I, Jonsdottir T, Olafsdottir EJ, Olafsdottir GH, Jonsson T, Jonsson F, Borch-Johnsen K, Hansen T, Andersen G, Jorgensen T, Lauritzen T, Aben KK, Verbeek A, Roeleveld N, Kampman E, Yanek LR, Becker LC, Tryggvadottir L, Rafnar T, Becker DM, Gulcher J, Kiemeney LA, Pedersen O, Kong A, Thorsteinsdottir U, Stefansson K (2009). Genome-wide association yields new sequence variants at seven loci that associate with measures of obesity. Nature Genetics.

[ref-278] Toye AA, Moir L, Hugill A, Bentley L, Quarterman J, Mijat V, Hough T, Goldsworthy M, Haynes A, Hunter AJ, Browne M, Spurr N, Cox RD (2004). A new mouse model of type 2 diabetes produced by N-ethyl-nitrosourea mutagenesis is the result of a missense mutation in the glucokinase gene. Diabetes.

[ref-279] Tung Y-CL, Ayuso E, Shan X, Bosch F, O’Rahilly S, Coll AP, Yeo GSH (2010). Hypothalamic-specific manipulation of Fto, the ortholog of the human obesity gene FTO, affects food intake in rats. PLoS ONE.

[ref-280] Urnov FD, Rebar EJ, Holmes MC, Zhang HS, Gregory PD (2010). Genome editing with engineered zinc finger nucleases. Nature Reviews. Genetics.

[ref-281] Vaisse C, Clement K, Guy-grand B, Froguel P (1998). A frameshift mutation in human MC4R is associated with a dominant form of obesity WRN , is a 3′ → 5′ exonuclease. Nature Genetics.

[ref-282] Valdar W, Solberg LC, Gauguier D, Burnett S, Klenerman P, Cookson WO, Taylor MS, Rawlins J Nicholas P., Mott R, Flint J (2006). Genome-wide genetic association of complex traits in heterogeneous stock mice. Nature Genetics.

[ref-283] Vimaleswaran KS, Tachmazidou I, Zhao JH, Hirschhorn JN, Dudbridge F, Loos RJF (2012). Candidate genes for obesity-susceptibility show enriched association within a large genome-Wide association study for BMI. Human Molecular Genetics.

[ref-284] Visscher PM, Brown MA, McCarthy MI, Yang J (2012). Five years of GWAS discovery. American Journal of Human Genetics.

[ref-285] Vogel H, Scherneck S, Kanzleiter T, Benz V, Kluge R, Stadion M, Kryvych S, Bluher M, Kloting N, Joost H-G, Schurmann A (2012). Loss of function of Ifi202b by a microdeletion on chromosome 1 of C57BL/6J mice suppresses 11*β*-hydroxysteroid dehydrogenase type 1 expression development of obesity. Human Molecular Genetics.

[ref-286] Vuillaume M-L, Naudion S, Banneau G, Diene G, Cartault A, Cailley D, Bouron J, Toutain J, Bourrouillou G, Vigouroux A, Bouneau L, Nacka F, Kieffer I, Arveiler B, Knoll-Gellida A, Babin PJ, Bieth E, Jouret B, Julia S, Sarda P, Geneviève D, Faivre L, Lacombe D, Barat P, Tauber M, Delrue M-A, Rooryck C (2014). New candidate loci identified by array-CGH in a cohort of 100 children presenting with syndromic obesity. American Journal of Medical Genetics. Part A.

[ref-287] Wabitsch M, Funcke J-B, Lennerz B, Kuhnle-Krahl U, Lahr G, Debatin K-M, Vatter P, Gierschik P, Moepps B, Fischer-Posovszky P (2015). Biologically inactive leptin and early-onset extreme obesity. New England Journal of Medicine.

[ref-288] Walters RG, Jacquemont S, Valsesia A, Smith AJDE, Martinet D (2010). A novel highly-penetrant form of obesity due to microdeletions on chromosome 16p11.2. Nature.

[ref-289] Wang F, Mullican SE, Dispirito JR, Peed LC, Lazar MA (2013). Lipoatrophy and severe metabolic disturbance in mice with fat-speci fi c deletion of PPAR *γ*. Proceedings of the National Academy of Sciences of the United States of America.

[ref-290] Warden CH, Fisler JS, Pace MJ, Svenson KL, Lusis AJ (1993). Coincidence of genetic loci for plasma cholesterol levels and obesity in a multifactorial mouse model. The Journal of Clinical Investigation.

[ref-291] Warden CH, Fisler JS, Shoemaker SM, Wen PZ, Svenson KL, Pace MJ, Lusis AJ (1995). Identification of four chromosomal loci determining obesity in a multifactorial mouse model. The Journal of Clinical Investigation.

[ref-292] Wheeler E, Huang N, Bochukova EG, Keogh JM, Lindsay S, Garg S, Henning E, Blackburn H, Loos RJF, Wareham NJ, O’Rahilly S, Hurles ME, Barroso I, Farooqi IS (2013). Genome-wide SNP and CNV analysis identifies common and low-frequency variants associated with severe early-onset obesity. Nature Genetics.

[ref-293] Willer CJ, Speliotes EK, Loos RJF, Li S, Lindgren CM, Heid IM, Berndt SI, Elliott AL, Jackson AU, Lamina C, Lettre G, Lim N, Lyon HN, McCarroll SA, Papadakis K, Qi L, Randall JC, Roccasecca RM, Sanna S, Scheet P, Weedon MN, Wheeler E, Zhao JH, Jacobs LC, Prokopenko I, Soranzo N, Tanaka T, Timpson NJ, Almgren P, Bennett A, Bergman RN, Bingham SA, Bonnycastle LL, Brown M, Burtt NP, Chines P, Coin L, Collins FS, Connell JM, Cooper C, Smith GD, Dennison EM, Deodhar P, Elliott P, Erdos MR, Estrada K, Evans DM, Gianniny L, Gieger C, Gillson CJ, Guiducci C, Hackett R, Hadley D, Hall AS, Havulinna AS, Hebebrand J, Hofman A, Isomaa B, Jacobs KB, Johnson T, Jousilahti P, Jovanovic Z, Khaw K-T, Kraft P, Kuokkanen M, Kuusisto J, Laitinen J, Lakatta EG, Luan Jian’an, Luben RN, Mangino M, McArdle WL, Meitinger T, Mulas A, Munroe PB, Narisu N, Ness AR, Northstone K, O’Rahilly S, Purmann C, Rees MG, Ridderstråle M, Ring SM, Rivadeneira F, Ruokonen A, Sandhu MS, Saramies J, Scott LJ, Scuteri A, Silander K, Sims MA, Song K, Stephens J, Stevens S, Stringham HM, Tung YCL, Valle TT, Van Duijn CM, Vimaleswaran KS, Vollenweider P, Waeber G, Wallace C, Watanabe RM, Waterworth DM, Watkins N, Witteman JCM, Zeggini E, Zhai G, Zillikens MC, Altshuler D, Caulfield MJ, Chanock SJ, Farooqi IS, Ferrucci L, Guralnik JM, Hattersley AT, Hu FB, Jarvelin M-R, Laakso M, Mooser V, Ong KK, Ouwehand WH, Salomaa V, Samani NJ, Spector TD, Tuomi T, Tuomilehto J, Uda M, Uitterlinden AG, Wareham NJ, Deloukas P, Frayling TM, Groop LC, Hayes RB, Hunter DJ, Mohlke KL, Peltonen L, Schlessinger D, Strachan DP, Wichmann H-E, McCarthy MI, Boehnke M, Barroso I, Abecasis GR, Hirschhorn JN (2009). Six new loci associated with body mass index highlight a neuronal influence on body weight regulation. Nature Genetics.

[ref-294] World Health Organization (2011). Obesity and overweight: factsheet.

[ref-295] Wuschke S, Dahm S, Schmidt C, Joost H-G, Al-Hasani H (2007). A meta-analysis of quantitative trait loci associated with body weight and adiposity in mice. International Journal of Obesity.

[ref-296] Xiang K, Wang Y, Zheng T, Jia W, Li J, Chen L, Shen K, Wu S, Lin X, Zhang G, Wang C, Wang S, Lu H, Fang Q, Shi Y, Zhang R, Xu J, Weng Q (2004). Genome-wide search for type 2 diabetes/impaired glucose homeostasis susceptibility genes in the Chinese significant linkage to chromosome 6q21-q23 and chromosome 1q21-q24. Diabetes.

[ref-297] Xu B, Goulding EH, Zang K, Cepoi D, Cone RD, Jones KR, Tecott LH, Reichardt LF (2003). Brain-derived neurotrophic factor regulates energy balance downstream of melanocortin-4 receptor. Nature Neuroscience.

[ref-298] Yamamura K, Araki K (2008). Gene trap mutagenesis in mice: new perspectives and tools in cancer research. Cancer Science.

[ref-299] Yang X, Deignan JL, Qi H, Zhu J, Qian S, Zhong J, Torosyan G, Majid S, Falkard B, Kleinhanz RR, Karlsson J, Castellani LW, Mumick S, Wang K, Xie T, Coon M, Zhang C, Estrada-Smith D, Farber CR, Wang SS, van Nas A, Ghazalpour A, Zhang B, MacNeil DJ, Lamb JR, Dipple KM, Reitman ML, Mehrabian M, Lum PY, Schadt EE, Lusis AJ, Drake TA (2009). Validation of candidate causal genes for obesity that affect shared metabolic pathways and networks. Nature Genetics.

[ref-300] Yang D, Jiang Y, He F (2009). An integrated view of the correlations between genomic and phenomic variables. Journal of Genetics and Genomics.

[ref-301] Yaswen L, Diehl N, Brennan MB, Hochgeschwender U (1999). Obesity in the mouse model of pro-opiomelanocortin deficiency responds to peripheral melanocortin. Nature Medicine.

[ref-302] Yazbek SN, Buchner DA, Geisinger JM, Burrage LC, Spiezio SH, Zentner GE, Hsieh C-W, Scacheri PC, Croniger CM, Nadeau JH (2011). Deep congenic analysis identifies many strong context-dependent QTLs one of which Slc35b4 regulates obesity glucose homeostasis. Genome Research.

[ref-303] Yeo GS, Farooqi IS, Aminian S, Halsall DJ, Stanhope RG, O’Rahilly S (1998). A frameshift mutation in MC4R associated with dominantly inherited human obesity. Nature Genetics.

[ref-304] Yeo GSH, Connie Hung C-C, Rochford J, Keogh J, Gray J, Sivaramakrishnan S, O’Rahilly S, Farooqi IS (2004). A de novo mutation affecting human TrkB associated with severe obesity developmental delay. Nature Neuroscience.

[ref-305] Yi N, Diament A, Chiu S, Kim K, Allison DB, Fisler JS, Warden CH (2004). Characterization of epistasis influencing complex spontaneous obesity in the BSB model. Genetics.

[ref-306] Yin H, Kanasty RL, Eltoukhy AA, Vegas AJ, Dorkin JR, Anderson DG (2014). Non-viral vectors for gene-based therapy. Nature Reviews. Genetics.

[ref-307] Yoganathan P, Karunakaran S, Clee Ho (2012). Nutritional regulation of genome-wide association obesity genes in a tissue-dependent manner. Nutrition and Metabolism.

[ref-308] Yourshaw M, Solorzano-Vargas RS, Pickett LA, Lindberg I, Wang J, Cortina G, Pawlikowska-Haddal A, Baron H, Venick RS, Nelson SF, Martín MG (2013). Exome sequencing finds a novel PCSK1 mutation in a child with generalized malabsorptive diarrhea and diabetes insipidus. Journal of Pediatric Gastroenterology and Nutrition.

[ref-309] Zegers D, Beckers S, Hendrickx R (2013). Prevalence of rare MC3R variants in obese cases and lean controls. Endocrine.

[ref-310] Zhang Y, Proenca R, Maffei M, Barone M, Leopold L, Friedman J (1994). Positional cloning of the mouse obese gene and its human homologue. Nature.

[ref-311] Zhang F, Wen Y, Guo X (2014). CRISPR/Cas9 for genome editing: progress, implications and challenges. Human Molecular Genetics.

[ref-312] Zhu M, Zhao S (2007). Candidate gene identification approach: progress and challenges. International Journal of Biological Sciences.

[ref-313] Zhu X, Zhou A, Dey A, Norrbom C, Carroll R, Zhang C, Laurent V, Lindberg I, Ugleholdt R, Holst JJ, Steiner DF (2002). Disruption of PC1/3 expression in mice causes dwarfism multiple neuroendocrine peptide processing defects. Proceedings of the National Academy of Sciences of the United States of America.

[ref-314] Zou F, Gelfond JAL, Airey DC, Lu L, Manly KF, Williams RW, Threadgill DW (2005). Quantitative trait locus analysis using recombinant inbred intercrosses: theoretical and empirical considerations. Genetics.

